# Supplementary material for: A Role in 15-Deacetylcalonectrin Acetylation in the Non-Enzymatic Cyclization of an Earlier Bicyclic Intermediate in Fusarium Trichothecene Biosynthesis
Source: Int J Mol Sci. 2024 Apr 12;25(8):4288. doi: 10.3390/ijms25084288 (PMC11050026; doi:10.3390/ijms25084288)
Supplement: Supplementary file 1 [file ijms-25-04288-s001.zip › ijms-2953346-supplementary.pdf]

A role in 15-deacetylcalonectrin acetylation in the non-enzymatic cyclization of an earlier bicyclic intermediate in *Fusarium* trichothecene biosynthesis

Yoshiaki Koizumi[1], Yuichi Nakajima[2], Yuya Tanaka[2], Kosuke Matsui[2], Masato Sakabe[3], Kazuyuki Maeda[2], Masayuki Sato[4], Hiroyuki Koshino[5], Soichi Sato[1,3], Makoto Kimura[2,4], Naoko Takahashi-Ando[1,3,4]

[1]Graduate School of Science and Engineering, Toyo University, 2100 Kujirai, Kawagoe, Saitama 350-8585, Japan

[2]Graduate School of Bioagricultural Sciences, Nagoya University, Furo-cho, Chikusa-ku, Nagoya, Aichi 464-8601, Japan

[3]Faculty of Science and Engineering, Toyo University, 2100 Kujirai, Kawagoe, Saitama 350-8585, Japan

[4]Plant & Microbial Engineering Research Unit, Discovery Research Institute (DRI) RIKEN, 2-1, Hirosawa, Wako, Saitama 351-0198, Japan

[5]Molecular Structure Characterization Unit, Technology Platform Division, Center for Sustainable Resource Science (CSRS) RIKEN, 2-1, Hirosawa, Wako, Saitama 351-0198, Japan

Table S1. Primers used for generation of the Δ*Fgtri3* (FGD3) and Δ*Fgtri5*Δ*Fgtri11* (FGD5/11) mutants

No.	Primer name	Primer sequence	Comments
#1	MFTri3-LAU	5′-TATCTTTACCACAACAGCCGATTGCCCC-3′	Primer for screening of the *FgTri3* gene disruption mutant (used in combination with #2)
#2	MFTri3-LAD	5′-GCACTCTCTGATTGGAGGAGAACACTTG-3′	Primer for screening of the *FgTri3* gene disruption mutant (used in combination with #1)
#3	MFTri3U-Sna	5′-GTACGTAGTCATTGTCTCCATTATAACAAGGC-3′	Inward primer containing a *Sna*BI site (underlined) used for the construction of the *FgTri3* gene disruption vector (used in combination with #4)
#4	MFTri3D-Sna	5′-TTTACGTATGCAGGAATGGAAGTTCATTGTAG-3′	Inward primer containing a *Sna*BI site (underlined) used for the construction of the *FgTri3* gene disruption vector (used in combination with #3)
#5	MFTri3U-inv/Spe	5′-AACTAGTATGGGGCCAAGAGATCAAGAACCTG-3′	Outward primer containing a *Spe*I site (underlined) used for the construction of the *FgTri3* gene disruption vector (used in combination with #6)
#6	MFTri3D-inv/Not	5′-AGCGGCCGCTTGAGGGATTGGCGTCGAGTCTG-3′	Outward primer containing a *Not*I site (underlined) used for the construction of the *FgTri3* gene disruption vector (used in combination with #5)
#7	MFTri11-LAU2	5′-TCAAGGCTCTCCCTGGGATG-3′	Primer for PCR confirmation of *FgTri11* gene disruption (used in combination with #8)
#8	MFTri11-LAD	5′- ATGAGTCGATCAATTTAGCGGCTGCGAG-3′	Primer for PCR confirmation of *FgTri11* gene disruption (used in combination with #7)

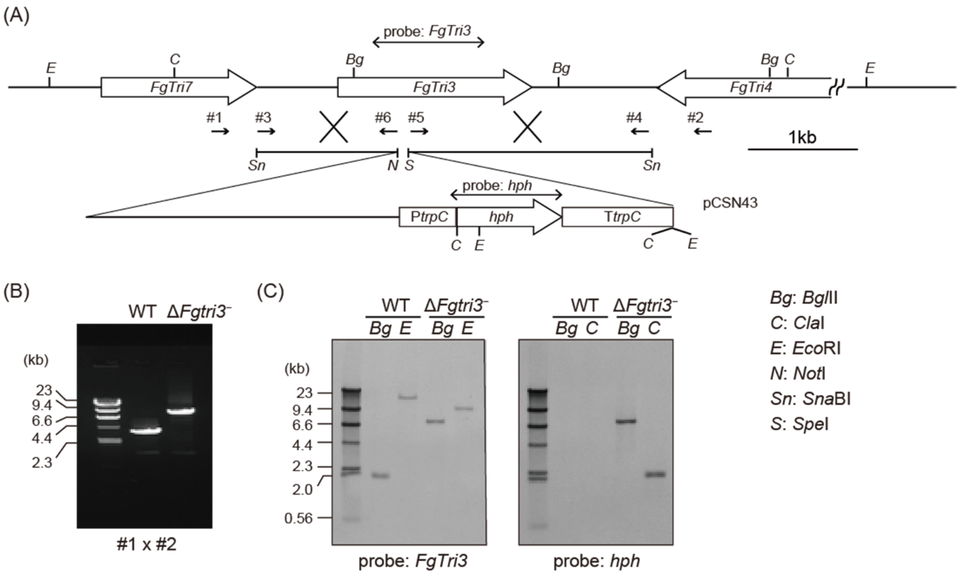

Supplementary Figure S1. Generation of a Δ*Fgtri3* mutant used in this study. (A) The gene disruption vector pΔFgTri3-hph was constructed by replacing a portion of an *FgTri3* coding region with pCSN43 [1] that contains the *hph* (hygromycin B phosphotransferase gene) cassette by the inverse polymerase chain reaction (IPCR) method [2] as follows: (1) *FgTri3* and its flanking regions were amplified by long PCR with inward primers #3 and #4 (Table S1), both of which had a *Sna*BI recognition site (Table S1); (2) the amplified product was self-ligated after digestion with *Sna*BI; (3) the ligated flanking regions were amplified by IPCR with the outward primers #5 and #6 (Table S1) that had *Spe*I and *Not*I sites, respectively (Table S1); and (4) the IPCR product was cloned between *Spe*I and *Not*I sites downstream of the *trpC* terminator. After transformation, the colonies were selected with 300 μg/ml of hygromycin B. (B) PCR analysis of genomic DNA isolated from the wild-type (WT) and Δ*Fgtri3* mutant strains. Two primers #1 and #2 that amplify the region encompassing *FgTri3* were used. An amplicon from the disruption mutant is 4.4 kb larger than the one from the WT strain. (C) Southern blot analysis of genomic DNA isolated from the WT and Δ*Fgtri3* mutant strains. The probes were synthesized with a PCR DIG probe synthesis kit [3] and detected with a DIG Luminescent Detection kit (Roche Diagnostics GmbH, Mannheim); standard hybridization, washing, and detection conditions provided by the manufacture were used for detection. Either blot showed expected banding patterns with probes that correspond to *FgTri3* and *hph*.

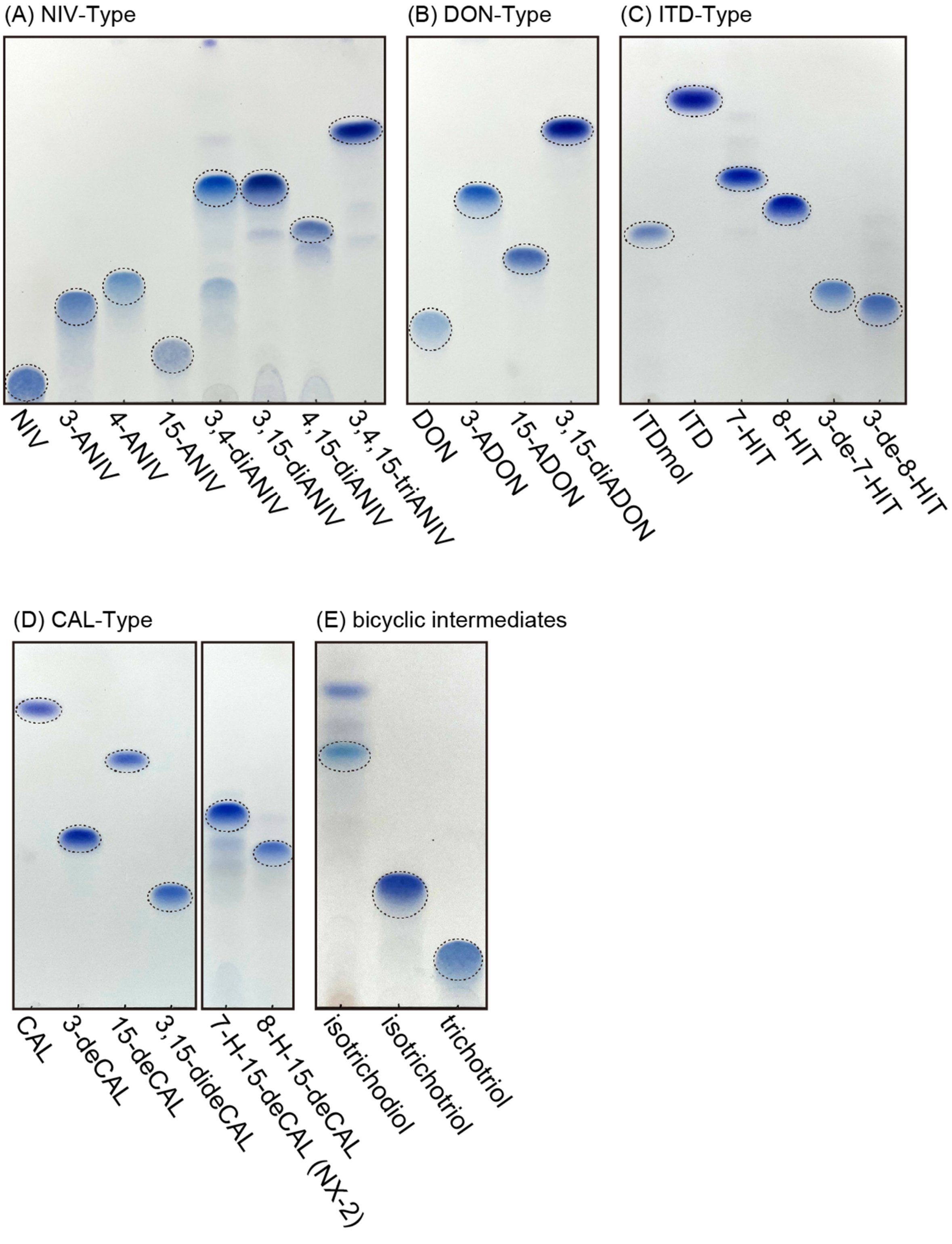

Supplementary Figure S2. Trichothecene standards on TLC. (A) NIV-type trichothecenes. NIV was produced by the deacetylation of 4-acetylnivalenol (4-ANIV). 4-ANIV and 4,15-diacetylnivalenol (4,15-diANIV) were produced through the incubation of MAFF 111233 WT culture. 3-Acetylnivalenol (3-ANIV) was produced through the incubation of the culture of

NBRC 113183, an *Fgtri13*_o/e strain of *F. graminearum* MAFF 101551 [4]. 15-Acetylnivalenol (15-ANIV) and 3,15-diacetylnivalenol (3,15-diANIV) were produced through the incubation of the culture of NBRC 113182, an *Fgtri7*$^{-}$ #1 s1* strain of *F. graminearum* MAFF 111233 [4]. 3,4,15-Triacetylnivalenol (3,4,15-triANIV) was produced through the incubation of the culture of NBRC 114123, an *FgTri8* disruptant of *F. graminearum* MAFF 111233 [5]. 3,4-Diacetylnivalenol (3,4-diANIV) was obtained by 3-*O*-acetylation of 4-ANIV by Tri101p. (B) DON-type trichothecenes. 3,15-diADON was produced through the incubation of the culture of YN_149. (C) ITD-type trichothecenes. 3-Deacetyl-7-hydroxyisotrichodermin (3-de-7-HIT) and 3-deacetyl-8-hydroxyisotrichodermin (3-de-8-HIT) were obtained through the alkaline deacetylation of 7-HIT and 8-HIT, respectively. (D) CAL-type trichothecenes. 3,15-dideCAL is the abbreviation for 3,15-dideacetylcalonectrin. 7-H-15-deCAL was obtained through the incubation of the culture of *F. graminearum* Δ*Fgtri1*/*FgTri1*$_{\_NX\ chemotype}$ [6]. Another name for 7-H-15-deCAL is NX-2. 8-H-15-deCAL was obtained through the incubation of FGD3 culture, and its structure was confirmed by NMR analysis (Supplementary Figure S3, spot 3). (E) Bicyclic trichothecene intermediates. Isotrichodiol, isotrichotriol and trichotriol were produced through the incubation of FGD3 culture, and their structures were confirmed by NMR analysis (Supplementary Figure S3, spots 1, 3, and 4).

(A)

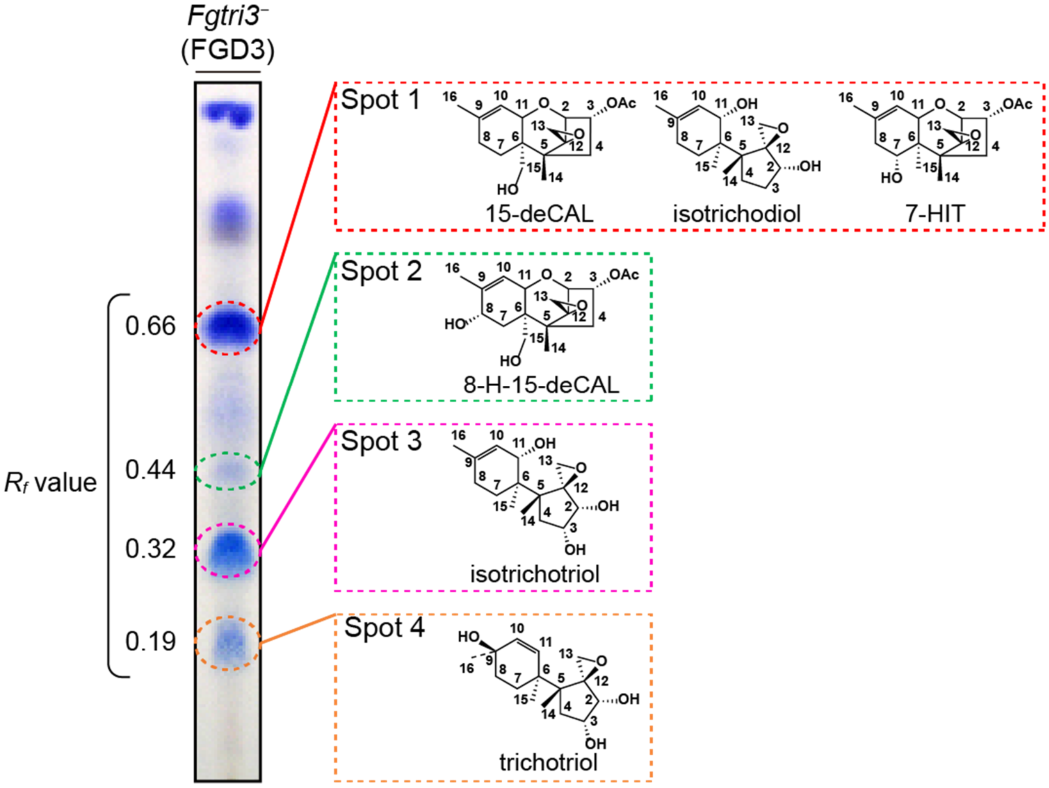

(B)

Spot 1 ：15-deCAL [^{1}H NMR Data of 15-deCAL (600 MHz, $CDCl_3$)]

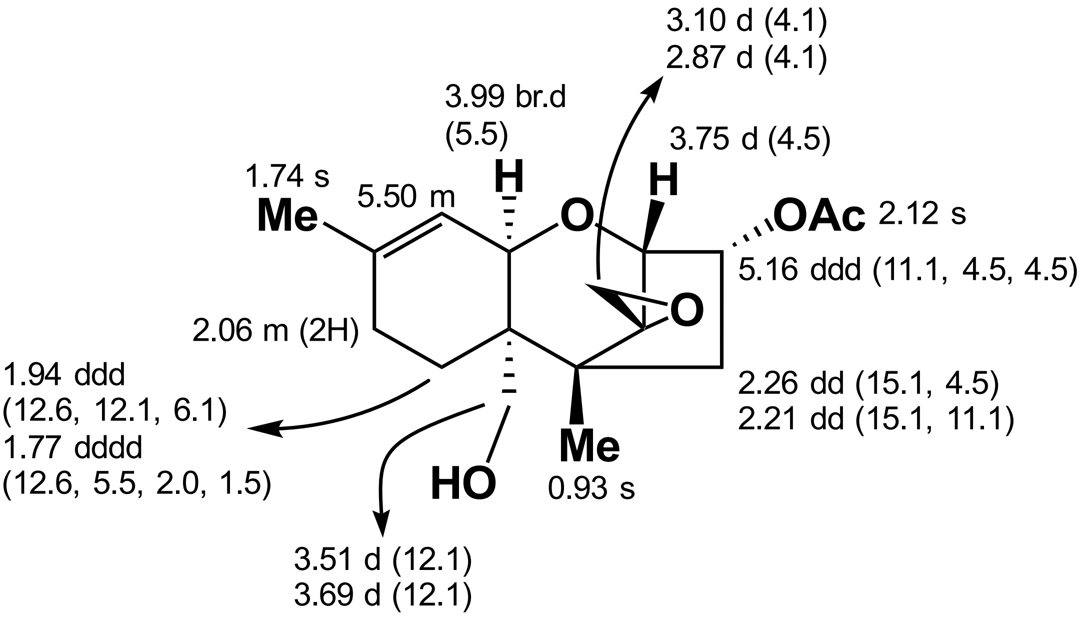

^{13}C NMR Data of 15-deCAL (151 MHz, $CDCl_3$) 78.1 (C-2), 71.4 (C-3), 39.3 (C-4), 45.3 (C-5), 44.2 (C-6), 20.8 (C-7), 28.4 (C-8), 140.5 (C-9), 119.3 (C-10), 68.3 (C-11), 65.2 (C-12), 48.6 (C-13), 12.5 (C-14), 62.8 (C-15), 23.3 (C-16), 21.0, and 170.5 (3-OAc) ppm.

Spot 1 : isotrichodiol [^{1}H NMR Data of isotrichodiol (600 MHz, $CDCl_3$)]

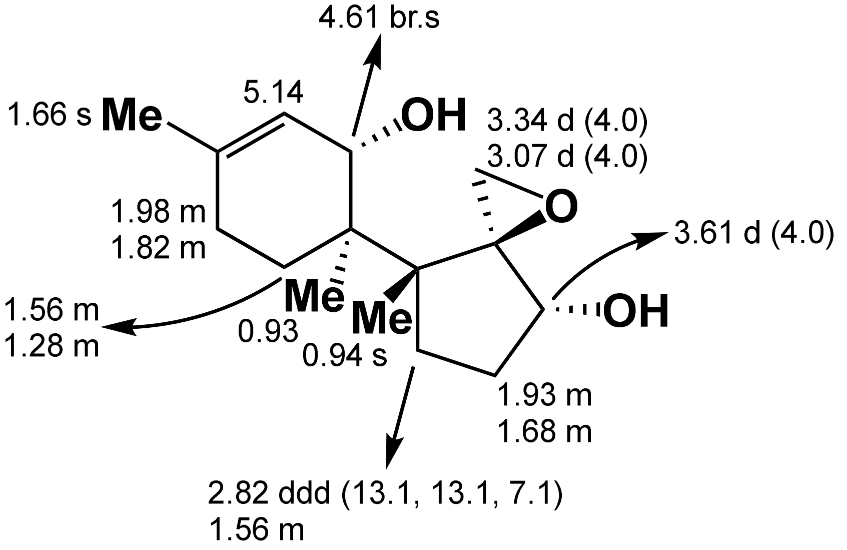

^{13}C NMR Data of isotrichodiol (151 MHz, $CDCl_3$) 81.7 (C-2), 30.3 (C-3), 37.1 (C-4), 46.8 (C-5), 41.0 (C-6), 29.2 (C-7), 27.8 (C-8), 130.5 (C-9), 125.5 (C-10), 72.0 (C-11), 69.3 (C-12), 49.5 (C-13), 20.2 (C-14), 13.0 (C-15), and 22.3 (C-16) ppm.

Spot 1 : 7-HIT [^{1}H NMR Data of 7-HIT (600 MHz, $CDCl_3$)]

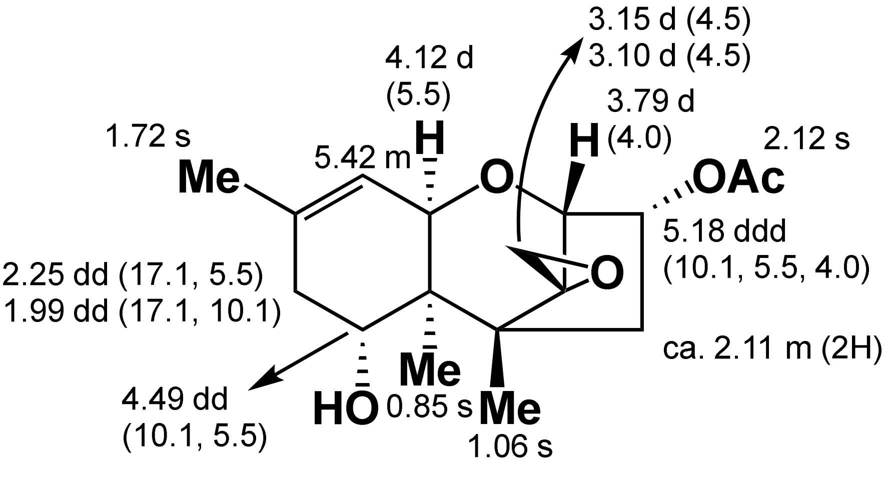

^{13}C NMR Data of 7-HIT (151 MHz, $CDCl_3$) 79.0 (C-2), 71.4 (C-3), 41.5 (C-4), 46.2 (C-5), 44.7 (C-6), 69.1 (C-7), 39.6 (C-8), 137.9 (C-9), 119.5 (C-10), 74.7 (C-11), 65.0 (C-12), 47.7 (C-13), 15.1 (C-14), 10.3 (C-15), and 22.5 (C-16) ppm.

Spot 2：8-H-15-deCAL [^{1}H NMR Data of 8-H-15-deCAL (600 MHz, $CDCl_3$)]

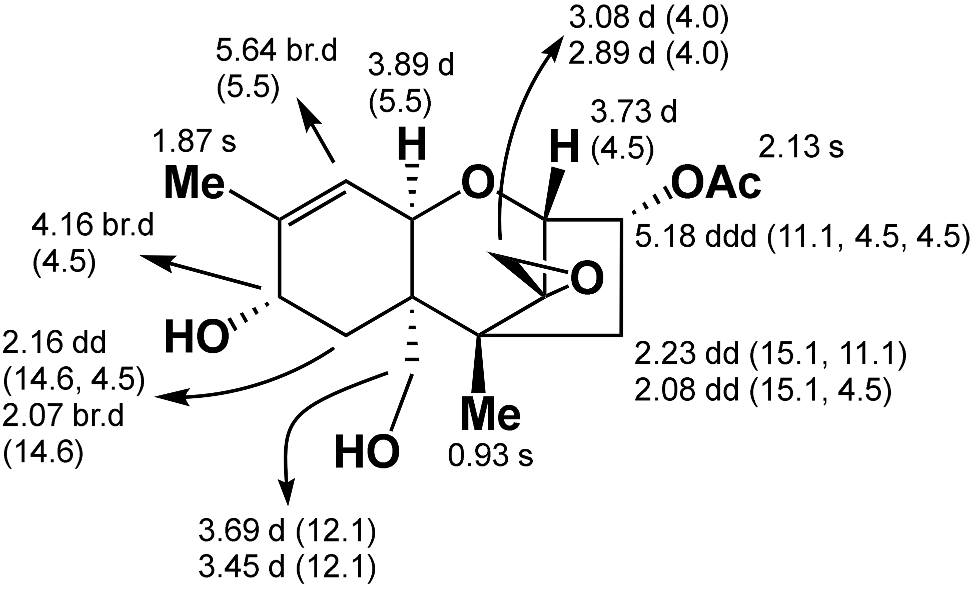

^{13}C NMR Data of 8-H 15-deCAL (151 MHz, $CDCl_3$) 77.9 (C-2), 71.3 (C-3), 39.5 (C-4), 45.7 (C-5), 44.0 (C-6), 28.3 (C-7), 67.3 (C-8), 138.8 (C-9), 122.8 (C-10), 68.6 (C-11), 67.0 (C-12), 48.5 (C-13), 12.6 (C-14), 62.7 (C-15), 20.6 (C-16), 21.1, and 170.7 (3-OAc) ppm.

Spot 3：isotrichotriol [^{1}H NMR Data of isotrichotriol (600 MHz, $CDCl_3$)]

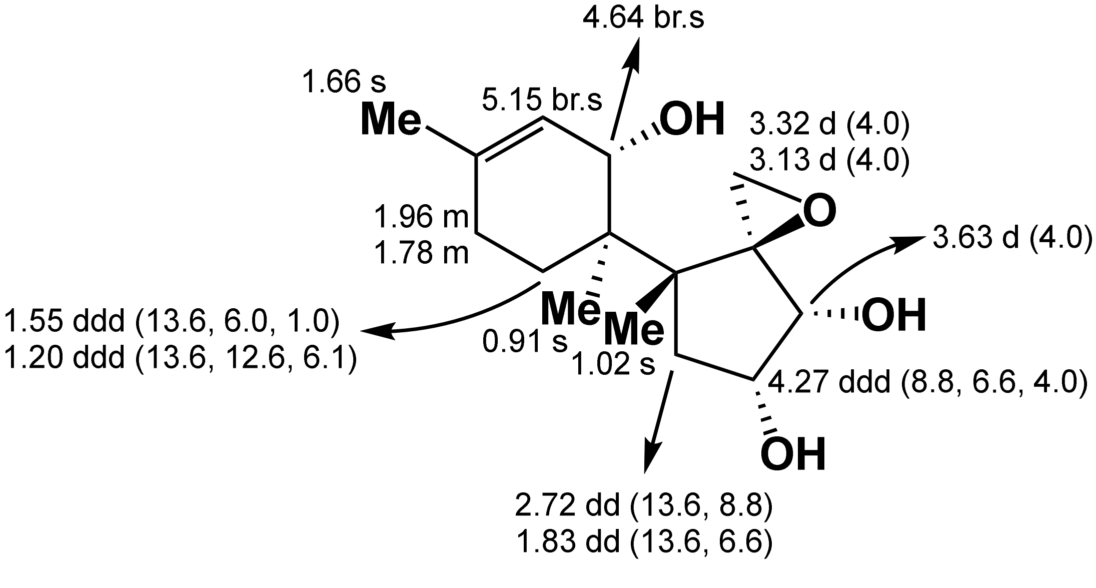

^{13}C NMR Data of isotrichotriol (151 MHz, $CDCl_3$) 79.6 (C-2), 70.0 (C-3), 43.5 (C-4), 45.2 (C-5), 41.2 (C-6), 29.1 (C-7), 27.6 (C-8), 135.3 (C-9), 125.3 (C-10), 71.5 (C-11), 67.5 (C-12), 49.3 (C-13), 22.0 (C-14), 13.1 (C-15), and 22.5 (C-16) ppm.

Spot 4：trichotriol [^{1}H NMR Data of trichotriol (600 MHz, $CDCl_3$)]

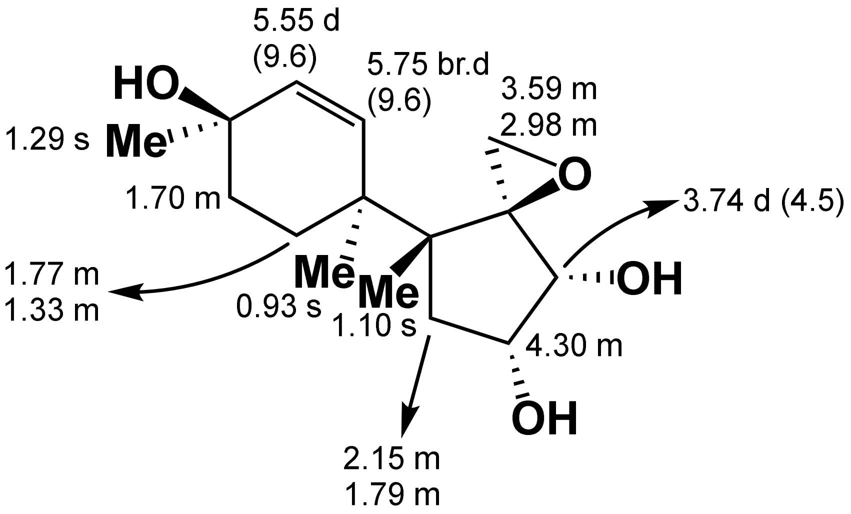

^{13}C NMR Data of trichotriol (151 MHz, $CDCl_3$) 77.5 (C-2), 69.0 (C-3), 41.5 (C-4), 43.9 (C-5), 39.5 (C-6), 27.0 (C-7), 34.0 (C-8), 66.5 (C-9), 131.3 (C-10), 136.3 (C-11), 69.2 (C-12), 49.9 (C-13), 20.4 (C-14), 21.3 (C-15), and 30.7 (C-16) ppm.

Supplementary Figure S3. Bicyclic precursors and tricyclic trichothecene intermediates of FGD3. (A) TLC analysis of trichothecene metabolites. Spot 1 contained 15-deCAL, isotrichodiol, and 7-HIT. Spots 2, 3, and 4 contained 8-H-15-deCAL, isotrichotriol, and trichotriol, respectively. (B) The compounds in spots 1–4 were confirmed by NMR analyses.

(A) ITDmol $[M+H]^+$ *m/z*: 251.164, RT: 3.88 min

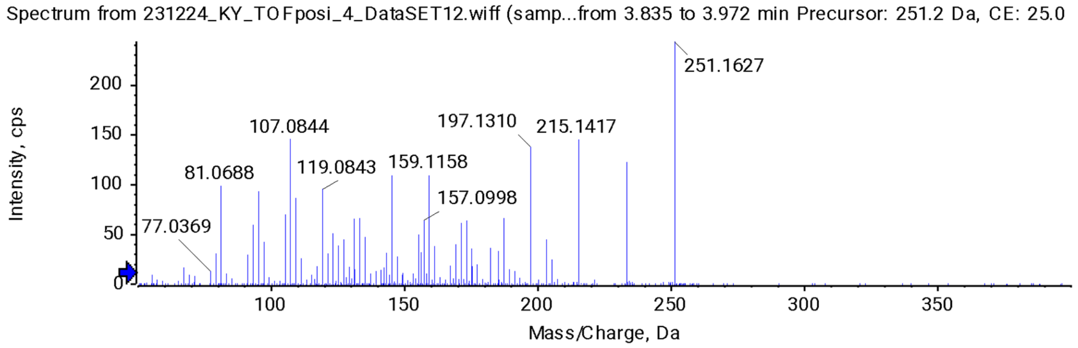

(B) ITD $[M+H]^+$ *m/z*: 293.175, RT: 4.95 min

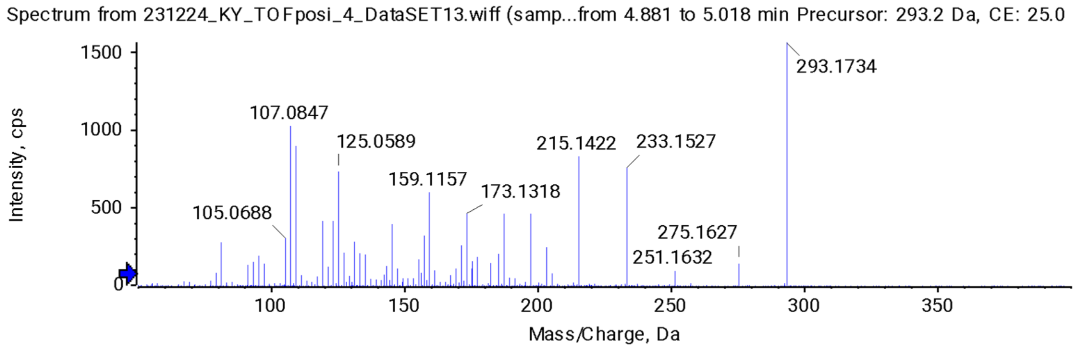

(C-1) 7-HIT $[M+H]^+$ *m/z*: 309.170, RT: 3.84 min

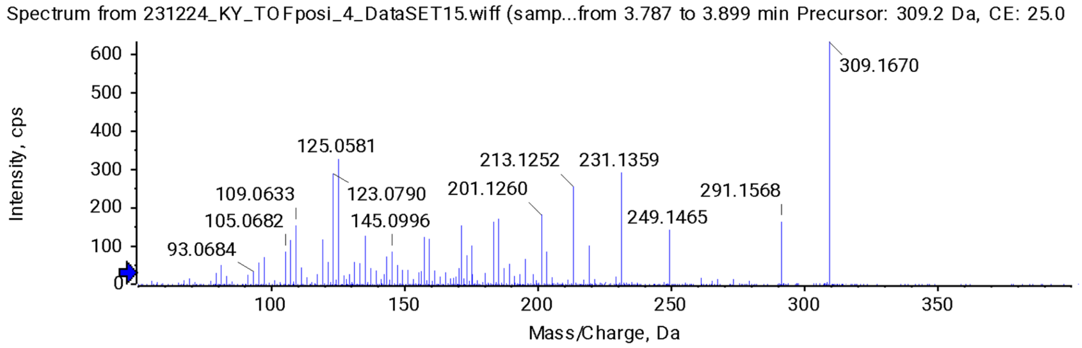

(C-2) 7-HIT $[M+NH_4]^+$ *m/z*: 326.196, RT: 3.84 min

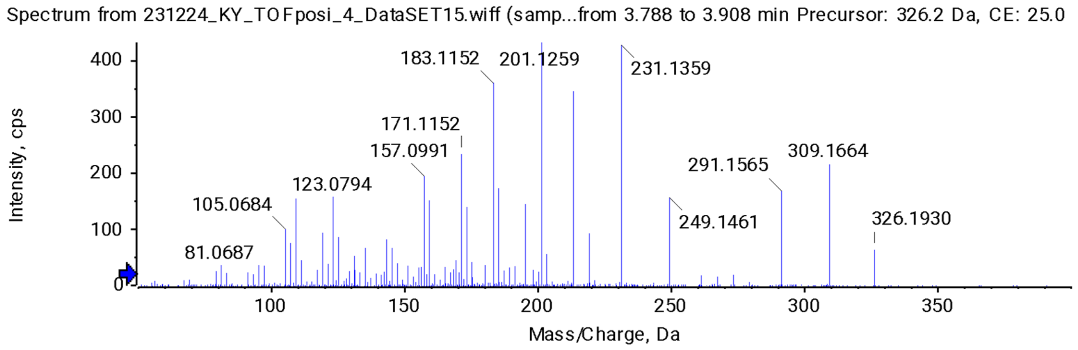

(D-1) 8-HIT $[M+H]^+$ *m/z*: 309.170, RT: 3.45 min

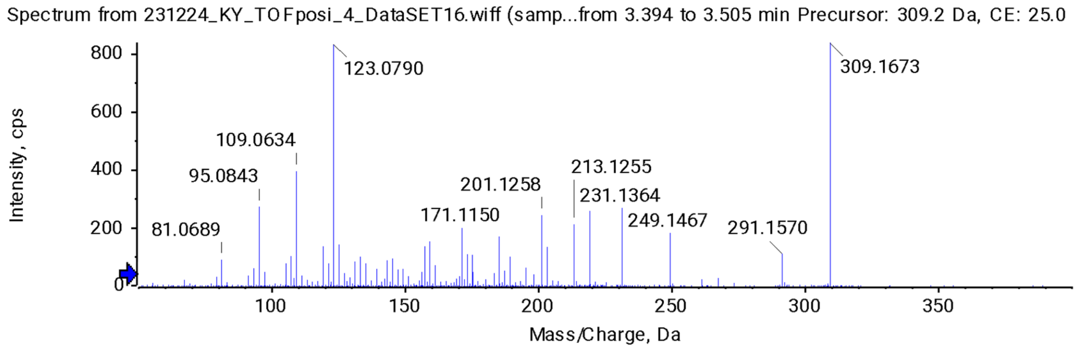

(D-2) 8-HIT $[M+NH_4]^+$ *m/z*: 326.196, RT: 3.44 min

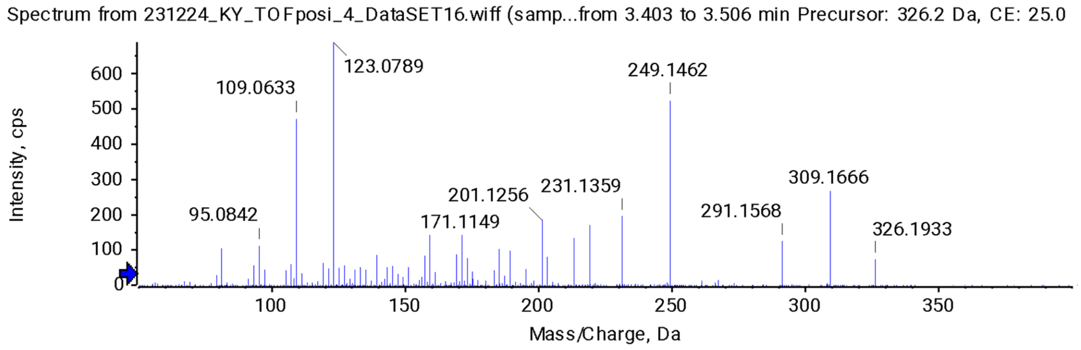

(E-1) 15-deCAL $[M+H]^+$ *m/z*: 309.170, RT: 3.55 min

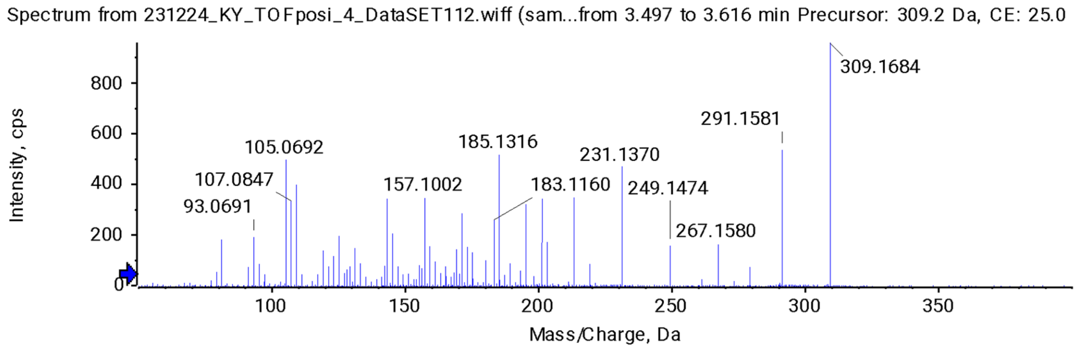

(E-2) 15-deCAL $[M+NH_4]^+$ *m/z*: 326.196, RT: 3.54 min

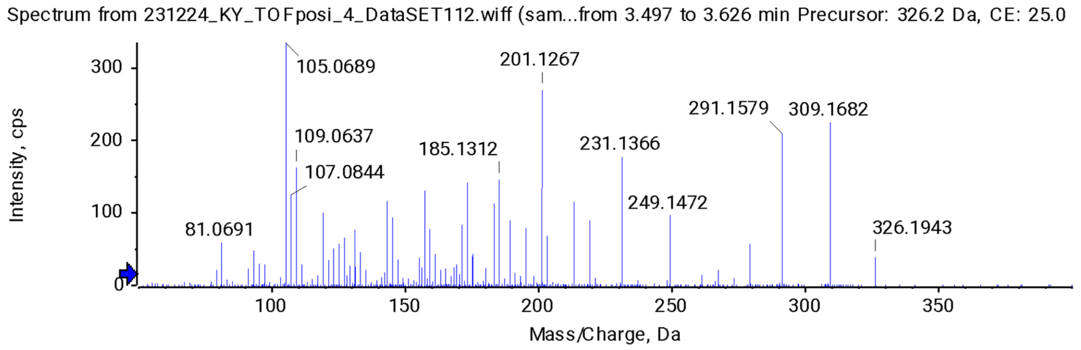

(F) CAL $[M+NH_4]^+$ *m/z*: 368.207, RT: 4.41 min

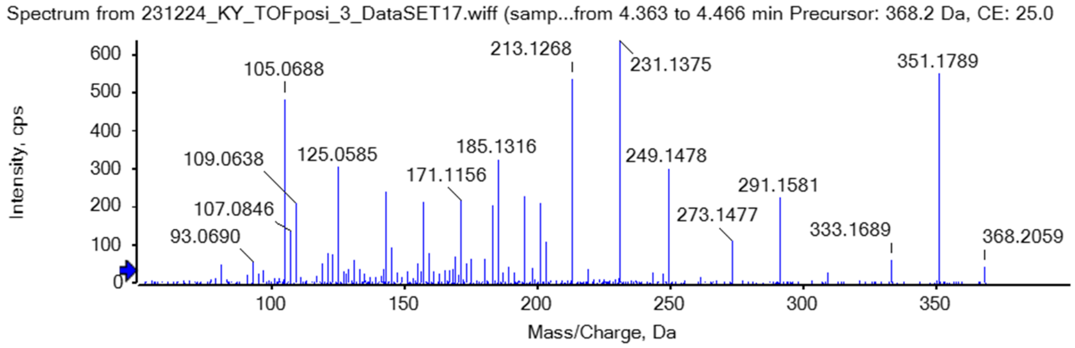

(G) 3-deCAL $[M+NH_4]^+$ *m/z*: 326.196, RT: 3.58 min

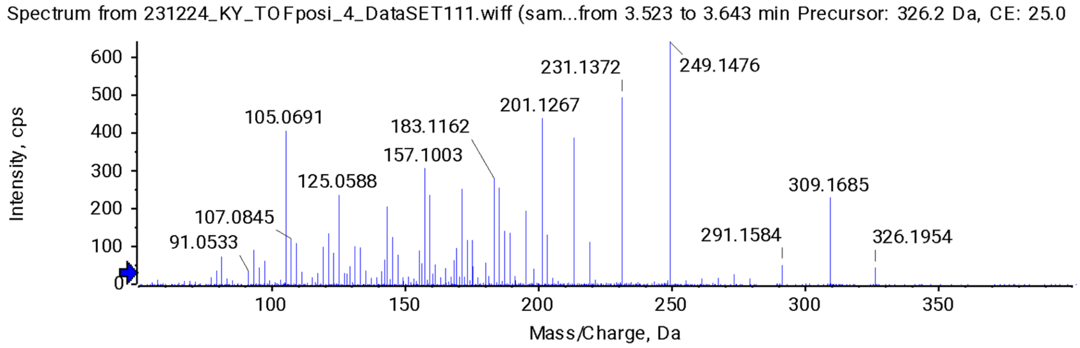

(H) isotrichotriol $[M+NH_4]^+$ *m/z*: 286.201, RT: 3.45 min

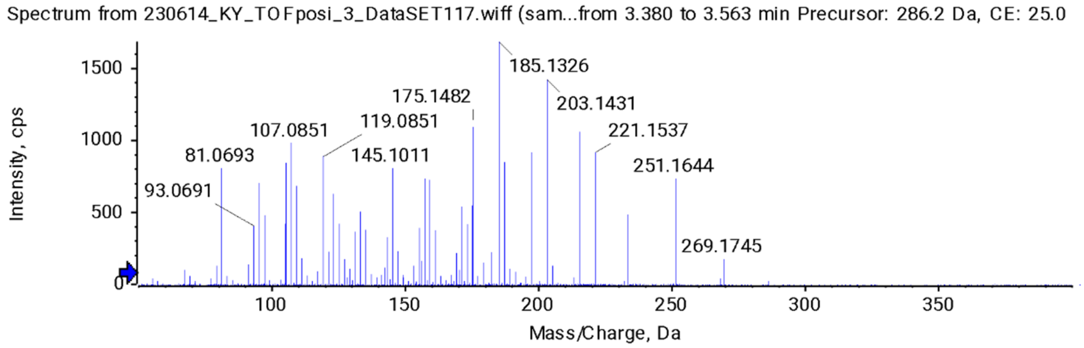

(I) trichotriol $[M+NH_4]^+$ *m/z*: 286.201, RT: 3.04 min

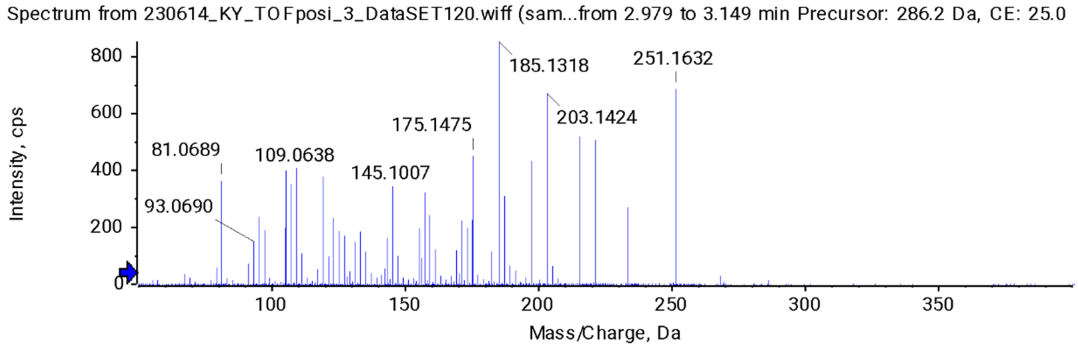

(J) 3-deacetyl-7-hydroxyisotrichodermin (3-de-7-HIT) $[M+H]^+$ *m/z*: 267.159, RT: 2.80 min

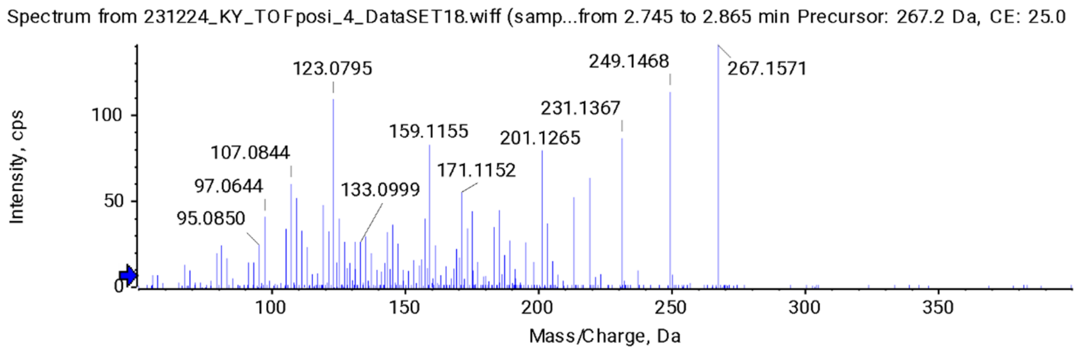

(K) 3-deacetyl-8-hydroxyisotrichodermin (3-de-8-HIT) $[M+NH_4]^+$ *m/z*: 284.186, RT: 2.48 min

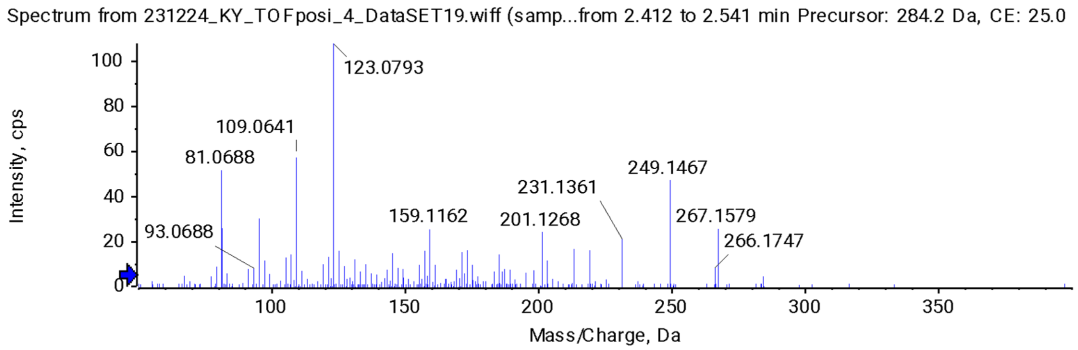

(L) 7-H-15-deCAL (NX-2) $[M+NH_4]^+$ *m/z*: 342.191, RT: 3.15 min

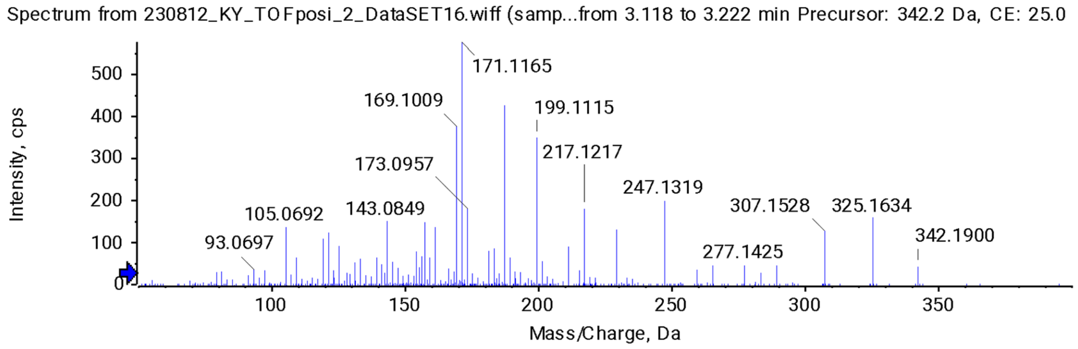

(M) 8-H-15-deCAL $[M+NH_4]^+$ *m/z*: 342.191, RT: 3.10 min

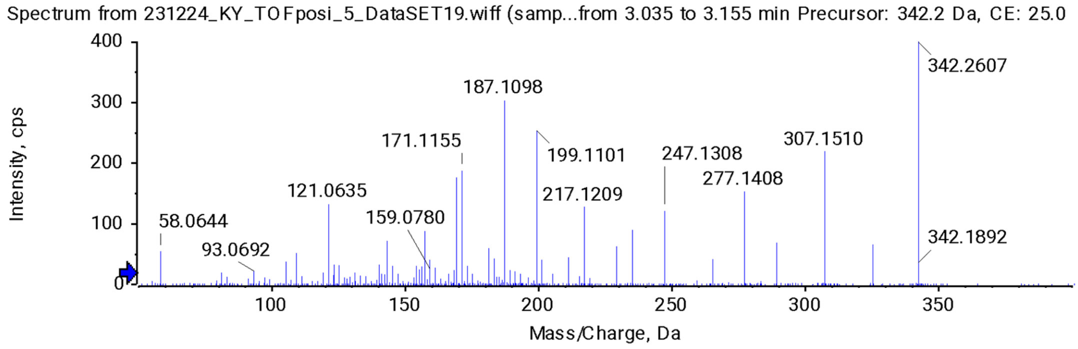

(N) isotrichodiol [M+NH4]$^+$ *m/z*: 270.206, RT: 4.11 min

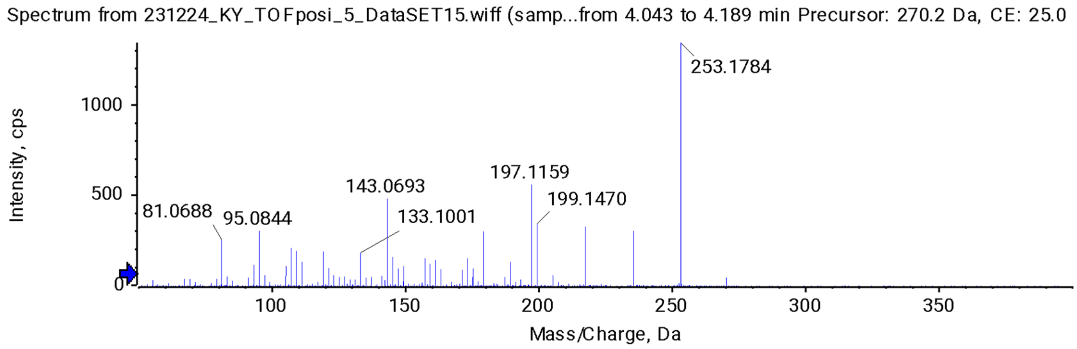

(O) 3,15-dideacetylcalonectrin (3,15-dideCAL) [M+NH4]$^+$ *m/z*: 284.186, RT: 2.77 min

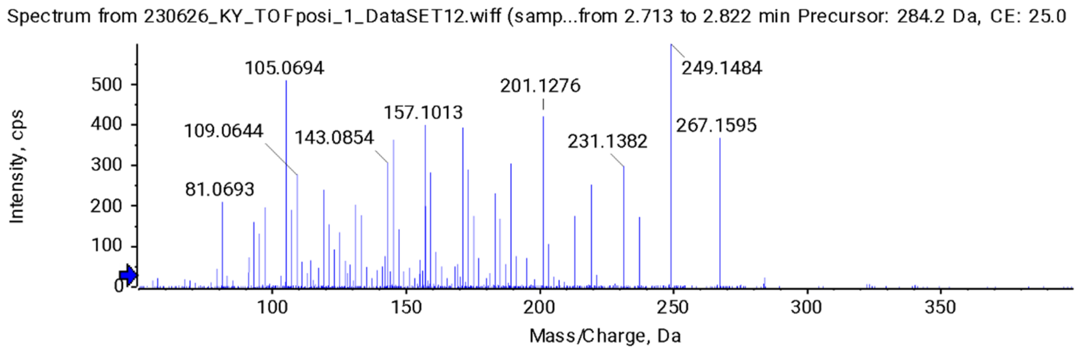

(P) DON [M+NH4]$^+$ *m/z*: 314.160, RT: 2.29 min

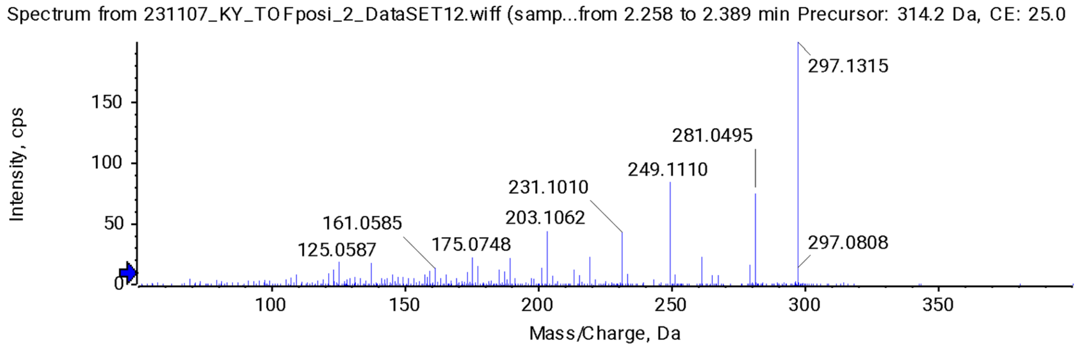

(Q) 3-ADON [M+H]$^+$ *m/z*: 339.144, RT: 3.10 min

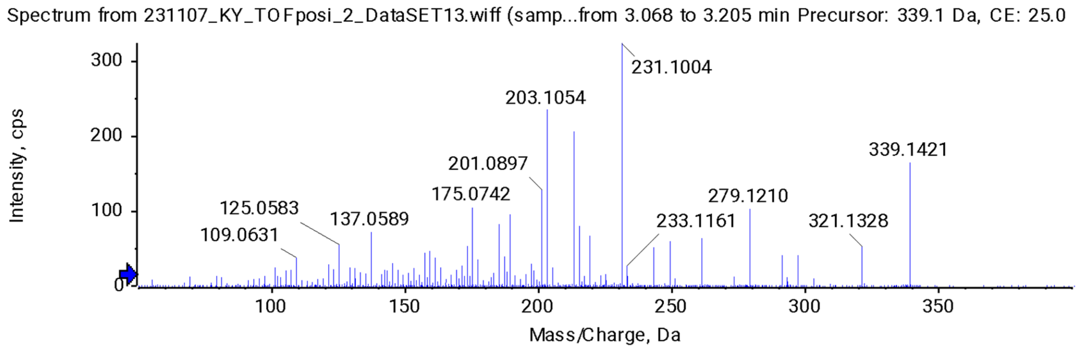

(R) 15-ADON $[M+NH_4]^+$ *m/z*: 356.170, RT: 3.05 min

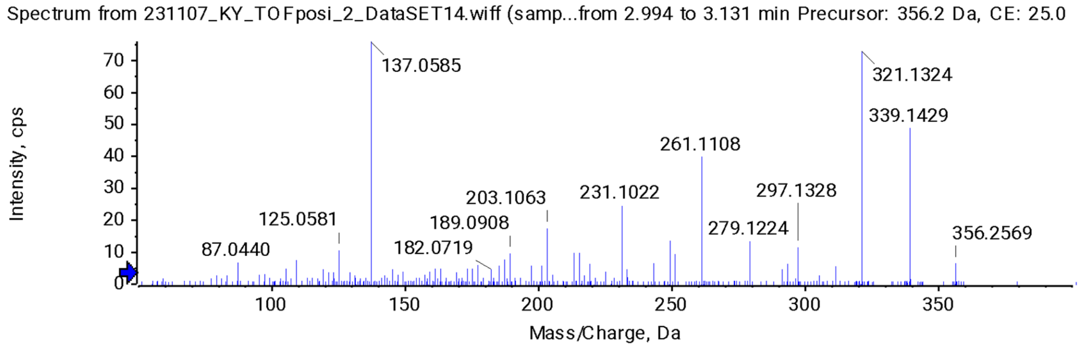

(S) 3,15-diADON $[M+NH_4]^+$ *m/z*: 398.181, RT: 3.84 min

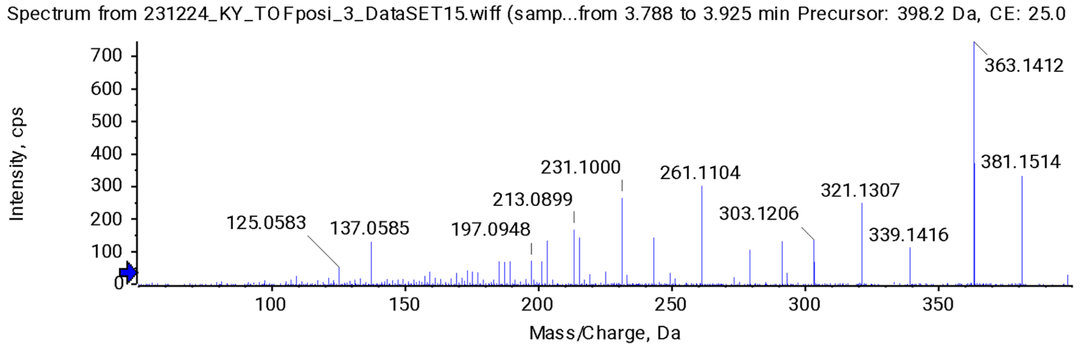

Supplementary Figure S4. MS/MS spectra of 16 tricyclic and 3 bicyclic trichothecenes and shunts. The purified samples were applied to LC-MS, and their MS/MS spectra were obtained in the positive ion mode, using the TOF–MS methods.

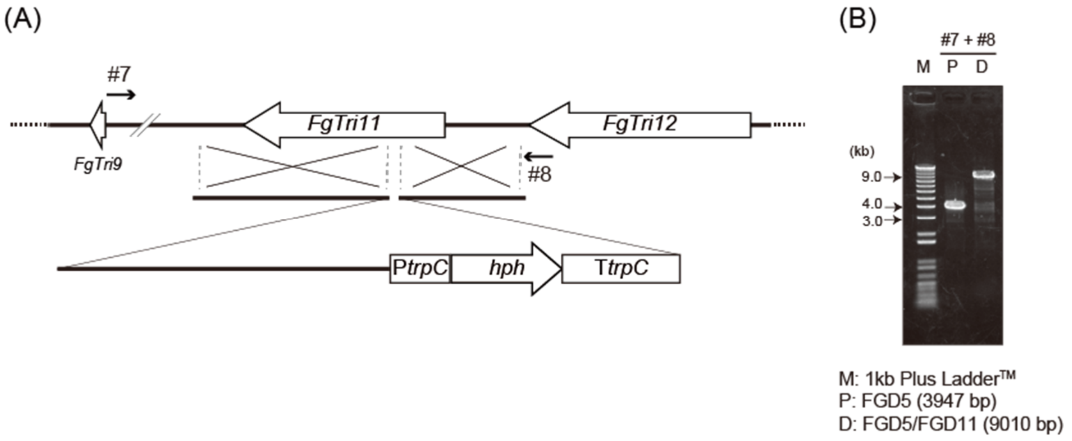

Supplementary Figure S5. Generation of a Δ*Fgtri5*Δ*Fgtri11* (FGD5/11) disruption mutant used in this study. (A) The *FgTri11* gene disruption vector [7] that contains the *hph* (hygromycin B phosphotransferase gene) cassette was transformed into FGD5 mutant strain [8]. After transformation, colonies were selected with 300 µg/ml of hygromycin B. (B) PCR confirmation of genomic DNA isolated from the FGD5 and FGD5/FGD11 mutant strains. Primers #7 and #8 were used to amplify the region encompassing *FgTri11*. An amplicon from the double disruption mutant is 5.0 kb larger than that from its parent FGD5.

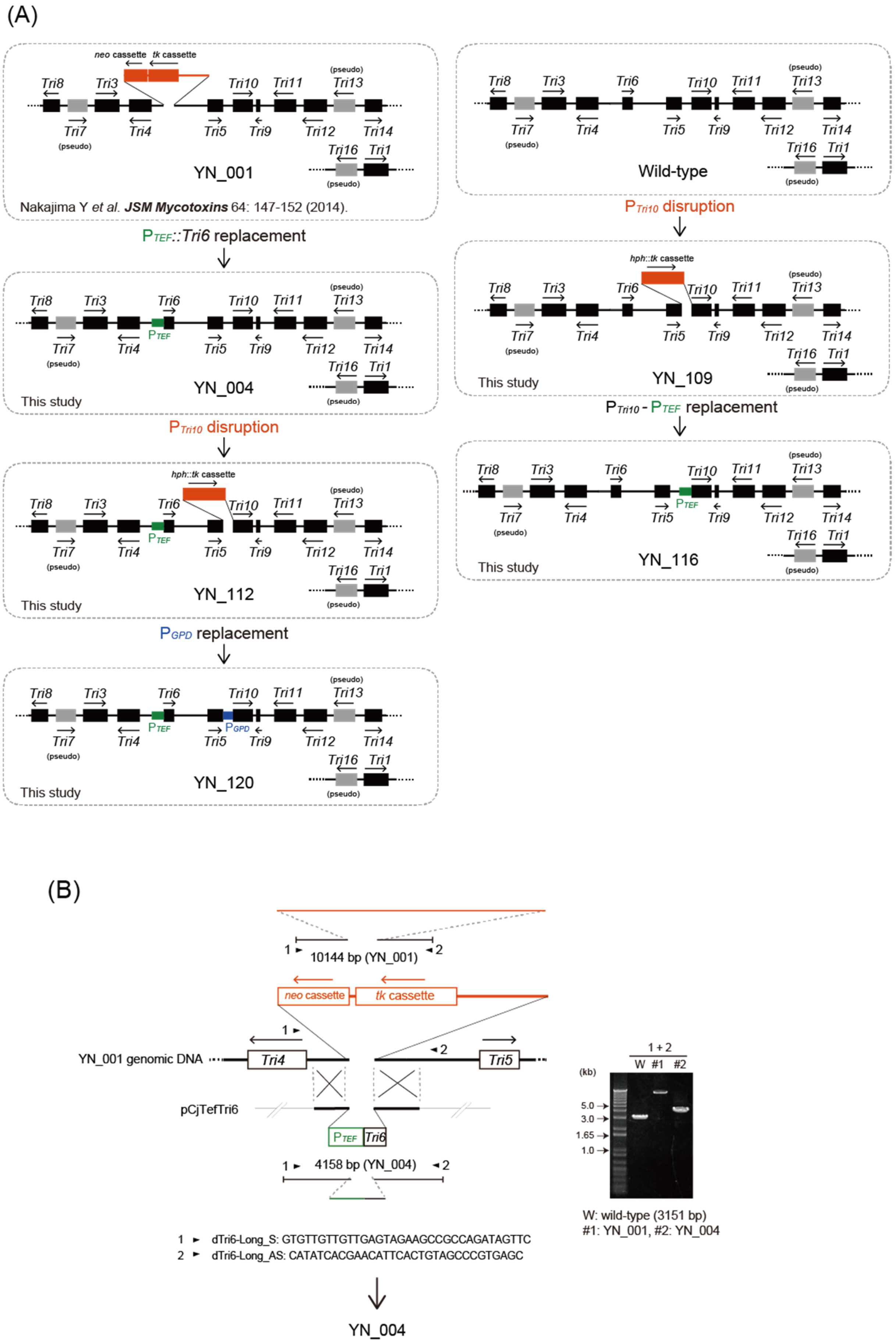
(A)
neo cassette
tk cassette
YN_001
Nakajima Y et al. JSM Mycotoxins 64: 147-152 (2014).
PTEF::Tri6 replacement
YN_004
This study
PTri10 disruption
hph::tk cassette
YN_112
This study
PGPD replacement
YN_120
This study
Wild-type
PTri10 disruption
YN_109
This study
PTri10 - PTEF replacement
YN_116
This study
Tri8 Tri3 Tri6 Tri10 Tri11 Tri13 Tri7 Tri4 Tri5 Tri9 Tri12 Tri14 Tri16 Tri1
(pseudo)
(B)
10144 bp (YN_001)
neo cassette
tk cassette
YN_001 genomic DNA
Tri4
Tri5
pCjTefTri6
PTEF Tri6
4158 bp (YN_004)
1 + 2
W #1 #2
(kb)
5.0
3.0
1.65
1.0
W: wild-type (3151 bp)
#1: YN_001, #2: YN_004
1 ► dTri6-Long_S: GTGTTGTTGTTGAGTAGAAGCCGCCAGATAGTTC
2 ► dTri6-Long_AS: CATATCACGAACATTCACTGTAGCCCGTGAGC
YN_004

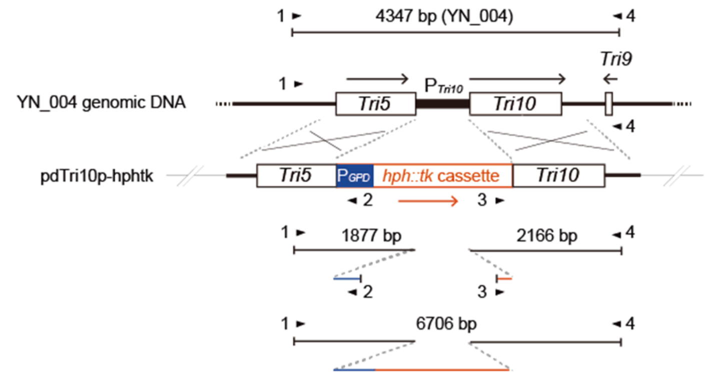

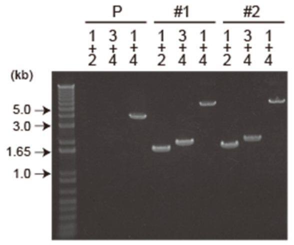

1 ► dTri10p-Long_S: GAGGCTTTCTTCTGTGTTGATAGATGGTCG
2 ► Specific_on_pgpd2: TGATGCAATAGATTCTTGGTCAACGAGGGGC
3 ► Specific_on_HSVtk2: GCACGTCTTTATCCTGGATTACGACCAATCG
4 ► dTri10p-Long_AS: GTTAGCGCTGCTTTATGCGCTACCATATGG

P: YN_004 (parental strain)
#1: YN_112, #2: YN_113

↓

YN_112, YN_113

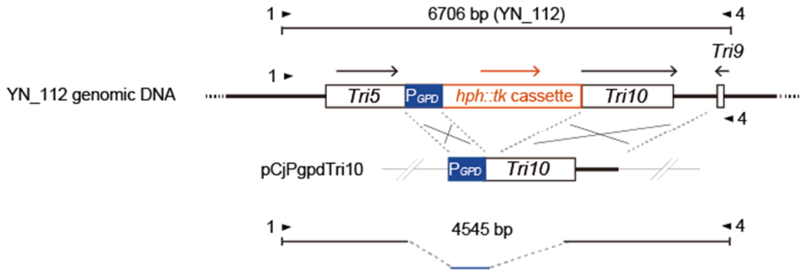

1 ► dTri10p-Long_S: GAGGCTTTCTTCTGTGTTGATAGATGGTCG
4 ► dTri10p-Long_AS: GTTAGCGCTGCTTTATGCGCTACCATATGG

↓

YN_120, YN_121

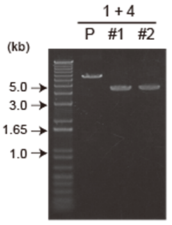

P: YN_112 (parental strain)
#1: YN_120, #2: YN_121

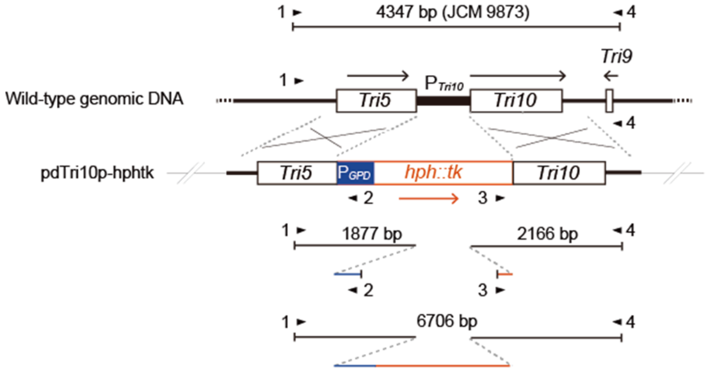

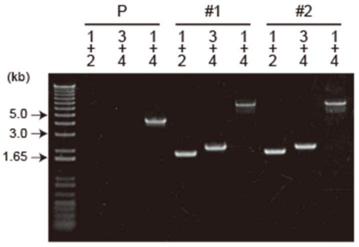

P: JCM 9873 (parental strain)
#1: YN_109, #2: YN_110

1 ► dTri10p-Long_S: GAGGCTTTCTTCTGTGTTGATAGATGGTCG
2 ► Specific_on_pgpd2: TGATGCAATAGATTCTTGGTCAACGAGGGGC
3 ► Specific_on_HSVtk2: GCACGTCTTTATCCTGGATTACGACCAATCG
4 ► dTri10p-Long_AS: GTTAGCGCTGCTTTATGCGCTACCATATGG

↓

YN_109, YN_110

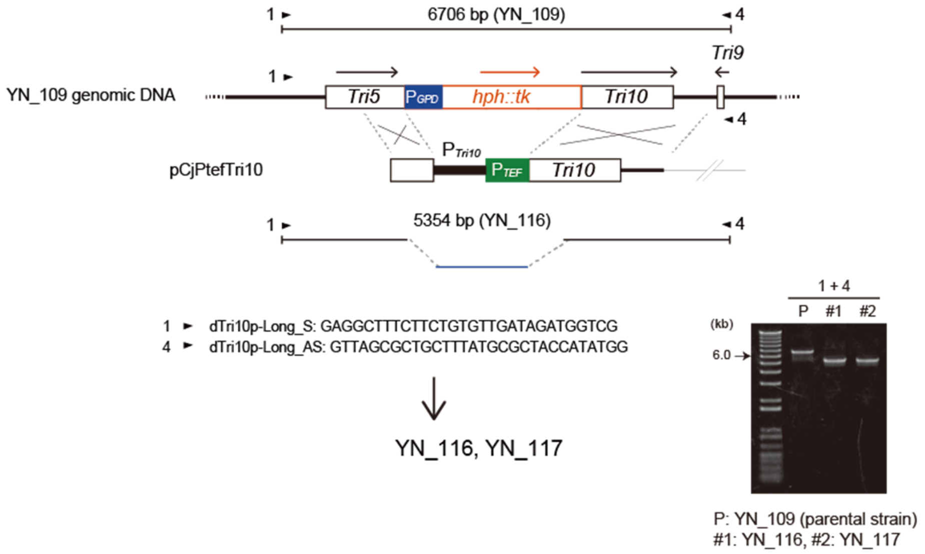
1
6706 bp (YN_109)
4
Tri9
YN_109 genomic DNA
Tri5
PGPD
hph::tk
Tri10
PTri10
pCjPtefTri10
PTEF
Tri10
5354 bp (YN_116)
1 ► dTri10p-Long_S: GAGGCTTTCTTCTGTGTTGATAGATGGTCG
4 ► dTri10p-Long_AS: GTTAGCGCTGCTTTATGCGCTACCATATGG
YN_116, YN_117
1 + 4
P #1 #2
(kb)
6.0
P: YN_109 (parental strain)
#1: YN_116, #2: YN_117

(C)

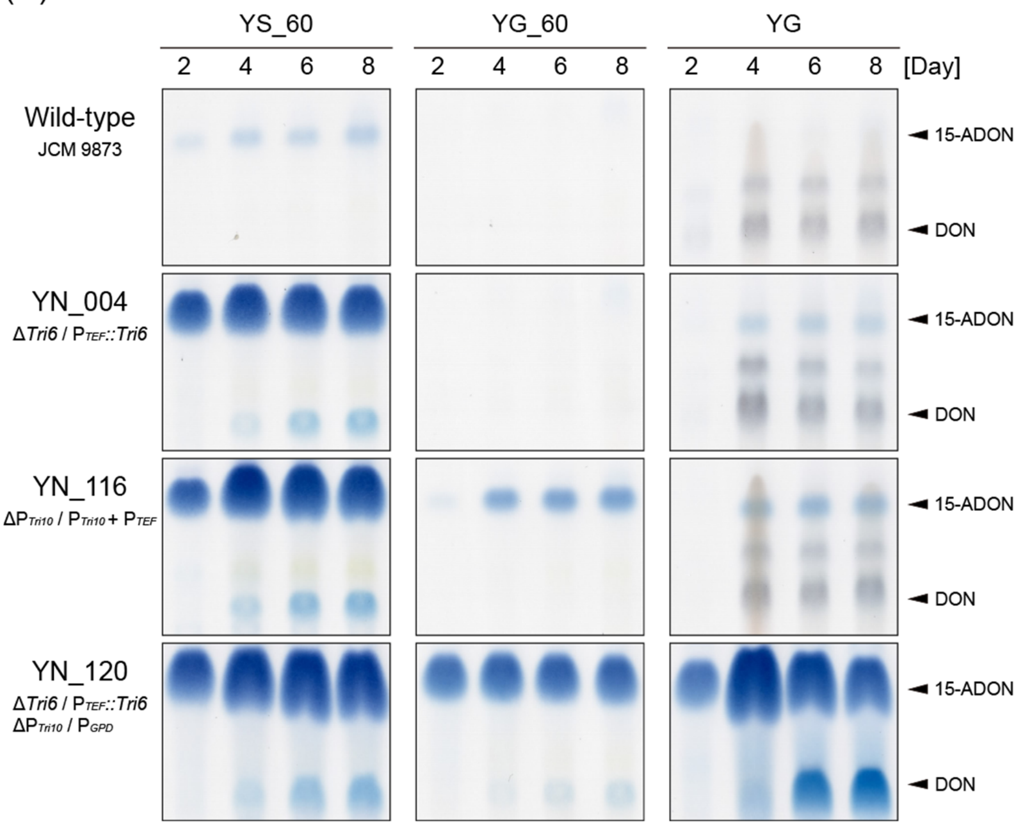
YS_60
YG_60
YG
2 4 6 8
[Day]
Wild-type
JCM 9873
YN_004
ΔTri6 / PTEF::Tri6
YN_116
ΔPTri10 / PTri10 + PTEF
YN_120
ΔTri6 / PTEF::Tri6
ΔPTri10 / PGPD
15-ADON
DON

(D)

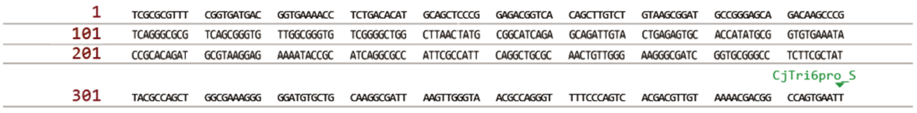
1 TCGCGCGTTT CGGTGATGAC GGTGAAAACC TCTGACACAT GCAGCTCCCG GAGACGGTCA CAGCTTGTCT GTAAGCGGAT GCCGGGAGCA GACAAGCCCG
101 TCAGGGCGCG TCAGCGGGTG TTGGCGGGTG TCGGGGCTGG CTTAACTATG CGGCATCAGA GCAGATTGTA CTGAGAGTGC ACCATATGCG GTGTGAAATA
201 CCGCACAGAT GCGTAAGGAG AAAATACCGC ATCAGGCGCC ATTCGCCATT CAGGCTGCGC AACTGTTGGG AAGGGCGATC GGTGCGGGCC TCTTCGCTAT
CjTri6pro_S
301 TACGCCAGCT GGCGAAAGGG GGATGTGCTG CAAGGCGATT AAGTTGGGTA ACGCCAGGGT TTTCCCAGTC ACGACGTTGT AAAACGACGG CCAGTGAATT

CjTri6pro_S
Tri6_upstream region
401 CGAGCTCGGT ACCCAGGTAC CTTGTCTATC GCGTCTCTAG AGTGCTTTGC ATGCGTTGTG GCCGTAAACG CTCACAACTC TGAAGTTGTC CTCAGTATCG

Tri6_upstream region
501 CCGTGTCAGA TAAGCTTCGC AAGAGTGTCC GGTCGGAAAC TCACCAATCA ACTCAGCAGG ATGAACAAGG GGTCTGAAAG GCCTGGCAGG CCTGACAGGA

Tri6_upstream region
601 GTGATAAAAT GTGAGAAGAG ATATGCCGAT ACAACCGTGT AACTTGTGAA ACGGGGCATG GAATCCCATG GCAAGTTATG GGGTCAGCAG CAACTGAATT

Tri6_upstream region
701 GCCTACGAGT CAAGAAGTGC ATCCTTTCAC CGGCGGCTTA TCCGAAGTTG CTGCCGATCA GATACAGACA TGCATGCAGA GTGGTACGAC TGCGCGGAAG

Tri6_upstream region
801 AATAAGAATC ATCAGTGCGC CGCAATGTTA AAAACTGATG TGCGGAAGCA ACATTAAGCT TTGGAGGCAT GCCAGGGTCT TGTCTCGAAA TATCTTTGTC

Tri6_upstream region
901 TACCGAGACC CATGCATGGT CAAAAGTATG TACATGGATG GTCTTGCACA GAAGACAGCC TCGAGTGTTA TGCAGACTGT CACGGCTGCA GTAAGTTGCC

Tri6_upstream region
1001 ACAGACTCGA ATCGATTATC ATTGACCGTT CGGAAGCGCT CTGTTAGGAA TCTTTCTAGA CCACAACTAC CACTTTGGCA TCTGCATACT AACACTAGTA

Tri6_upstream region
1101 GCCACATAGT AAACCTTCAA CTGCCGCCGC ATCAAACTGT AAACAGGTAC CGGCCGACGC GTCTCGGATA AGAATACCTT TTAAACTGCC GTAGCAAACT

Tri6_upstream region
1201 GTAATTGTCG GTACTTCTCG GACAATATTT TCATGGCTTT CAGAAGCTTT CACTTTTAAT AAAACTTGAT CTGAATAAGA AACTTTATCA ATCGTATCCC

TEF promoter
CTefjTri6_S
Tri6_upstream region
1301 ATCCCATCAA GGCTCAAGCC ATCTTTTATT TTTTATTTTT TGCATCGCCA ACCAATATAT TGAACATCTA TTTTGACTAC CCTCGAATAG CGACTTAGAG
jT6Cpro_AS

CTefjTri6_S
TEF promoter
1401 TGCACGCTCA AAAGATACCC CTAACTTCTG ATTTGATAGC ATTTGTATAT GGCATATAGT ACGCCTGAAG CATAAAAAAA AAAACAGAAT TATTATAGTT

TEF promoter
1501 TATTAATACG AGACAGCAGA ATCACCGCCC AAGTTAAGCC TTTGTGCTGA TCATGCTCTC GAACGGGCCA AGTTCGGGAA AAGCAAAGGA GCGTTTAGTG

TEF promoter
1601 AGGGGCAATT TGACTCACCT CCCAGGCAAC AGATGAGGGG GGCAAAAAGA AAGAAATTTT CGTGAGTCAA TATGGATTCC GAGCATCATT TTCTTGCGGT

TEF promoter
1701 CTATCTTGCT ACGTATGTTG ATCTTGACGC TGTGGATCAA GCAACGCCAC TCGCTCGCTC CATCGCAGGC TGGTCGCAGA CAAATTAAAA GGCGGCAAAC

TEF promoter
1801 TCGTACAGCC GCGGGGTTGT CCGCTGCAAA GTACAGAGTG ATAAAAGCCG CCATGCGACC ATCAACGCGT TGATGCCCAG CTTTTTCGAT CCGAGAATCC

TEF promoter
1901 ACCGTAGAGG CGATAGCAAG TAAAGAAAAG CTAAACAAAA AAAAATTTCT GCCCCTAAGC CATGAAAACG AGATGGGGTG GAGCAGAACC AAGGAAAGAG

TEF promoter
2001 TCGCGCTGGG CTGCCGTTCC GGAAGGTGTT GTAAAGGCTC GACGCCCAAG GTGGGAGTCT AGGAGAAGAA TTTGCATCGG GAGTGGGGCG GGTTACCCCT

TEF promoter
2101 CCATATCCAA TGACAGATAT CTACCAGCCA AGGGTTTGAG CCCGCCCGCT TAGTCGTCGT CCTCGCTTGC CCCTCCATAA AAGGATTTCC CCTCCCCCTC

TEF promoter
2201 CCACAAAATT TTCTTTCCCT TCCTCTCCTT GTCCGCTTCA GTACGTATAT CTTCCCTTCC CTCGCTTCTC TCCTCCATCC TTCTTTCATC CATCTCCTGC

Tri6
jT6C_S
TEF promoter
+1 M I
2301 TAACTTCTCT GCTCAGCACC TCTACGCATT ACTAGCCGTA GTATCTGAGC ACTTCTCCCT TTTATATTCC ACAAAACATA ACACAACCTT CACCATGATT
CTefjTri6_AS

jT6C_S
Tri6
+1 Y M E A E S H Y E S W S A L P L F D R V A S P D P A K D F V P D L N
2401 TACATGGAGG CCGAATCTCA CTACGAATCT TGGAGCGCCT TGCCCCTCTT TGATCGAGTT GCGTCTCCCG ATCCTGCCAA GGACTTTGTC CCAGATCTAA
CTefjTri6_AS

Tri6
+1 · N D Y E S P T F E I D L L S E T Y D F D N F P T Y S L P T V D S T K
2501 ACGACTATGA ATCACCAACA TTCGAAATAG ATCTTCTCTC AGAAACTTAT GACTTTGACA ACTTCCCCAC ATACTCTCTA CCAACGGTGG ATTCAACCAA

Tri6
+1 · K T L Y S E E P L V C F D F D F A N P A I E N Y I T T S S G L L D A
2601 GACTTTGTAC TCCGAAGAAC CACTTGTTTG CTTCGACTTT GACTTCGCGA ACCCGGCTAT CGAAAATTAT ATAACCACAT CGTCGGGACT GTTGGACGCA

Tri6
+1 V P S Q L I A L P T F T R P S K C P F P S C K S A T V F E S G R D F
2701 GTGCCAAGCC AGCTTATCGC CCTTCCCACC TTCACACGGC CAAGCAAATG CCCATTCCCT AGTTGCAAGT CGGCCACAGT CTTTGAAAGC GGACGGGACT

Tri6
+1 · F R R H Y R Q H F K R F F C R Y S E C P Q S A Q D L Q E V G T K G F
2801 TTAGGCGGCA TTACCGGCAA CACTTCAAGC GCTTTTTCTG TCGCTACTCA GAATGCCCTC AGTCAGCTCA AGACCTGCAA GAAGTCGGCA CCAAAGGCTT

Tri6
+1 · F A T R K D R A R H E S K H K P T V R C P W Q D K E G Q Q C L R V F
2901 TGCGACTCGC AAGGACCGTG CTCGGCATGA GTCTAAGCAC AAACCAACAG TGCGGTGCCC TTGGCAAGAC AAGGAAGGAC AACAATGTCT GAGGGTCTTT

Tri6
Tri6 downstream region
+1 S R V D N M R D H Y R R I H K C *
3001 AGCAGGGTGG ATAACATGCG AGATCACTAT AGGCGGATAC ATAAGTGTTG ACGAGGGATC GGTGTGCAAA ACTGCAGATA GTTACTCATA AAAGGCAACA

Tri6 downstream region
3101 TTTGCGAAAA TCAATAACAA TATCTCCATA AATATCCCAT TAGACTTTTT GAGTTTCATA AATGATGACG AGTTTGGCGC CGATCGATGC TGTTTCCAGC

Tri6 downstream region
3201 ACCAATTCAT AATATACTCC ATAGTAGCCG AGACCCTGCA GCTCATTGGT ACCAGCCCTG TTTTTATTGC ACAATTAATG ACACTCTATT CTGCACGCGA

Tri6 downstream region
3301 CTCAACGGCT TTCAACACTT ACATATTGCC TCTACGCGTG GCTGTGTCTA AGCGAGATCA GCGCGGTCAT TGTTATTGCT GCGTTGCTTT GGCCCCGTAC

Tri6 downstream region
3401 GTTCCACAAC AATCTGGCCA CTGCAAGGTA TTTGCGGCCG CTGGTTAAAT TCTCTTAGTA GTCTGCCTCA TTTGGCAGGT GCCAATAATA TATTTTACCC

Tri6 downstream region
3501 GCGTTTGGCT GGCCATGCTA CCCAAAAGGC TTCATACATA TCTGGCCCAT ATCGTTTACT CGGTTCCACG TTTACGAGCA TACGCCTCTC GTATCAATGT

Tri6 downstream region
3601 CGCAAACAAA CATACGTCAT TGGTTACTAT GCCGGGTTTC CGCGAACCAT TGCTGTGCCG CAAAAGGATG CATCGGCACT CTAAAGATTG CGATCCTGTG

Tri6 downstream region
3701 TTAGAGGGTG ACTTGAGTCA AGAAGTAACG TAACAAGTGA GATAATGAAA TTAATTACCT GAGGGCAATT TAAGGTTTCA ACCTCCGAGG AAAGCCATTC

Tri6 downstream region
3801 GTCGTGGGAT ATAATCTGCA ATAGGGTAGG CTTTGCTGTT TTTTTCGGAG CATTTGTGAC TGTTTTGGAT TGTGTCTTTG GGGTCCTTTT GCTTTCAAGG

Tri6 downstream region
3901 CTGGTGAGTT TGCAAGGAAC GAGTCAGTCA CGGCAGGCAC TGAGTCAAAC ACTGTTTCGT AATAATCATG ATTTGTTGTT GCAAAATTCC AACGAAGCTT

Tri6 downstream region
4001 TGCGCGGTCA GTCAAATTCG CACACGTCGA TACTTTTTGC TGGCATTGGC CCAGTATTCA TCCATGAGTG GCTGAACCGT AGTTGATTCT CAAGTCCAAC

Tri6 downstream region
4101 CCTAATAGTG CCCGGCGGAA TGAGACGTTT TCGGGTGTCT GTAGCCGAGA TGTGGATAGT AACGGTAACC CTAGTCAAAT GAGACGTGGG CAGGGTTCAT

Tri6 downstream region
4201 GGTTGTTGAA CCTTGTTCAT CAGAATGTTG ATGCCGTTGA CCTACGGAAT ACCATCTTTC ACATGTATTT GTTCCCAACC CACGTGGCTA TACCAACATC

Tri6 downstream region BamHI
4301 CGCTGTATAT TCATTGGTTG GCTTTATATA TTGATACAGG TAATTTCAAG ATCGCGAGCG GGAATTTCCT TGTCGGATCA GAGAGATGCT CTGGGGATCC
jT6Cter_AS

4401 TCTAGAGTCG ACCTGCAGGC ATGCAAGCTT GGCGTAATCA TGGTCATAGC TGTTTCCTGT GTGAAATTGT TATCCGCTCA CAATTCCACA CAACATACGA
jT6Cter_AS

4501 GCCGGAAGCA TAAAGTGTAA AGCCTGGGGT GCCTAATGAG TGAGCTAACT CACATTAATT GCGTTGCGCT CACTGCCCGC TTTCCAGTCG GGAAACCTGT
4601 CGTGCCAGCT GCATTAATGA ATCGGCCAAC GCGCGGGGAG AGGCGGTTTG CGTATTGGGC GCTCTTCCGC TTCCTCGCTC ACTGACTCGC TGCGCTCGGT
4701 CGTTCGGCTG CGGCGAGCGG TATCAGCTCA CTCAAAGGCG GTAATACGGT TATCCACAGA ATCAGGGGAT AACGCAGGAA AGAACATGTG AGCAAAAGGC
4801 CAGCAAAAGG CCAGGAACCG TAAAAAGGCC GCGTTGCTGG CGTTTTTCCA TAGGCTCCGC CCCCCTGACG AGCATCACAA AAATCGACGC TCAAGTCAGA
4901 GGTGGCGAAA CCCGACAGGA CTATAAAGAT ACCAGGCGTT TCCCCCTGGA AGCTCCCTCG TGCGCTCTCC TGTTCCGACC CTGCCGCTTA CCGGATACCT
5001 GTCCGCCTTT CTCCCTTCGG GAAGCGTGGC GCTTTCTCAA TGCTCACGCT GTAGGTATCT CAGTTCGGTG TAGGTCGTTC GCTCCAAGCT GGGCTGTGTG
5101 CACGAACCCC CCGTTCAGCC CGACCGCTGC GCCTTATCCG GTAACTATCG TCTTGAGTCC AACCCGGTAA GACACGACTT ATCGCCACTG GCAGCAGCCA
5201 CTGGTAACAG GATTAGCAGA GCGAGGTATG TAGGCGGTGC TACAGAGTTC TTGAAGTGGT GGCCTAACTA CGGCTACACT AGAAGGACAG TATTTGGTAT
5301 CTGCGCTCTG CTGAAGCCAG TTACCTTCGG AAAAAGAGTT GGTAGCTCTT GATCCGGCAA ACAAACCACC GCTGGTAGCG GTGGTTTTTT TGTTTGCAAG
5401 CAGCAGATTA CGCGCAGAAA AAAAGGATCT CAAGAAGATC CTTTGATCTT TTCTACGGGG TCTGACGCTC AGTGGAACGA AAACTCACGT TAAGGGATTT
5501 TGGTCATGAG ATTATCAAAA AGGATCTTCA CCTAGATCCT TTTAAATTAA AAATGAAGTT TTAAATCAAT CTAAAGTATA TATGAGTAAA CTTGGTCTGA
5601 CAGTTACCAA TGCTTAATCA GTGAGGCACC TATCTCAGCG ATCTGTCTAT TTCGTTCATC CATAGTTGCC TGACTCCCCG TCGTGTAGAT AACTACGATA
bla

5701 CGGGAGGGCT TACCATCTGG CCCCAGTGCT GCAATGATAC CGCGAGACCC ACGCTCACCG GCTCCAGATT TATCAGCAAT AAACCAGCCA GCCGGAAGGG
bla

5801 CCGAGCGCAG AAGTGGTCCT GCAACTTTAT CCGCCTCCAT CCAGTCTATT AATTGTTGCC GGGAAGCTAG AGTAAGTAGT TCGCCAGTTA ATAGTTTGCG
bla

5901 CAACGTTGTT GCCATTGCTA CAGGCATCGT GGTGTCACGC TCGTCGTTTG GTATGGCTTC ATTCAGCTCC GGTTCCCAAC GATCAAGGCG AGTTACATGA
bla

6001 TCCCCCATGT TGTGCAAAAA AGCGGTTAGC TCCTTCGGTC CTCCGATCGT TGTCAGAAGT AAGTTGGCCG CAGTGTTATC ACTCATGGTT ATGGCAGCAC
bla

6101 TGCATAATTC TCTTACTGTC ATGCCATCCG TAAGATGCTT TTCTGTGACT GGTGAGTACT CAACCAAGTC ATTCTGAGAA TAGTGTATGC GGCGACCGAG
bla

6201 TTGCTCTTGC CCGGCGTCAA TACGGGATAA TACCGCGCCA CATAGCAGAA CTTTAAAAGT GCTCATCATT GGAAAACGTT CTTCGGGGCG AAAACTCTCA
bla

6301 AGGATCTTAC CGCTGTTGAG ATCCAGTTCG ATGTAACCCA CTCGTGCACC CAACTGATCT TCAGCATCTT TTACTTTCAC CAGCGTTTCT GGGTGAGCAA
bla

6401 AAACAGGAAG GCAAAATGCC GCAAAAAAGG GAATAAGGGC GACACGGAAA TGTTGAATAC TCATACTCTT CCTTTTTCAA TATTATTGAA GCATTTATCA
bla

6501 GGGTTATTGT CTCATGAGCG GATACATATT TGAATGTATT TAGAAAAATA AACAAATAGG GGTTCCGCGC ACATTTCCCC GAAAAGTGCC ACCTGACGTC
6601 TAAGAAACCA TTATTATCAT GACATTAACC TATAAAAATA GGCGTATCAC GAGGCCCTTT CGTC

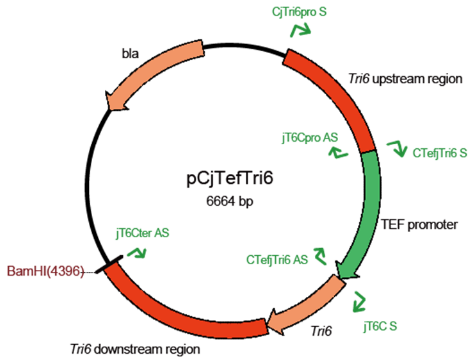

Upstream HR
dTri10P_U_S
1 GGGTAACGCC AGGGTTTTCC CAGTCACGAC GTTGTAAAAC GACGGCCAGT GCCAAGCTTG CATGCCTGCA GGTCGACTCT AGAGTTTGTT CCAAGAGCCG
Vb_AS

Upstream HR
dTri10P_U_S
101 GATGTTTTCT GATATCTGTA GCCTTGCAGG GAATGAGAGA GCACGTCCAT ACGTCAAGGT CTCTCTTCAC GACTGTCTGG TTGGGGACGC TATTCGCATT

Upstream HR
201 GACTTTGGAT CAGTCTTTAG GCCTAACAAT ACAATCTTGA CTAACAACTG CATACGGGTC AAGATATTTG CCATCTAATG ATATATAGTT AATAGCAACA

Tri5
Upstream HR
301 GAACTTTGTA ATTAGAGAAT GACAACGACT TGACTTGTTA ATTTATTGAA TAACTGTTAC CAGTACAACC TTGCCATCAT GGAAAACTTT CCCACCGAGT

Tri5
Upstream HR
401 ATTTTCTCAA CACTAGCGTG CGCCTTCTCG AGTATATTCG ATACCGAGAC AGCAATTACA CCCGAGAGGA GCGCATCGAG AATTTGCACT ATGCTTACAA

Tri5
Upstream HR
501 CAAGGCTGCC CACCACTTTG CTCAGCCTCG CCAACAGCAG ATGCTCAAGG TAGACCCTAA GCGACTACAG GCTTCCCTCC AAACAATCGT TGGCATGGTT

Tri5
Upstream HR
601 GTATACAGCT GGGCAAAGGT GTCCAAAGAG TGCATGGCGG ATCTATCTAT TCACTACACC TATACTCTCG TTTTGGATGA CAGCAGCGAT GATCCCCATC

Tri5
Upstream HR
701 CTGCCATGTT GAACTATTTT GACGACCTTC AAGCCGGACG AGAGCAGGCC CATCCATGGT GGGCACTTGT CAACGAGCAC TTTCCCAACG TCCTTCGCCA

Tri5
Upstream HR
801 TTTTGGACCT TTCTGCTCAT TGAACCTTAT CCGTAGCACT ATGGACTGTA AGATACCGAT TCTATTATTC ACCCGTATGA AGAAGCTAAC GGCGGTGGAA

Tri5
Upstream HR
901 CAACAGTTTT TGAGGGATGT TGGATTGAGC AGTACAACTT TGGAGGATTC CCAGGATCTG ATGACTACCC TCAATTCCTT CGTCGTATGA ATGGTTTGGG

Tri5
Upstream HR
1001 TCATTGTGTT GGGGCTTCTC TATGGCCCAA GGACCTGTTT GATGAGCGGA AGCATTTCCT TGAAATCACG TCAGCCGTTG CTCAGATGGA GAACTGGATG

Tri5
Upstream HR
1101 GTTTGGGTCA ATGATCTCAT GTCATTCTAC AAGGAATTCG ACGATGAGCG TGACCAAATC AGTCTGGTCA AGAACTTTGT CACCTGCCAT GAGATCACTC

Tri5
Upstream HR
1201 TGGATGAAGC TTTGGAGAAG CTCACCCAGG AAACCCTACA CTCGTCTAAG CAGATGGTTG CTGTCTTCTC GGACAAGGAC CCTCAGGTGA TGGACACGAT

Tri5
Upstream HR
1301 TGAGTGTTTC ATGCATGGCT ACGTCACGTG GCACTTGTGC GACGCTCGAT ACCGCCTCCA TGAGATTTAT GAAAAGGTCA AGGATCAGGA TACAGAGGAC

Tri5
Upstream HR
1401 GCCAAGAAGT TCTGCAAGTT CTTTGAGCAG GCGGCCAATG TCGGCGCCGT TGCACCCTCG GAGTGGGCTT ATCCACAAGT TGCACAACTG GCAAACGTTC

hphtkC2_S
Tri5
Upstream HR
GPD promoter
1501 GGGCCAAGGA CGATGTGAAG GAGGCTCAGA AGCCCATCCT AAGTTCAATT GAGCTAGTGG AGTAACCCCC GGTGACTCTT TCTGGCATGC GGAGAGACGG
dTri10P-U_AS

GPD promoter
1601 ACGGACGCAG AGAGAAGGGC TGAGTAATAA GCGCCACTGC GCCAGACAGC TCTGGCGGCT CTGAGGTGCA GTGGATGATT ATTAATCCGG GACCGGCCGC

GPD promoter
1701 CCCTCCGCCC CGAAGTGGAA AGGCTGGTGT GCCCCTCGTT GACCAAGAAT CTATTGCATC ATCGGAGAAT ATGGAGCTTC ATCGAATCAC CGGCAGTAAG

GPD promoter
1801 CGAAGGAGAA TGTGAAGCCA GGGGTGTATA GCCGTCGGCG AAATAGCATG CCATTAACCT AGGTACAGAA GTCCAATTGC TTCCGATCTG GTAAAAGATT

GPD promoter
1901 CACGAGATAG TACCTTCTCC GAAGTAGGTA GAGCGAGTAC CCGGCGCGTA AGCTCCCTAA TTGGCCCATC CGGCATCTGT AGGGCGTCCA AATATCGTGC

GPD promoter
2001 CTCTCCTGCT TTGCCCGGTG TATGAAACCG GAAAGGCCGC TCAGGAGCTG GCCAGCGGCG CAGACCGGGA ACACAAGCTG GCAGTCGACC CATCCGGTGC

GPD promoter
2101 TCTGCACTCG ACCTGCTGAG GTCCCTCAGT CCCTGGTAGG CAGCTTTGCC CCGTCTGTCC GCCCGGTGTG TCGGCGGGGT TGACAAGGTC GTTGCGTCAG

GPD promoter
2201 TCCAACATTT GTTGCCATAT TTTCCTGCTC TCCCCACCAG CTGCTCTTTT CTTTTCTCTT TCTTTTCCCA TCTTCAGTAT ATTCATCTTC CCATCCAAGA

GPD promoter
2301 ACCTTTATTT CCCCTAAGTA AGTACTTTGC TACATCCATA CTCCATCCTT CCCATCCCTT ATTCCTTTGA ACCTTTCAGT TCGAGCTTTC CCACTTCATC

GPD promoter
hph
2401 GCAGCTTGAC TAACAGCTAC CCCGCTTGAG ATCGATATGA AAAAGCCTGA ACTCACCGCG ACGTCTGTCG AGAAGTTTCT GATCGAAAAG TTCGACAGCG

hph
2501 TCTCCGACCT GATGCAGCTC TCGGAGGGCG AAGAATCTCG TGCTTTCAGC TTCGATGTAG GAGGGCGTGG ATATGTCCTG CGGGTAAATA GCTGCGCCGA

hph
2601 TGGTTTCTAC AAAGATCGTT ATGTTTATCG GCACTTTGCA TCGGCCGCGC TCCCGATTCC GGAAGTGCTT GACATTGGGG AGTTCAGCGA GAGCCTGACC

hph
2701 TATTGCATCT CCCGCCGTGC ACAGGGTGTC ACGTTGCAAG ACCTGCCTGA AACCGAACTG CCCGCTGTTC TCCAGCCGGT CGCGGAGGCC ATGGATGCGA

hph
2801 TCGCTGCGGC CGATCTTAGC CAGACGAGCG GGTTCGGCCC ATTCGGACCG CAAGGAATCG GTCAATACAC TACATGGCGT GATTTCATAT GCGCGATTGC

hph
2901 TGATCCCCAT GTGTATCACT GGCAAACTGT GATGGACGAC ACCGTCAGTG CGTCCGTCGC GCAGGCTCTC GATGAGCTGA TGCTTTGGGC CGAGGACTGC

hph
3001 CCCGAAGTCC GGCACCTCGT GCATGCGGAT TTCGGCTCCA ACAATGTCCT GACGGACAAT GGCCGCATAA CAGCGGTCAT TGACTGGAGC GAGGCGATGT

hph
3101 TCGGGGATTC CCAATACGAG GTCGCCAACA TCCTCTTCTG GAGGCCGTGG TTGGCTTGTA TGGAGCAGCA GACGCGCTAC TTCGAGCGGA GGCATCCGGA

hph
3201 GCTTGCAGGA TCGCCGCGCC TCCGGGCGTA TATGCTCCGC ATTGGTCTTG ACCAACTCTA TCAGAGCTTG GTTGACGGCA ATTTCGATGA TGCAGCTTGG

hph
3301 GCGCAGGGTC GATGCGACGC AATCGTCCGA TCCGGAGCCG GGACTGTCGG GCGTACACAA ATCGCCCGCA GAAGCGCGGC CGTCTGGACC GATGGCTGTG

HSVtk
hph
3401 TAGAAGTACT CGCCGATAGT GGAAACCGAC GCCCCAGCAC TCGTCCGAGG GCAAAGGAAA TGGCTTCGTA CCCCGGCCAT CAACACGCGT CTGCGTTCGA

HSVtk
3501 CCAGGCTGCG CGTTCTCGCG GCCATAGCAA CCGACGTACG GCGTTGCGCC CTCGCCGGCA GCAAGAAGCC ACGGAAGTCC GCCCGGAGCA GAAAATGCCC

HSVtk
3601 ACGCTACTGC GGGTTTATAT AGACGGTCCC CACGGGATGG GGAAAACCAC CACCACGCAA CTGCTGGTGG CCCTGGGTTC GCGCGACGAT ATCGTCTACG

HSVtk
3701 TACCCGAGCC GATGACTTAC TGGCGGGTGC TGGGGGCTTC CGAGACAATC GCGAACATCT ACACCACACA ACACCGCCTC GACCAGGGTG AGATATCGGC

HSVtk
3801 CGGGGACGCG GCGGTGGTAA TGACAAGCGC CCAGATAACA ATGGGCATGC CTTATGCCGT GACCGACGCC GTTCTGGCTC CTCATATCGG GGGGGAGGCT

HSVtk

3901 GGGAGCTCAC ATGCCCCGCC CCCGGCCCTC ACCCTCATCT TCGACCGCCA TCCCATCGCC GCCCTCCTGT GCTACCCGGC CGCGCGGTAC CTTATGGGCA

HSVtk

4001 GCATGACCCC CCAGGCCGTG CTGGCGTTCG TGGCCCTCAT CCCGCCGACC TTGCCCGGCA CCAACATCGT GCTTGGGGCC CTTCCGGAGG ACAGACACAT

HSVtk

4101 CGACCGCCTG GCCAAACGCC AGCGCCCCGG CGAGCGGCTG GACCTGGCTA TGCTGGCTGC GATTCGCCGC GTTTACGGGC TACTTGCCAA TACGGTGCGG

HSVtk

4201 TATCTGCAGT GCGGCGGGTC GTGGCGGGAG GACTGGGGAC AGCTTTCGGG GACGGCCGTG CCGCCCCAGG GTGCCGAGCC CCAGAGCAAC GCGGGCCCAC

HSVtk

4301 GACCCCATAT CGGGGACACG TTATTTACCC TGTTTCGGGC CCCCGAGTTG CTGGCCCCCA ACGGCGACCT GTATAACGTG TTTGCCTGGG CCTTGGACGT

HSVtk

4401 CTTGGCCAAA CGCCTCCGTT CCATGCACGT CTTTATCCTG GATTACGACC AATCGCCCGC CGGCTGCCGG GACGCCCTGC TGCAACTTAC CTCCGGGATG

dTri10P-D_S

Downstream HR

HSVtk

Tri10

4501 GTCCAGACCC ACGTCACCAC CCCCGGCTCC ATACCGACGA TATGCGACCT GGCGCGCACG TTTGCCCGGG AGATGGGGGA GGCTAACTGA CATGGATTTC

hphtkC2_AS

Downstream HR

Tri10

dTri10P-D_S

4601 CCAAAGCCTA GACAAGTCCG AGAGACGAGC CTGTTGATGT ACTACCTAGA CGTCGTGTTT CCTCTGCAAT GCATCAACCC AAACAACAAT TGTCTGGGAA

Tri10

Downstream HR

4701 AGAGAGAGTG GCTGTTGACT ATACTGACCT CTGCGCGGCC TACGTACTAT GCCACATTGT GCATGTCGCT CCTCTATAAA GAATCGCTTT CAAGCCCTTG

Tri10

Downstream HR

4801 CAGATCTGAA CAGGCGATGG TATGGAAGAG AGAGAAGACA TACTACTACA TTCTTGCACT CCAGGAGTCT CAGAAGCTGC TGGGTGGGCT CGACAAGACA

Tri10

Downstream HR

4901 TTTGGCATCA CAAGGCTGAA AGGTACCGTC GTTGCCCTTG CTTGCATGCT ACAGCTTATC AGTTTTGAGG TAAGACGAAT CCACCATTGT TTCGATGCTC

Tri10

Downstream HR

5001 GATGTCGATG CTCGATATCC GATCTACGAT TATCGTTGGT CACTAACAAA TTAAAATAGT CTTCGCACCT AAGCAGGGGA GATTGGCGCG TTCACCTCCA

Tri10

Downstream HR

5101 TGCGGCCAAC ATACTCATTC CTGTCTTGGT TGAGGGATGG TCCACAGCTT TGCAATCAGG CCCCCCAGCC ACCTCCATAT GGTGCGAGCT GGATGAATCA

Tri10

Downstream HR

5201 CACTTCGGCT CGACTGAAGA TCAAACCTCT TTGAGCTTCG AATACGTCGG AGCTTTGAGA TTCCTGTCAA ACTCACTCGC CGCAGTCGGC ATCCTGTCTT

Tri10

Downstream HR

5301 GCATATCTAT TGGCCCATCA GCACCATTTG AAGATTACGG CCATCTCCTG GACCAGCCAG GCCTTATACA GCTGGACGAG GTGCTGGGGT GCAGGAATTG

Tri10

Downstream HR

5401 GACCATGTTG ACTATTCTCG AAGTGGGTAA GCTGGATCGT TGGAAGCGAC AGGAGCAAGA ACATAATCGC TTGAGCCTAA AGACGCTCGC TAGGCGCGCC

Tri10

Downstream HR

5501 ATGATGATTG AGGATATGTT GTCAGACGAG CTACAAAGGC TACCGACAGA CGAGACGCTT CCAGACCTCA TCACTCAGAT TTACGCCGCC TCTATCATGA

Tri10

Downstream HR

5601 CGTATCTGCA TACAGTAGTT TCCGGACTCA ATCCCAACCT TTCAGAGGTT CAGGATAGTG TGGCCGGGAC GCTTCAATTG TTGGAGAGGC TCCCAAATCT

Tri10

Downstream HR

5701 TGAAGCTGTC ACGAGCGTTA CTTGGCCTCT AGCTGTCACA GGATGCATGG CCTCAGAAAG TCATAAGGAC TTTTTCAGAA ATACTCTGAG GTCGTATGAG

Tri10

Downstream HR

5801 GCGACATTCA GCTCCTTAAA AAAGTATGAC GGAACTCTTC AGGTCTTGGA AGACGCTTGG AAGAGAAGAG AGATAGATAC AGAGTCTCCA ATGAGATGGG

Tri10

Downstream HR

5901 AAGACTTGAC GGATCACCAT GGGCTTCCAG TGCTACTTTG GTAGGGATGG TATCGGCGCA GAGACTACGG ATACCGATAC CCGAAGTCTT TCTTCTGCAT

Downstream HR

6001 CAGCTACCCG ATACATGTAA GTTTGATGCT CACTTACAGA TGATGCATGC AGGGAAGTTT TATTAGATGT TATGGGTTCG ACTCATTTTG GCGTTTAAAC

Downstream HR

6101 AGGGATTGGC ATGATGCATG ATTTAAATGG AATAGAAGAG CGGAAAACGT TTCCCATGTA ACTTGAATGA TGGACTTTCA ATATATATTT CTAGTATACT

Downstream HR

6201 TTTTTAACCG TGAACCGCAT CTTCTCCATC TATCCAACCA ATCGTGCTGA AAGCAGTCTG GAACCATGGT GGGCGAATCA GTGCTGTGTC CCTTGATTGT

Vb_S

Downstream HR

6301 CTCGACATGG CTTGACATTG TTTAGCCCGT ACGACAAACG ATATTGATCA TAGTGAAGGA GTGTTGATTT CAAGCGAGGG GATTTGTCGA AGTTCGGGGT

dTri10P-D_AS

Vb_S

6401 ACCGAGCTCG AATTCGTAAT CATGGTCATA GCTGTTTCCT GTGTGAAATT GTTATCCGCT CACAATTCCA CACAACATAC GAGCCGGAAG CATAAAGTGT

dTri10P-D_AS

6501 AAAGCCTGGG GTGCCTAATG AGTGAGCTAA CTCACATTAA TTGCGTTGCG CTCACTGCCC GCTTTCCAGT CGGGAAACCT GTCGTGCCAG CTGCATTAAT

6601 GAATCGGCCA ACGCGCGGGG AGAGGCGGTT TGCGTATTGG GCGCTCTTCC GCTTCCTCGC TCACTGACTC GCTGCGCTCG GTCGTTCGGC TGCGGCGAGC

6701 GGTATCAGCT CACTCAAAGG CGGTAATACG GTTATCCACA GAATCAGGGG ATAACGCAGG AAAGAACATG TGAGCAAAAG GCCAGCAAAA GGCCAGGAAC

6801 CGTAAAAAGG CCGCGTTGCT GGCGTTTTTC CATAGGCTCC GCCCCCCTGA CGAGCATCAC AAAAATCGAC GCTCAAGTCA GAGGTGGCGA AACCCGACAG

6901 GACTATAAAG ATACCAGGCG TTTCCCCCTG GAAGCTCCCT CGTGCGCTCT CCTGTTCCGA CCCTGCCGCT TACCGGATAC CTGTCCGCCT TTCTCCCTTC

7001 GGGAAGCGTG GCGCTTTCTC ATAGCTCACG CTGTAGGTAT CTCAGTTCGG TGTAGGTCGT TCGCTCCAAG CTGGGCTGTG TGCACGAACC CCCCGTTCAG

7101 CCCGACCGCT GCGCCTTATC CGGTAACTAT CGTCTTGAGT CCAACCCGGT AAGACACGAC TTATCGCCAC TGGCAGCAGC CACTGGTAAC AGGATTAGCA

7201 GAGCGAGGTA TGTAGGCGGT GCTACAGAGT TCTTGAAGTG GTGGCCTAAC TACGGCTACA CTAGAAGGAC AGTATTTGGT ATCTGCGCTC TGCTGAAGCC

7301 AGTTACCTTC GGAAAAAGAG TTGGTAGCTC TTGATCCGGC AAACAAACCA CCGCTGGTAG CGGTGGTTTT TTTGTTTGCA AGCAGCAGAT TACGCGCAGA

7401 AAAAAAGGAT CTCAAGAAGA TCCTTTGATC TTTTCTACGG GGTCTGACGC TCAGTGGAAC GAAAACTCAC GTTAAGGGAT TTTGGTCATG AGATTATCAA
7501 AAAGGATCTT CACCTAGATC CTTTTAAATT AAAAATGAAG TTTTAAATCA ATCTAAAGTA TATATGAGTA AACTTGGTCT GACAGTTACC AATGCTTAAT

AhdI bla

7601 CAGTGAGGCA CCTATCTCAG CGATCTGTCT ATTTCGTTCA TCCATAGTTG CCTGACTCCC CGTCGTGTAG ATAACTACGA TACGGGAGGG CTTACCATCT

bla

7701 GGCCCCAGTG CTGCAATGAT ACCGCGAGAC CCACGCTCAC CGGCTCCAGA TTTATCAGCA ATAAACCAGC CAGCCGGAAG GGCCGAGCGC AGAAGTGGTC

bla

7801 CTGCAACTTT ATCCGCCTCC ATCCAGTCTA TTAATTGTTG CCGGGAAGCT AGAGTAAGTA GTTCGCCAGT TAATAGTTTG CGCAACGTTG TTGCCATTGC

bla

7901 TACAGGCATC GTGGTGTCAC GCTCGTCGTT TGGTATGGCT TCATTCAGCT CCGGTTCCCA ACGATCAAGG CGAGTTACAT GATCCCCCAT GTTGTGCAAA

bla

8001 AAAGCGGTTA GCTCCTTCGG TCCTCCGATC GTTGTCAGAA GTAAGTTGGC CGCAGTGTTA TCACTCATGG TTATGGCAGC ACTGCATAAT TCTCTTACTG

bla

8101 TCATGCCATC CGTAAGATGC TTTTCTGTGA CTGGTGAGTA CTCAACCAAG TCATTCTGAG AATAGTGTAT GCGGCGACCG AGTTGCTCTT GCCCGGCGTC

bla

8201 AATACGGGAT AATACCGCGC CACATAGCAG AACTTTAAAA GTGCTCATCA TTGGAAAACG TTCTTCGGGG CGAAAACTCT CAAGGATCTT ACCGCTGTTG

bla

8301 AGATCCAGTT CGATGTAACC CACTCGTGCA CCCAACTGAT CTTCAGCATC TTTTACTTTC ACCAGCGTTT CTGGGTGAGC AAAAACAGGA AGGCAAAATG

bla

8401 CCGCAAAAAA GGGAATAAGG GCGACACGGA AATGTTGAAT ACTCATACTC TTCCTTTTTC AATATTATTG AAGCATTTAT CAGGGTTATT GTCTCATGAG

bla

8501 CGGATACATA TTTGAATGTA TTTAGAAAAA TAAACAAATA GGGGTTCCGC GCACATTTCC CCGAAAAGTG CCACCTGACG TCTAAGAAAC CATTATTATC
8601 ATGACATTAA CCTATAAAAA TAGGCGTATC ACGAGGCCCT TTCGTCTCGC GCGTTTCGGT GATGACGGTG AAAACCTCTG ACACATGCAG CTCCCGGAGA
8701 CGGTCACAGC TTGTCTGTAA GCGGATGCCG GGAGCAGACA AGCCCGTCAG GGCGCGTCAG CGGGTGTTGG CGGGTGTCGG GGCTGGCTTA ACTATGCGGC
8801 ATCAGAGCAG ATTGTACTGA GAGTGCACCA TATGCGGTGT GAAATACCGC ACAGATGCGT AAGGAGAAAA TACCGCATCA GGCGCCATTC GCCATTCAGG
8901 CTGCGCAACT GTTGGGAAGG GCGATCGGTG CGGGCCTCTT CGCTATTACG CCAGCTGGCG AAAGGGGGAT GTGCTGCAAG GCGATTAAGT T

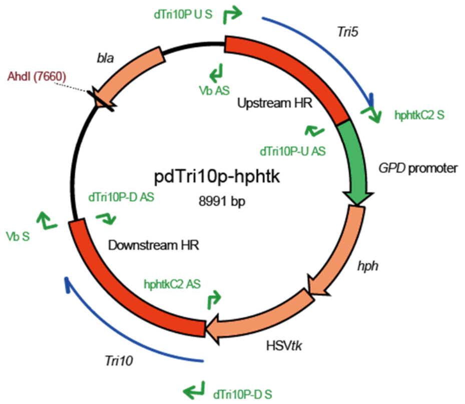

Tri5 partial CDS
SbfI Upstream HR
SwTri10P-U_S

1 GGGTAACGCC AGGGTTTTCC CAGTCACGAC GTTGTAAAAC GACGGCCAGT GCCAAGCTTG CATGCCTGCA GGTCGACTCT AGAGCAGTAC AACTTTGGAG

Vb_AS

Tri5 partial CDS
Upstream HR
SwTri10P-U_S

101 GATTCCCAGG ATCTGATGAC TACCCTCAAT TCCTTCGTCG TATGAATGGT TTGGGTCATT GTGTTGGGGC TTCTCTATGG CCCAAGGACC TGTTTGATGA

Upstream HR
Tri5 partial CDS

201 GCGGAAGCAT TTCCTTGAAA TCACGTCAGC CGTTGCTCAG ATGGAGAACT GGATGGTTTG GGTCAATGAT CTCATGTCAT TCTACAAGGA ATTCGACGAT

Upstream HR
Tri5 partial CDS

301 GAGCGTGACC AAATCAGTCT GGTCAAGAAC TTTGTCACCT GCCATGAGAT CACTCTGGAT GAAGCTTTGG AGAAGCTCAC CCAGGAAACC CTACACTCGT

Upstream HR
Tri5 partial CDS

401 CTAAGCAGAT GGTTGCTGTC TTCTCGGACA AGGACCCTCA GGTGATGGAC ACGATTGAGT GTTTCATGCA TGGCTACGTC ACGTGGCACT TGTGCGACGC

Upstream HR
Tri5 partial CDS

501 TCGATACCGC CTCCATGAGA TTTATGAAAA GGTCAAGGAT CAGGATACAG AGGACGCCAA GAAGTTCTGC AAGTTCTTTG AGCAGGCGGC CAATGTCGGC

Upstream HR
Tri5 partial CDS

601 GCCGTTGCAC CCTCGGAGTG GGCTTATCCA CAAGTTGCAC AACTGGCAAA CGTTCGGGCC AAGGACGATG TGAAGGAGGC TCAGAAGCCC ATCCTAAGTT

Tri10 promoter
Upstream HR
Tri5 partial CDS

701 CAATTGAGCT AGTGGAGTAA CCGAAGGCGA GTTTGGAAGT ATGTTTTGCG GGTACGGATA CTCGTTTGGA GAATGGTGGT CTGTTATAAT GATTACAAAT

Tri10 promoter

801 AGTTCGGTCG TGTTTTGTTA GAATGAACAG TTGAACAAGG ATAATTACTT CGGAATAGGC AGTTGAAACT GAATGTCTGT ACGTAACCTG AGCCTGTAAC

Tri10 promoter

901 CATTTCCCAC TTGAGTGCAG GCTTTTGCGT AACCAAGTCT GTACACCCGT CGGTGCGACA GGGCTACCCC CAACCCTGCA ACTGCATCTG CAGCTGCAGC

Tri10 promoter

1001 TGGCAGACTG GTAGACTGGC GCTACGAACT GTAGTGCGAT GCGGGAATCT TGTACCCGCT CGGAGGTTGG AGGGAGCTGG CACATTCTCT AGACCCGCGA

Tri10 promoter

1101 ATTGATCTTC AAAGCGCTTG CGTTTTGTCC AGATCAGTGA CCATACCCTT GCCTTTTCGC ACCACCCAAA CGTCCACTGA ACGAGGCGTA CAGAAACCAC

Tri10 promoter
1201 ACAAGATAAG GTTTGATGCC TGCTTGAGCA CTATGAGGGA CACGACACTT CTGTAAAACT CTATCCTTGC ATTATATTGT AACATCGTTT AACTTCTCCA

gpd10P_S
GPD promoter
Tri10 promoter
1301 CGCCCATTCC TTCCCGTCTT TCCCCCAACT TCAATTGTAT GCCAACCAAC AATCATCAAA TTATCATTAT TATTGTCGTT AGTCATCCCG GTGACTCTTT
JTri10P_AS

GPD promoter
gpd10P_S
1401 CTGGCATGCG GAGAGACGGA CGGACGCAGA GAGAAGGGCT GAGTAATAAG CGCCACTGCG CCAGACAGCT CTGGCGGCTC TGAGGTGCAG TGGATGATTA

GPD promoter
1501 TTAATCCGGG ACCGGCCGCC CCTCCGCCCC GAAGTGGAAA GGCTGGTGTG CCCCTCGTTG ACCAAGAATC TATTGCATCA TCGGAGAATA TGGAGCTTCA

GPD promoter
1601 TCGAATCACC GGCAGTAAGC GAAGGAGAAT GTGAAGCCAG GGGTGTATAG CCGTCGGCGA AATAGCATGC CATTAACCTA GGTACAGAAG TCCAATTGCT

GPD promoter
1701 TCCGATCTGG TAAAAGATTC ACGAGATAGT ACCTTCTCCG AAGTAGGTAG AGCGAGTACC CGGCGCGTAA GCTCCCTAAT TGGCCCATCC GGCATCTGTA

GPD promoter
1801 GGGCGTCCAA ATATCGTGCC TCTCCTGCTT TGCCCGGTGT ATGAAACCGG AAAGGCCGCT CAGGAGCTGG CCAGCGGCGC AGACCGGGAA CACAAGCTGG

GPD promoter
1901 CAGTCGACCC ATCCGGTGCT CTGCACTCGA CCTGCTGAGG TCCCTCAGTC CCTGGTAGGC AGCTTTGCCC CGTCTGTCCG CCCGGTGTGT CGGCGGGGTT

GPD promoter
2001 GACAAGGTCG TTGCGTCAGT CCAACATTTG TTGCCATATT TTCCTGCTCT CCCCACCAGC TGCTCTTTTC TTTTCTCTTT CTTTTCCCAT CTTCAGTATA

GPD promoter
2101 TTCATCTTCC CATCCAAGAA CCTTTATTTC CCCTAAGTAA GTACTTTGCT ACATCCATAC TCCATCCTTC CCATCCCTTA TTCCTTTGAA CCTTTCAGTT

Downstream HR
GPD promoter
Tri10
JTri10P_S
2201 CGAGCTTTCC CACTTCATCG CAGCTTGACT AACAGCTACC CCGCTTGAGA TGGATTTCCC AAAGCCTAGA CAAGTCCGAG AGACGAGCCT GTTGATGTAC
gpd10P_AS

Tri10
Downstream HR
2301 TACCTAGACG TCGTGTTTCC TCTGCAATGC ATCAACCCAA ACAACAATTG TCTGGGAAAG AGAGAGTGGC TGTTGACTAT ACTGACCTCT GCGCGGCCTA

Tri10
Downstream HR
2401 CGTACTATGC CACATTGTGC ATGTCGCTCC TCTATAAAGA ATCGCTTTCA AGCCCTTGCA GATCTGAACA GGCGATGGTA TGGAAGAGAG AGAAGACATA

Tri10
Downstream HR
2501 CTACTACATT CTTGCACTCC AGGAGTCTCA GAAGCTGCTG GGTGGGCTCG ACAAGACATT TGGCATCACA AGGCTGAAAG GTACCGTCGT TGCCCTTGCT

Tri10
Downstream HR
2601 TGCATGCTAC AGCTTATCAG TTTTGAGGTA AGACGAATCC ACCATTGTTT CGATGCTCGA TGTCGATGCT CGATATCCGA TCTACGATTA TCGTTGGTCA

Tri10
Downstream HR
2701 CTAACAAATT AAAATAGTCT TCGCACCTAA GCAGGGGAGA TTGGCGCGTT CACCTCCATG CGGCCAACAT ACTCATTCCT GTCTTGGTTG AGGGATGGTC

Tri10
Downstream HR
2801 CACAGCTTTG CAATCAGGCC CCCCAGCCAC CTCCATATGG TGCGAGCTGG ATGAATCACA CTTCGGCTCG ACTGAAGATC AAACCTCTTT GAGCTTCGAA

Tri10
Downstream HR
2901 TACGTCGGAG CTTTGAGATT CCTGTCAAAC TCACTCGCCG CAGTCGGCAT CCTGTCTTGC ATATCTATTG GCCCATCAGC ACCATTTGAA GATTACGGCC

Tri10
Downstream HR
3001 ATCTCCTGGA CCAGCCAGGC CTTATACAGC TGGACGAGGT GCTGGGGTGC AGGAATTGGA CCATGTTGAC TATTCTCGAA GTGGGTAAGC TGGATCGTTG

Tri10
Downstream HR
3101 GAAGCGACAG GAGCAAGAAC ATAATCGCTT GAGCCTAAAG ACGCTCGCTA GGCGCGCCAT GATGATTGAG GATATGTTGT CAGACGAGCT ACAAAGGCTA

Tri10
Downstream HR
3201 CCGACAGACG AGACGCTTCC AGACCTCATC ACTCAGATTT ACGCCGCCTC TATCATGACG TATCTGCATA CAGTAGTTTC CGGACTCAAT CCCAACCTTT

Tri10
Downstream HR
3301 CAGAGGTTCA GGATAGTGTG GCCGGGACGC TTCAATTGTT GGAGAGGCTC CCAAATCTTG AAGCTGTCAC GAGCGTTACT TGGCCTCTAG CTGTCACAGG

Tri10
Downstream HR
3401 ATGCATGGCC TCAGAAAGTC ATAAGGACTT TTTCAGAAAT ACTCTGAGGT CGTATGAGGC GACATTCAGC TCCTTAAAAA AGTATGACGG AACTCTTCAG

Tri10
Downstream HR
3501 GTCTTGGAAG ACGCTTGGAA GAGAAGAGAG ATAGATACAG AGTCTCCAAT GAGATGGGAA GACTTGACGG ATCACCATGG GCTTCCAGTG CTACTTTGGT

Tri10
Downstream HR
3601 AGGGATGGTA TCGGCGCAGA GACTACGGAT ACCGATACCC GAAGTCTTTC TTCTGCATCA GCTACCCGAT ACATGTAAGT TTGATGCTCA CTTACAGATG

Downstream HR
3701 ATGCATGCAG GGAAGTTTTA TTAGATGTTA TGGGTTCGAC TCATTTTGGC GTTTAAACAG GGATTGGCAT GATGCATGAT TTAAATGGAA TAGAAGAGCG

Downstream HR
3801 GAAAACGTTT CCCATGTAAC TTGAATGATG GACTTTCAAT ATATATTTCT AGTATACTTT TTTAACCGTG AACCGCATCT TCTCCATCTA TCCAACCAAT

Downstream HR
3901 CGTGCTGAAA GCAGTCTGGA ACCATGGTGG GCGAATCAGT GCTGTGTCCC TTGATTGTCT CGACATGGCT TGACATTGTT TAGCCCGTAC GACAAACGAT

Downstream HR
Vb_S
4001 ATTGATCATA GTGAAGGAGT GTTGATTTCA AGCGAGGGGA TTTGTCGAAG TTCGGGGTAC CGAGCTCGAA TTCGTAATCA TGGTCATAGC TGTTTCCTGT
dTri10P-D_AS

4101 GTGAAATTGT TATCCGCTCA CAATTCCACA CAACATACGA GCCGGAAGCA TAAAGTGTAA AGCCTGGGGT GCCTAATGAG TGAGCTAACT CACATTAATT
4201 GCGTTGCGCT CACTGCCCGC TTTCCAGTCG GGAAACCTGT CGTGCCAGCT GCATTAATGA ATCGGCCAAC GCGCGGGGAG AGGCGGTTTG CGTATTGGGC
4301 GCTCTTCCGC TTCCTCGCTC ACTGACTCGC TGCGCTCGGT CGTTCGGCTG CGGCGAGCGG TATCAGCTCA CTCAAAGGCG GTAATACGGT TATCCACAGA
4401 ATCAGGGGAT AACGCAGGAA AGAACATGTG AGCAAAAGGC CAGCAAAAGG CCAGGAACCG TAAAAAGGCC GCGTTGCTGG CGTTTTTCCA TAGGCTCCGC
4501 CCCCCTGACG AGCATCACAA AAATCGACGC TCAAGTCAGA GGTGGCGAAA CCCGACAGGA CTATAAAGAT ACCAGGCGTT TCCCCCTGGA AGCTCCCTCG
4601 TGCGCTCTCC TGTTCCGACC CTGCCGCTTA CCGGATACCT GTCCGCCTTT CTCCCTTCGG GAAGCGTGGC GCTTTCTCAT AGCTCACGCT GTAGGTATCT
4701 CAGTTCGGTG TAGGTCGTTC GCTCCAAGCT GGGCTGTGTG CACGAACCCC CCGTTCAGCC CGACCGCTGC GCCTTATCCG GTAACTATCG TCTTGAGTCC
4801 AACCCGGTAA GACACGACTT ATCGCCACTG GCAGCAGCCA CTGGTAACAG GATTAGCAGA GCGAGGTATG TAGGCGGTGC TACAGAGTTC TTGAAGTGGT
4901 GGCCTAACTA CGGCTACACT AGAAGGACAG TATTTGGTAT CTGCGCTCTG CTGAAGCCAG TTACCTTCGG AAAAAGAGTT GGTAGCTCTT GATCCGGCAA
5001 ACAAACCACC GCTGGTAGCG GTGGTTTTTT TGTTTGCAAG CAGCAGATTA CGCGCAGAAA AAAAGGATCT CAAGAAGATC CTTTGATCTT TTCTACGGGG
5101 TCTGACGCTC AGTGGAACGA AAACTCACGT TAAGGGATTT TGGTCATGAG ATTATCAAAA AGGATCTTCA CCTAGATCCT TTTAAATTAA AAATGAAGTT
5201 TTAAATCAAT CTAAAGTATA TATGAGTAAA CTTGGTCTGA CAGTTACCAA TGCTTAATCA GTGAGGCACC TATCTCAGCG ATCTGTCTAT TTCGTTCATC
bla
5301 CATAGTTGCC TGACTCCCCG TCGTGTAGAT AACTACGATA CGGGAGGGCT TACCATCTGG CCCCAGTGCT GCAATGATAC CGCGAGACCC ACGCTCACCG
bla
5401 GCTCCAGATT TATCAGCAAT AAACCAGCCA GCCGGAAGGG CCGAGCGCAG AAGTGGTCCT GCAACTTTAT CCGCCTCCAT CCAGTCTATT AATTGTTGCC
bla
5501 GGGAAGCTAG AGTAAGTAGT TCGCCAGTTA ATAGTTTGCG CAACGTTGTT GCCATTGCTA CAGGCATCGT GGTGTCACGC TCGTCGTTTG GTATGGCTTC
bla
5601 ATTCAGCTCC GGTTCCCAAC GATCAAGGCG AGTTACATGA TCCCCCATGT TGTGCAAAAA AGCGGTTAGC TCCTTCGGTC CTCCGATCGT TGTCAGAAGT
bla
5701 AAGTTGGCCG CAGTGTTATC ACTCATGGTT ATGGCAGCAC TGCATAATTC TCTTACTGTC ATGCCATCCG TAAGATGCTT TTCTGTGACT GGTGAGTACT
bla
5801 CAACCAAGTC ATTCTGAGAA TAGTGTATGC GGCGACCGAG TTGCTCTTGC CCGGCGTCAA TACGGGATAA TACCGCGCCA CATAGCAGAA CTTTAAAAGT
bla
5901 GCTCATCATT GGAAAACGTT CTTCGGGGCG AAAACTCTCA AGGATCTTAC CGCTGTTGAG ATCCAGTTCG ATGTAACCCA CTCGTGCACC CAACTGATCT
bla
6001 TCAGCATCTT TTACTTTCAC CAGCGTTTCT GGGTGAGCAA AAACAGGAAG GCAAAATGCC GCAAAAAAGG GAATAAGGGC GACACGGAAA TGTTGAATAC
bla
6101 TCATACTCTT CCTTTTTCAA TATTATTGAA GCATTTATCA GGGTTATTGT CTCATGAGCG GATACATATT TGAATGTATT TAGAAAAATA AACAAATAGG
bla
6201 GGTTCCGCGC ACATTTCCCC GAAAAGTGCC ACCTGACGTC TAAGAAACCA TTATTATCAT GACATTAACC TATAAAAATA GGCGTATCAC GAGGCCCTTT
6301 CGTCTCGCGC GTTTCGGTGA TGACGGTGAA AACCTCTGAC ACATGCAGCT CCCGGAGACG GTCACAGCTT GTCTGTAAGC GGATGCCGGG AGCAGACAAG
6401 CCCGTCAGGG CGCGTCAGCG GGTGTTGGCG GGTGTCGGGG CTGGCTTAAC TATGCGGCAT CAGAGCAGAT TGTACTGAGA GTGCACCATA TGCGGTGTGA
6501 AATACCGCAC AGATGCGTAA GGAGAAAATA CCGCATCAGG CGCCATTCGC CATTCAGGCT GCGCAACTGT TGGGAAGGGC GATCGGTGCG GGCCTCTTCG
6601 CTATTACGCC AGCTGGCGAA AGGGGGATGT GCTGCAAGGC GATTAAGTT

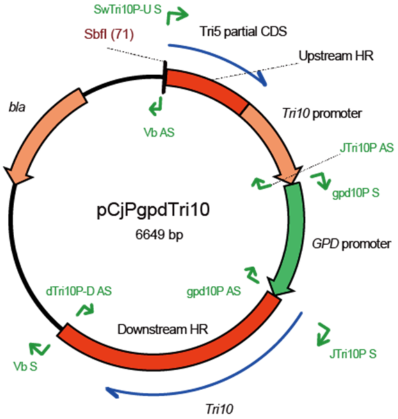

Upstream HR
Tri5 CDS partial
SbfI
SwTri10P-U_S
1 GGGTAACGCC AGGGTTTTCC CAGTCACGAC GTTGTAAAAC GACGGCCAGT GCCAAGCTTG CATGCCTGCA GGTCGACTCT AGAGCAGTAC AACTTTGGAG
Vb_AS

Upstream HR
Tri5 CDS partial
SwTri10P-U_S
101 GATTCCCAGG ATCTGATGAC TACCCTCAAT TCCTTCGTCG TATGAATGGT TTGGGTCATT GTGTTGGGGC TTCTCTATGG CCCAAGGACC TGTTTGATGA

Tri5 CDS partial
Upstream HR
201 GCGGAAGCAT TTCCTTGAAA TCACGTCAGC CGTTGCTCAG ATGGAGAACT GGATGGTTTG GGTCAATGAT CTCATGTCAT TCTACAAGGA ATTCGACGAT

Tri5 CDS partial
Upstream HR
301 GAGCGTGACC AAATCAGTCT GGTCAAGAAC TTTGTCACCT GCCATGAGAT CACTCTGGAT GAAGCTTTGG AGAAGCTCAC CCAGGAAACC CTACACTCGT

Tri5 CDS partial
Upstream HR
401 CTAAGCAGAT GGTTGCTGTC TTCTCGGACA AGGACCCTCA GGTGATGGAC ACGATTGAGT GTTTCATGCA TGGCTACGTC ACGTGGCACT TGTGCGACGC

Tri5 CDS partial
Upstream HR
501 TCGATACCGC CTCCATGAGA TTTATGAAAA GGTCAAGGAT CAGGATACAG AGGACGCCAA GAAGTTCTGC AAGTTCTTTG AGCAGGCGGC CAATGTCGGC

Tri5 CDS partial
Upstream HR
601 GCCGTTGCAC CCTCGGAGTG GGCTTATCCA CAAGTTGCAC AACTGGCAAA CGTTCGGGCC AAGGACGATG TGAAGGAGGC TCAGAAGCCC ATCCTAAGTT

Tri5 CDS partial
Tri10 promoter
Upstream HR

701 CAATTGAGCT AGTGGAGTAA CCGAAGGCGA GTTTGGAAGT ATGTTTTGCG GGTACGGATA CTCGTTTGGA GAATGGTGGT CTGTTATAAT GATTACAAAT

Tri10 promoter

801 AGTTCGGTCG TGTTTTGTTA GAATGAACAG TTGAACAAGG ATAATTACTT CGGAATAGGC AGTTGAAACT GAATGTCTGT ACGTAACCTG AGCCTGTAAC

Tri10 promoter

901 CATTTCCCAC TTGAGTGCAG GCTTTTGCGT AACCAAGTCT GTACACCCGT CGGTGCGACA GGGCTACCCC CAACCCTGCA ACTGCATCTG CAGCTGCAGC

Tri10 promoter

1001 TGGCAGACTG GTAGACTGGC GCTACGAACT GTAGTGCGAT GCGGGAATCT TGTACCCGCT CGGAGGTTGG AGGGAGCTGG CACATTCTCT AGACCCGCGA

Tri10 promoter

1101 ATTGATCTTC AAAGCGCTTG CGTTTTGTCC AGATCAGTGA CCATACCCTT GCCTTTTCGC ACCACCCAAA CGTCCACTGA ACGAGGCGTA CAGAAACCAC

Tri10 promoter

1201 ACAAGATAAG GTTTGATGCC TGCTTGAGCA CTATGAGGGA CACGACACTT CTGTAAAACT CTATCCTTGC ATTATATTGT AACATCGTTT AACTTCTCCA

tef10P_S
TEF promoter
Tri10 promoter

1301 CGCCCATTCC TTCCCGTCTT TCCCCCAACT TCAATTGTAT GCCAACCAAC AATCATCAAA TTATCATTAT TATTGTCGTT AGTCATCTAG CGACTTAGAG

JTri10P_AS

TEF promoter
tef10P_S

1401 TGCACGCTCA AAAGATACCC CTAACTTCTG ATTTGATAGC ATTTGTATAT GGCATATAGT ACGCCTGAAG CATAAAAAAA AAAACAGAAT TATTATAGTT

TEF promoter

1501 TATTAATACG AGACAGCAGA ATCACCGCCC AAGTTAAGCC TTTGTGCTGA TCATGCTCTC GAACGGGCCA AGTTCGGGAA AAGCAAAGGA GCGTTTAGTG

TEF promoter

1601 AGGGGCAATT TGACTCACCT CCCAGGCAAC AGATGAGGGG GGCAAAAAGA AAGAAATTTT CGTGAGTCAA TATGGATTCC GAGCATCATT TTCTTGCGGT

TEF promoter

1701 CTATCTTGCT ACGTATGTTG ATCTTGACGC TGTGGATCAA GCAACGCCAC TCGCTCGCTC CATCGCAGGC TGGTCGCAGA CAAATTAAAA GGCGGCAAAC

TEF promoter

1801 TCGTACAGCC GCGGGGTTGT CCGCTGCAAA GTACAGAGTG ATAAAAGCCG CCATGCGACC ATCAACGCGT TGATGCCCAG CTTTTTCGAT CCGAGAATCC

TEF promoter

1901 ACCGTAGAGG CGATAGCAAG TAAAGAAAAG CTAAACAAAA AAAAATTTCT GCCCCTAAGC CATGAAAACG AGATGGGGTG GAGCAGAACC AAGGAAAGAG

TEF promoter

2001 TCGCGCTGGG CTGCCGTTCC GGAAGGTGTT GTAAAGGCTC GACGCCCAAG GTGGGAGTCT AGGAGAAGAA TTTGCATCGG GAGTGGGGCG GGTTACCCCT

TEF promoter

2101 CCATATCCAA TGACAGATAT CTACCAGCCA AGGGTTTGAG CCCGCCCGCT TAGTCGTCGT CCTCGCTTGC CCCTCCATAA AAGGATTTCC CCTCCCCCTC

TEF promoter

2201 CCACAAAATT TTCTTTCCCT TCCTCTCCTT GTCCGCTTCA GTACGTATAT CTTCCCTTCC CTCGCTTCTC TCCTCCATCC TTCTTTCATC CATCTCCTGC

Downstream HR
Tri10
JTri10_S
TEF promoter

2301 TAACTTCTCT GCTCAGCACC TCTACGCATT ACTAGCCGTA GTATCTGAGC ACTTCTCCCT TTTATATTCC ACAAAACATA ACACAACCTT CACCATGGAT

tef10P_AS

Tri10
JTri10_S
Downstream HR

2401 TTCCCAAAGC CTAGACAAGT CCGAGAGACG AGCCTGTTGA TGTACTACCT AGACGTCGTG TTTCCTCTGC AATGCATCAA CCCAAACAAC AATTGTCTGG

tef10P_AS

Tri10
Downstream HR

2501 GAAAGAGAGA GTGGCTGTTG ACTATACTGA CCTCTGCGCG GCCTACGTAC TATGCCACAT TGTGCATGTC GCTCCTCTAT AAAGAATCGC TTTCAAGCCC

Tri10
Downstream HR

2601 TTGCAGATCT GAACAGGCGA TGGTATGGAA GAGAGAGAAG ACATACTACT ACATTCTTGC ACTCCAGGAG TCTCAGAAGC TGCTGGGTGG GCTCGACAAG

Tri10
Downstream HR

2701 ACATTTGGCA TCACAAGGCT GAAAGGTACC GTCGTTGCCC TTGCTTGCAT GCTACAGCTT ATCAGTTTTG AGGTAAGACG AATCCACCAT TGTTTCGATG

Tri10
Downstream HR

2801 CTCGATGTCG ATGCTCGATA TCCGATCTAC GATTATCGTT GGTCACTAAC AAATTAAAAT AGTCTTCGCA CCTAAGCAGG GGAGATTGGC GCGTTCACCT

Tri10
Downstream HR

2901 CCATGCGGCC AACATACTCA TTCCTGTCTT GGTTGAGGGA TGGTCCACAG CTTTGCAATC AGGCCCCCCA GCCACCTCCA TATGGTGCGA GCTGGATGAA

Tri10
Downstream HR

3001 TCACACTTCG GCTCGACTGA AGATCAAACC TCTTTGAGCT TCGAATACGT CGGAGCTTTG AGATTCCTGT CAAACTCACT CGCCGCAGTC GGCATCCTGT

Tri10
Downstream HR

3101 CTTGCATATC TATTGGCCCA TCAGCACCAT TTGAAGATTA CGGCCATCTC CTGGACCAGC CAGGCCTTAT ACAGCTGGAC GAGGTGCTGG GGTGCAGGAA

Tri10
Downstream HR

3201 TTGGACCATG TTGACTATTC TCGAAGTGGG TAAGCTGGAT CGTTGGAAGC GACAGGAGCA AGAACATAAT CGCTTGAGCC TAAAGACGCT CGCTAGGCGC

Tri10
Downstream HR

3301 GCCATGATGA TTGAGGATAT GTTGTCAGAC GAGCTACAAA GGCTACCGAC AGACGAGACG CTTCCAGACC TCATCACTCA GATTTACGCC GCCTCTATCA

Tri10
Downstream HR

3401 TGACGTATCT GCATACAGTA GTTTCCGGAC TCAATCCCAA CCTTTCAGAG GTTCAGGATA GTGTGGCCGG GACGCTTCAA TTGTTGGAGA GGCTCCCAAA

Tri10
Downstream HR

3501 TCTTGAAGCT GTCACGAGCG TTACTTGGCC TCTAGCTGTC ACAGGATGCA TGGCCTCAGA AAGTCATAAG GACTTTTTCA GAAATACTCT GAGGTCGTAT

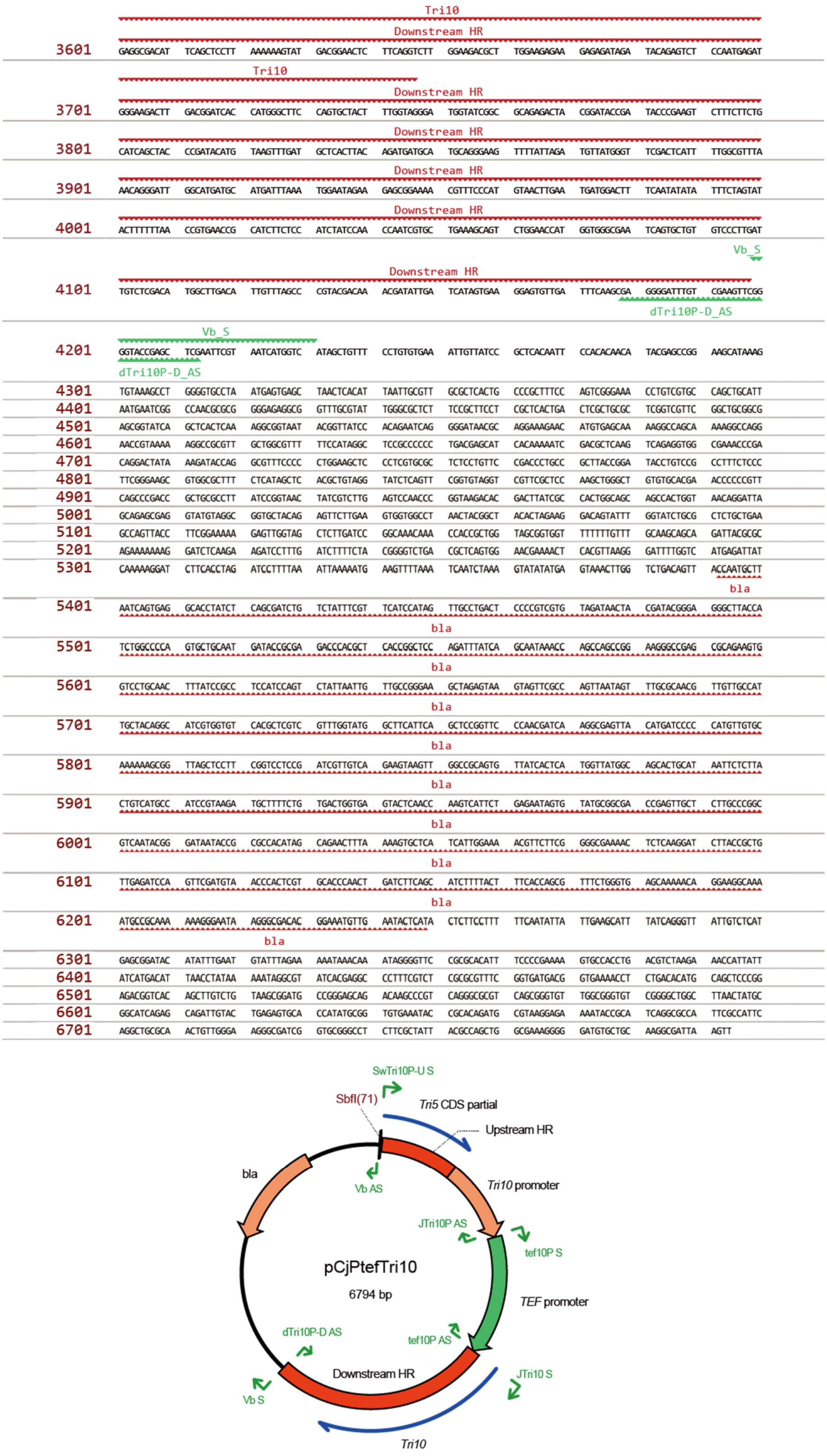

Supplementary Figure S6. Trichothecene production of transgenic *F. graminearum* strains with constitutive overexpression of *Tri6* and/or *Tri10*. (A) Pedigree of transformants with

multiple genetic manipulations for the overproduction of 15-ADON. The previously described strain Δ*Tri6* tk (YN_001) contains a hygromycin B phosphotransferase gene translationally fused to a herpes simplex virus thymidine kinase gene (*hph::tk*) [9, 10]. This strain was used to generate a marker-free *Tri6*-overexpressor strain YN_004 (Δ*Tri6* / P_{TEF}::*Tri6*) by conditional negative selection against the *hph::tk* cassette with 2′-deoxy-5-fluorouridine (5-FdU). In strain YN_004, a strong *Aspergillus nidulans TEF1α* promoter fragment [9] is inserted upstream of the *Tri6* coding region. The promoter disruptants YN_112 (Δ*Tri6* / P_{TEF}::*Tri6*, ΔP_{Tri10} / P_{GPD}::*hph::tk*) and YN_109 (ΔP_{Tri10} / P_{GPD}::*hph::tk*) were obtained from YN_004 and the WT strains, respectively, by positive selection against *hph::tk* with hygromycin B. Marker-free transformants YN_120 (Δ*Tri6* / P_{TEF}::*Tri6*, ΔP_{Tri10} / P_{GPD}) and YN_116 (ΔP_{Tri10} / P_{Tri10} + P_{TEF}) were obtained from YN_112 and YN_109, respectively, by the negative selection. (B) Generation of strains YN_120 and YN_116. YN_004 was created from YN_001 following transformation with pCjTefTri6, where 5-FdU resistant transformants with double crossover homologous recombination were negatively selected against *hph::tk*. YN_112 and YN_109 were created from YN_004 and the WT strains, respectively, by positive selection with hygromycin B following transformation with pdTri10p-hphtk. YN_120 and YN_116 were created by negative selection with 5-FdU, respectively, following transformation of YN_112 with pCjPgpdTri10 and YN_109 with pCjPtefTri10. (C) Production of trichothecenes by the transgenic strains. The WT (JCM 9873), YN_004, YN_116, and YN_120 strains were cultured on 100 mL of YS_60 (0.1% [w/v] Bacto™ yeast extract, 6% [w/v] sucrose) and YG_60 (0.1% [w/v] Bacto™ yeast extract, 6% [w/v] glucose) medium in a 300 mL-Erlenmeyer flask with gyratory shaking at 25°C. In contrast to the WT and *Tri6*-overexpressor YN_004 strains, YN_116 produced 15-ADON on YG_60 medium that does not contain sucrose. The 15-ADON production was further enhanced in the culture of YN_120, in which both *Tri6* and *Tri10* were constitutively overexpressed. YN_120 also produced 15-ADON on YG medium (0.5% [w/v] Bacto™ yeast extract, 2% [w/v] glucose) that contains a lower amount of glucose and a higher amount of yeast extract. (D) Sequences and maps of disruption and replacement vectors.

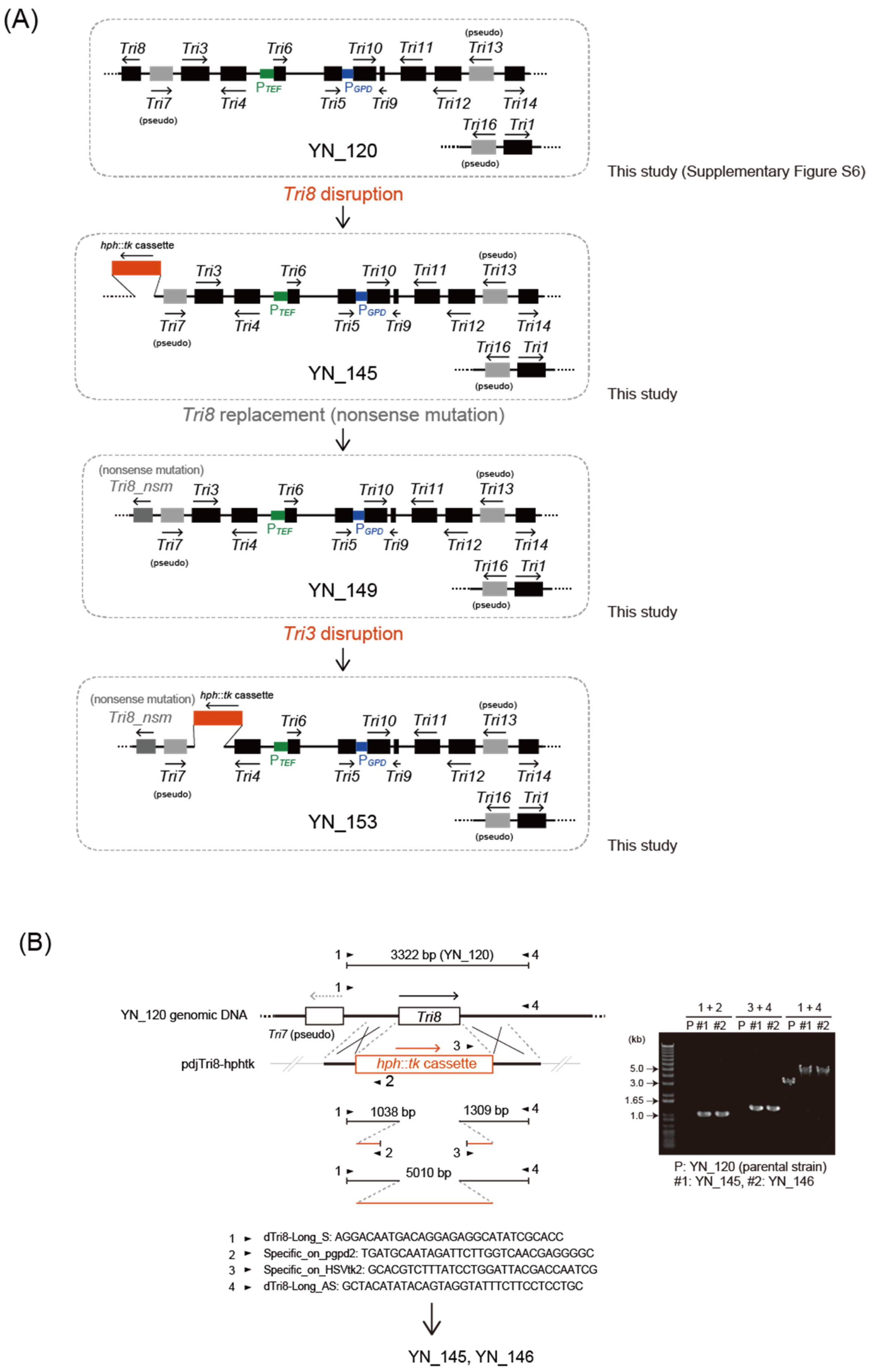
(A)
Tri8 disruption
Tri8 replacement (nonsense mutation)
Tri3 disruption
YN_120
YN_145
YN_149
YN_153
This study (Supplementary Figure S6)
This study
This study
This study
hph::tk cassette
(nonsense mutation)
Tri8_nsm
(B)
3322 bp (YN_120)
YN_120 genomic DNA
Tri7 (pseudo)
Tri8
pdjTri8-hphtk
hph::tk cassette
1038 bp
1309 bp
5010 bp
P: YN_120 (parental strain)
#1: YN_145, #2: YN_146
1 ► dTri8-Long_S: AGGACAATGACAGGAGAGGCATATCGCACC
2 ► Specific_on_pgpd2: TGATGCAATAGATTCTTGGTCAACGAGGGGC
3 ► Specific_on_HSVtk2: GCACGTCTTTATCCTGGATTACGACCAATCG
4 ► dTri8-Long_AS: GCTACATATACAGTAGGTATTTCTTCCTCCTGC
YN_145, YN_146

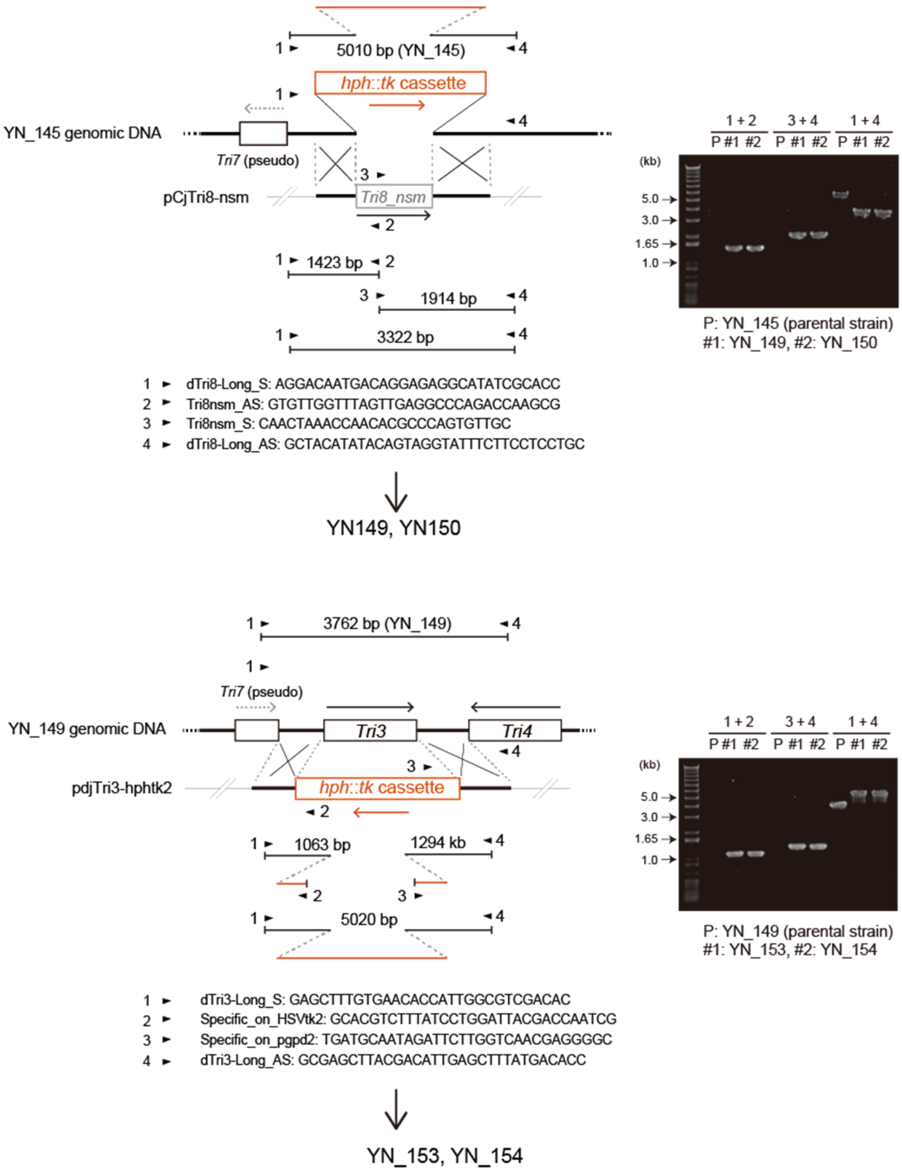

(C)

Tri8_Upstream
HindIII dTri8-U_S
1 GGGTAACGCC AGGGTTTTCC CAGTCACGAC GTTGTAAAAC GACGGCCAGT GCCAAGCTTG CATGCCTGCA GGTCGACTCT AGAGTCTACC TGCGATCCGA
Vb_AS

Tri8_Upstream
dTri8-U_S
101 TACCTATCGC ATTGTCGCTG GTGCTGGAAA AGATTCCGTC CGACTTTCGG TCCGATTAGA ACTATTGCGA AGGCCGTGAA AATGAACCGC TTGATGTACA

Tri8_Upstream
201 AAGACTAGAG GTCCAGACAA GTAAGTGAAA GAGCAATACA AGACAGATGA AGCCTTACTT TTCTCAGGCC TCGCTGGTGG ACCTAGTAAA AAGCAAAATC

Tri8_Upstream
301 TCGAAACGAA CCAGCTGTGA TGTGTAGAAT GGAGGGCCTG ATGAAAGGGC CAGGGGCCTG ACATGGACAT GGGGACAATA AATCGGCTGT TCCAATTATA

Tri8_Upstream
401 CGTAACTACA CTTAAAGAAC CCAGGACTCA ATCTAGAATT CTTTGATAAA GCGAGCTTCA CTTTACTATG TATAAAGTAA AGTTTGTAGT GTATACGTCC

Tri8_Upstream
501 TTAGCCCTTC CTCATTTGAT TACAGTTCTA ATTCTTGTAA GACTTGGTGA TGGTTAGGAG CCCCCTGTAA GTTTGTGCGC TCCTATGGCT CAAAATGACT

Tri8_Upstream
601 CATTTCACTC TAGCGCATTG AGGATTGATG TACTCTGTAC GTGCTGAGGT CTTGGACCCC TTCACCCCTG GCACAACAAC ATCAAGGTTC AAACAACATT

GPD promoter
Tri8_Upstream
hphtkC2_S
701 ACTAAATAAA ACGATGCGCC GCGTTTCGTA GTGTGAGTCC CAAAGAGCTA TTGACAGTTC AAGTCATTCA TTCGTTCACT CACTCAGTCC CCCGGTGACT
dTri8-U_AS

GPD promoter
hphtkC2_S
801 CTTTCTGGCA TGCGGAGAGA CGGACGGACG CAGAGAGAAG GGCTGAGTAA TAAGCGCCAC TGCGCCAGAC AGCTCTGGCG GCTCTGAGGT GCAGTGGATG
dTri8-U_AS

GPD promoter
901 ATTATTAATC CGGGACCGGC CGCCCCTCCG CCCCGAAGTG GAAAGGCTGG TGTGCCCCTC GTTGACCAAG AATCTATTGC ATCATCGGAG AATATGGAGC

GPD promoter
1001 TTCATCGAAT CACCGGCAGT AAGCGAAGGA GAATGTGAAG CCAGGGGTGT ATAGCCGTCG GCGAAATAGC ATGCCATTAA CCTAGGTACA GAAGTCCAAT

GPD promoter
1101 TGCTTCCGAT CTGGTAAAAG ATTCACGAGA TAGTACCTTC TCCGAAGTAG GTAGAGCGAG TACCCGGCGC GTAAGCTCCC TAATTGGCCC ATCCGGCATC

GPD promoter
1201 TGTAGGGCGT CCAAATATCG TGCCTCTCCT GCTTTGCCCG GTGTATGAAA CCGGAAAGGC CGCTCAGGAG CTGGCCAGCG GCGCAGACCG GGAACACAAG

GPD promoter
1301 CTGGCAGTCG ACCCATCCGG TGCTCTGCAC TCGACCTGCT GAGGTCCCTC AGTCCCTGGT AGGCAGCTTT GCCCCGTCTG TCCGCCCGGT GTGTCGGCGG

GPD promoter
1401 GGTTGACAAG GTCGTTGCGT CAGTCCAACA TTTGTTGCCA TATTTTCCTG CTCTCCCCAC CAGCTGCTCT TTTCTTTTCT CTTTCTTTTC CCATCTTCAG

GPD promoter
1501 TATATTCATC TTCCCATCCA AGAACCTTTA TTTCCCCTAA GTAAGTACTT TGCTACATCC ATACTCCATC CTTCCCATCC CTTATTCCTT TGAACCTTTC

GPD promoter hph
1601 AGTTCGAGCT TTCCCACTTC ATCGCAGCTT GACTAACAGC TACCCCGCTT GAGATCGATA TGAAAAAGCC TGAACTCACC GCGACGTCTG TCGAGAAGTT

hph
1701 TCTGATCGAA AAGTTCGACA GCGTCTCCGA CCTGATGCAG CTCTCGGAGG GCGAAGAATC TCGTGCTTTC AGCTTCGATG TAGGAGGGCG TGGATATGTC

hph
1801 CTGCGGGTAA ATAGCTGCGC CGATGGTTTC TACAAAGATC GTTATGTTTA TCGGCACTTT GCATCGGCCG CGCTCCCGAT TCCGGAAGTG CTTGACATTG

hph
1901 GGGAGTTCAG CGAGAGCCTG ACCTATTGCA TCTCCCGCCG TGCACAGGGT GTCACGTTGC AAGACCTGCC TGAAACCGAA CTGCCCGCTG TTCTCCAGCC

hph
2001 GGTCGCGGAG GCCATGGATG CGATCGCTGC GGCCGATCTT AGCCAGACGA GCGGGTTCGG CCCATTCGGA CCGCAAGGAA TCGGTCAATA CACTACATGG

hph
2101 CGTGATTTCA TATGCGCGAT TGCTGATCCC CATGTGTATC ACTGGCAAAC TGTGATGGAC GACACCGTCA GTGCGTCCGT CGCGCAGGCT CTCGATGAGC

hph
2201 TGATGCTTTG GGCCGAGGAC TGCCCCGAAG TCCGGCACCT CGTGCATGCG GATTTCGGCT CCAACAATGT CCTGACGGAC AATGGCCGCA TAACAGCGGT

hph
2301 CATTGACTGG AGCGAGGCGA TGTTCGGGGA TTCCCAATAC GAGGTCGCCA ACATCCTCTT CTGGAGGCCG TGGTTGGCTT GTATGGAGCA GCAGACGCGC

hph
2401 TACTTCGAGC GGAGGCATCC GGAGCTTGCA GGATCGCCGC GCCTCCGGGC GTATATGCTC CGCATTGGTC TTGACCAACT CTATCAGAGC TTGGTTGACG

hph
2501 GCAATTTCGA TGATGCAGCT TGGGCGCAGG GTCGATGCGA CGCAATCGTC CGATCCGGAG CCGGGACTGT CGGGCGTACA CAAATCGCCC GCAGAAGCGC

HSVtk
hph
2601 GGCCGTCTGG ACCGATGGCT GTGTAGAAGT ACTCGCCGAT AGTGGAAACC GACGCCCCAG CACTCGTCCG AGGGCAAAGG AAATGGCTTC GTACCCCGGC

HSVtk
2701 CATCAACACG CGTCTGCGTT CGACCAGGCT GCGCGTTCTC GCGGCCATAG CAACCGACGT ACGGCGTTGC GCCCTCGCCG GCAGCAAGAA GCCACGGAAG

HSVtk
2801 TCCGCCCGGA GCAGAAAATG CCCACGCTAC TGCGGGTTTA TATAGACGGT CCCCACGGGA TGGGGAAAAC CACCACCACG CAACTGCTGG TGGCCCTGGG

HSVtk
2901 TTCGCGCGAC GATATCGTCT ACGTACCCGA GCCGATGACT TACTGGCGGG TGCTGGGGGC TTCCGAGACA ATCGCGAACA TCTACACCAC ACAACACCGC

HSVtk
3001 CTCGACCAGG GTGAGATATC GGCCGGGGAC GCGGCGGTGG TAATGACAAG CGCCCAGATA ACAATGGGCA TGCCTTATGC CGTGACCGAC GCCGTTCTGG

HSVtk
3101 CTCCTCATAT CGGGGGGGAG GCTGGGAGCT CACATGCCCC GCCCCCGGCC CTCACCCTCA TCTTCGACCG CCATCCCATC GCCGCCCTCC TGTGCTACCC

HSVtk
3201 GGCCGCGCGG TACCTTATGG GCAGCATGAC CCCCCAGGCC GTGCTGGCGT TCGTGGCCCT CATCCCGCCG ACCTTGCCCG GCACCAACAT CGTGCTTGGG

HSVtk
3301 GCCCTTCCGG AGGACAGACA CATCGACCGC CTGGCCAAAC GCCAGCGCCC CGGCGAGCGG CTGGACCTGG CTATGCTGGC TGCGATTCGC CGCGTTTACG

HSVtk
3401 GGCTACTTGC CAATACGGTG CGGTATCTGC AGTGCGGCGG GTCGTGGCGG GAGGACTGGG GACAGCTTTC GGGGACGGCC GTGCCGCCCC AGGGTGCCGA

HSVtk
3501 GCCCCAGAGC AACGCGGGCC CACGACCCCA TATCGGGGAC ACGTTATTTA CCCTGTTTCG GGCCCCCGAG TTGCTGGCCC CCAACGGCGA CCTGTATAAC

HSVtk
3601 GTGTTTGCCT GGGCCTTGGA CGTCTTGGCC AAACGCCTCC GTTCCATGCA CGTCTTTATC CTGGATTACG ACCAATCGCC CGCCGGCTGC CGGGACGCCC

HSVtk
dTri8-D_S
3701 TGCTGCAACT TACCTCCGGG ATGGTCCAGA CCCACGTCAC CACCCCCGGC TCCATACCGA CGATATGCGA CCTGGCGCGC ACGTTTGCCC GGGAGATGGG
hphtkC2_AS

HSVtk Tri8_Downstream
dTri8-D_S
3801 GGAGGCTAAC TGACTTTCAT TTCTTGACAT CTCTGGCCAT CGGCCTGGTA ATTTATTTTT GGACTGCCGG TCATCGACTT GGTACGCCAT CCCTTATATG
hphtkC2_AS

Tri8_Downstream
3901 TTTGGCCAGA ACCTGTAGTG GTCTTATACT GTGTCTTATA AAATATTTAT TGATCCTTTT CTATGAGTAA CTTTATCTGT CTCCGGCGAT GTAGCTGCAT

Tri8_Downstream
4001 CTGTAAGATT CAAATAAATC ATCCAAACAC CAGCCAGGTA GCATTAATCG AAGAGGACCT CCAATCTGAA TAACTTCGGT GTTGTTGAGT ATTCCTGGTT

Tri8_Downstream
4101 TATTGAGATG GCTTTGATCA GCTCCCATTT CCCTTCAAGT TCCCTCCATT GTGGTCAGAA GACAAGGGAA GCATGAGGGT GGACTCATTT GAAGCACGAT

Tri8_Downstream
4201 AATTACACTA TCATAAAACA CAGGTTTAGC TTGAACTGAA AAACCACTCA TCACATTCAC AGTAGCACCA CTGGATAGAT GTTGGGTTCA AGTAAGCCTC

Tri8_Downstream
4301 ATGTTGCTCT GGCATCGCTA CTTACCCCTT GCCGCAACAA TATGCATCTT TTACACTTCA TGCACAAACA AGGGCATGGT ATCGTTGAGG CAATATCAGA

Tri8_Downstream
4401 CATATAAATT AATTATAATA TTGTCGGATA CTGCGTGGAG GAAGGCGACT TCGTCTTTAT GGACGTCAAG CCCTCCTGTA AGCCTCTTTT CAAGGTCCCT

Tri8_Downstream
4501 CAGGCTCGCA AGCTCCTTCT CAAGCTCTGG CACTATCCGG CCTAGCTTCT TAGATCGAAA GCGCCTTACG AAGAGCAAGA GGACCTTGTA GCTGAAGGCA

Tri8_Downstream
4601 ATTAACCTGA TGAGGCTGTA GTTATCGTCA TATGGCCCCT CTCGGAGGCA CCTTACCTTA TGCCTTAGCT ACTTTAGATC CTTACTGTAG TTACTAAGTA

Tri8_Downstream
4701 AGATAACCTG GTTTATGCCT TCTTTAGTAA AGAAATAGTC TTCTAGATAG CTAGGGATAT AACTGTATAG GCTTTTTTAT TGAGGCTGTA TTATTATATA

Tri8_Downstream
Vb_S
4801 ATACCTATGA CATCGGTTAG CTATATGGAG CGATGGACTG GGGTACCGAG CTCGAATTCG TAATCATGGT CATAGCTGTT TCCTGTGTGA AATTGTTATC
dTri8-D_AS

4901 CGCTCACAAT TCCACACAAC ATACGAGCCG GAAGCATAAA GTGTAAAGCC TGGGGTGCCT AATGAGTGAG CTAACTCACA TTAATTGCGT TGCGCTCACT
5001 GCCCGCTTTC CAGTCGGGAA ACCTGTCGTG CCAGCTGCAT TAATGAATCG GCCAACGCGC GGGGAGAGGC GGTTTGCGTA TTGGGCGCTC TTCCGCTTCC
5101 TCGCTCACTG ACTCGCTGCG CTCGGTCGTT CGGCTGCGGC GAGCGGTATC AGCTCACTCA AAGGCGGTAA TACGGTTATC CACAGAATCA GGGGATAACG
5201 CAGGAAAGAA CATGTGAGCA AAAGGCCAGC AAAAGGCCAG GAACCGTAAA AAGGCCGCGT TGCTGGCGTT TTTCCATAGG CTCCGCCCCC CTGACGAGCA
5301 TCACAAAAAT CGACGCTCAA GTCAGAGGTG GCGAAACCCG ACAGGACTAT AAAGATACCA GGCGTTTCCC CCTGGAAGCT CCCTCGTGCG CTCTCCTGTT
5401 CCGACCCTGC CGCTTACCGG ATACCTGTCC GCCTTTCTCC CTTCGGGAAG CGTGGCGCTT TCTCATAGCT CACGCTGTAG GTATCTCAGT TCGGTGTAGG
5501 TCGTTCGCTC CAAGCTGGGC TGTGTGCACG AACCCCCCGT TCAGCCCGAC CGCTGCGCCT TATCCGGTAA CTATCGTCTT GAGTCCAACC CGGTAAGACA
5601 CGACTTATCG CCACTGGCAG CAGCCACTGG TAACAGGATT AGCAGAGCGA GGTATGTAGG CGGTGCTACA GAGTTCTTGA AGTGGTGGCC TAACTACGGC
5701 TACACTAGAA GGACAGTATT TGGTATCTGC GCTCTGCTGA AGCCAGTTAC CTTCGGAAAA AGAGTTGGTA GCTCTTGATC CGGCAAACAA ACCACCGCTG
5801 GTAGCGGTGG TTTTTTTGTT TGCAAGCAGC AGATTACGCG CAGAAAAAAA GGATCTCAAG AAGATCCTTT GATCTTTTCT ACGGGGTCTG ACGCTCAGTG
5901 GAACGAAAAC TCACGTTAAG GGATTTTGGT CATGAGATTA TCAAAAAGGA TCTTCACCTA GATCCTTTTA AATTAAAAAT GAAGTTTTAA ATCAATCTAA
6001 AGTATATATG AGTAAACTTG GTCTGACAGT TACCAATGCT TAATCAGTGA GGCACCTATC TCAGCGATCT GTCTATTTCG TTCATCCATA GTTGCCTGAC
bla

6101 TCCCCGTCGT GTAGATAACT ACGATACGGG AGGGCTTACC ATCTGGCCCC AGTGCTGCAA TGATACCGCG AGACCCACGC TCACCGGCTC CAGATTTATC
bla

6201 AGCAATAAAC CAGCCAGCCG GAAGGGCCGA GCGCAGAAGT GGTCCTGCAA CTTTATCCGC CTCCATCCAG TCTATTAATT GTTGCCGGGA AGCTAGAGTA
bla

6301 AGTAGTTCGC CAGTTAATAG TTTGCGCAAC GTTGTTGCCA TTGCTACAGG CATCGTGGTG TCACGCTCGT CGTTTGGTAT GGCTTCATTC AGCTCCGGTT
bla

6401 CCCAACGATC AAGGCGAGTT ACATGATCCC CCATGTTGTG CAAAAAAGCG GTTAGCTCCT TCGGTCCTCC GATCGTTGTC AGAAGTAAGT TGGCCGCAGT
bla

6501 GTTATCACTC ATGGTTATGG CAGCACTGCA TAATTCTCTT ACTGTCATGC CATCCGTAAG ATGCTTTTCT GTGACTGGTG AGTACTCAAC CAAGTCATTC
bla

6601 TGAGAATAGT GTATGCGGCG ACCGAGTTGC TCTTGCCCGG CGTCAATACG GGATAATACC GCGCCACATA GCAGAACTTT AAAAGTGCTC ATCATTGGAA
bla

6701 AACGTTCTTC GGGGCGAAAA CTCTCAAGGA TCTTACCGCT GTTGAGATCC AGTTCGATGT AACCCACTCG TGCACCCAAC TGATCTTCAG CATCTTTTAC
bla

6801 TTTCACCAGC GTTTCTGGGT GAGCAAAAAC AGGAAGGCAA AATGCCGCAA AAAAGGGAAT AAGGGCGACA CGGAAATGTT GAATACTCAT ACTCTTCCTT
bla

6901 TTTCAATATT ATTGAAGCAT TTATCAGGGT TATTGTCTCA TGAGCGGATA CATATTTGAA TGTATTTAGA AAAATAAACA AATAGGGGTT CCGCGCACAT
7001 TTCCCCGAAA AGTGCCACCT GACGTCTAAG AAACCATTAT TATCATGACA TTAACCTATA AAAATAGGCG TATCACGAGG CCCTTTCGTC TCGCGCGTTT
7101 CGGTGATGAC GGTGAAAACC TCTGACACAT GCAGCTCCCG GAGACGGTCA CAGCTTGTCT GTAAGCGGAT GCCGGGAGCA GACAAGCCCG TCAGGGCGCG
7201 TCAGCGGGTG TTGGCGGGTG TCGGGGCTGG CTTAACTATG CGGCATCAGA GCAGATTGTA CTGAGAGTGC ACCATATGCG GTGTGAAATA CCGCACAGAT
7301 GCGTAAGGAG AAAATACCGC ATCAGGCGCC ATTCGCCATT CAGGCTGCGC AACTGTTGGG AAGGGCGATC GGTGCGGGCC TCTTCGCTAT TACGCCAGCT
7401 GGCGAAAGGG GGATGTGCTG CAAGGCGATT AAGTT

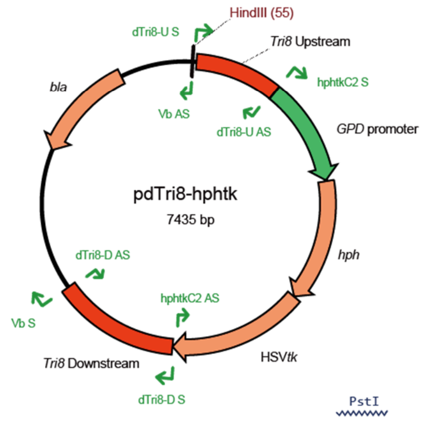

PstI Tri8_Upstream
dTri8-U_S
1 GGGTAACGCC AGGGTTTTCC CAGTCACGAC GTTGTAAAAC GACGGCCAGT GCCAAGCTTG CATGCCTGCA GGTCGACTCT AGAGTCTACC TGCGATCCGA
Vb_AS

Tri8_Upstream
dTri8-U_S
101 TACCTATCGC ATTGTCGCTG GTGCTGGAAA AGATTCCGTC CGACTTTCGG TCCGATTAGA ACTATTGCGA AGGCCGTGAA AATGAACCGC TTGATGTACA

Tri8_Upstream
201 AAGACTAGAG GTCCAGACAA GTAAGTGAAA GAGCAATACA AGACAGATGA AGCCTTACTT TTCTCAGGCC TCGCTGGTGG ACCTAGTAAA AAGCAAAATC

Tri8_Upstream
301 TCGAAACGAA CCAGCTGTGA TGTGTAGAAT GGAGGGCCTG ATGAAAGGGC CAGGGGCCTG ACATGGACAT GGGGACAATA AATCGGCTGT TCCAATTATA

Tri8_Upstream
401 CGTAACTACA CTTAAAGAAC CCAGGACTCA ATCTAGAATT CTTTGATAAA GCGAGCTTCA CTTTACTATG TATAAAGTAA AGTTTGTAGT GTATACGTCC

Tri8_Upstream
501 TTAGCCCTTC CTCATTTGAT TACAGTTCTA ATTCTTGTAA GACTTGGTGA TGGTTAGGAG CCCCCTGTAA GTTTGTGCGC TCCTATGGCT CAAAATGACT

Tri8_Upstream
601 CATTTCACTC TAGCGCATTG AGGATTGATG TACTCTGTAC GTGCTGAGGT CTTGGACCCC TTCACCCCTG GCACAACAAC ATCAAGGTTC AAACAACATT

Tri8-nsm
Tri8_Upstream
+3 M A L D
701 ACTAAATAAA ACGATGCGCC GCGTTTCGTA GTGTGAGTCC CAAAGAGCTA TTGACAGTTC AAGTCATTCA TTCGTTCACT CACTCAGTAT GGCTCTCGAT

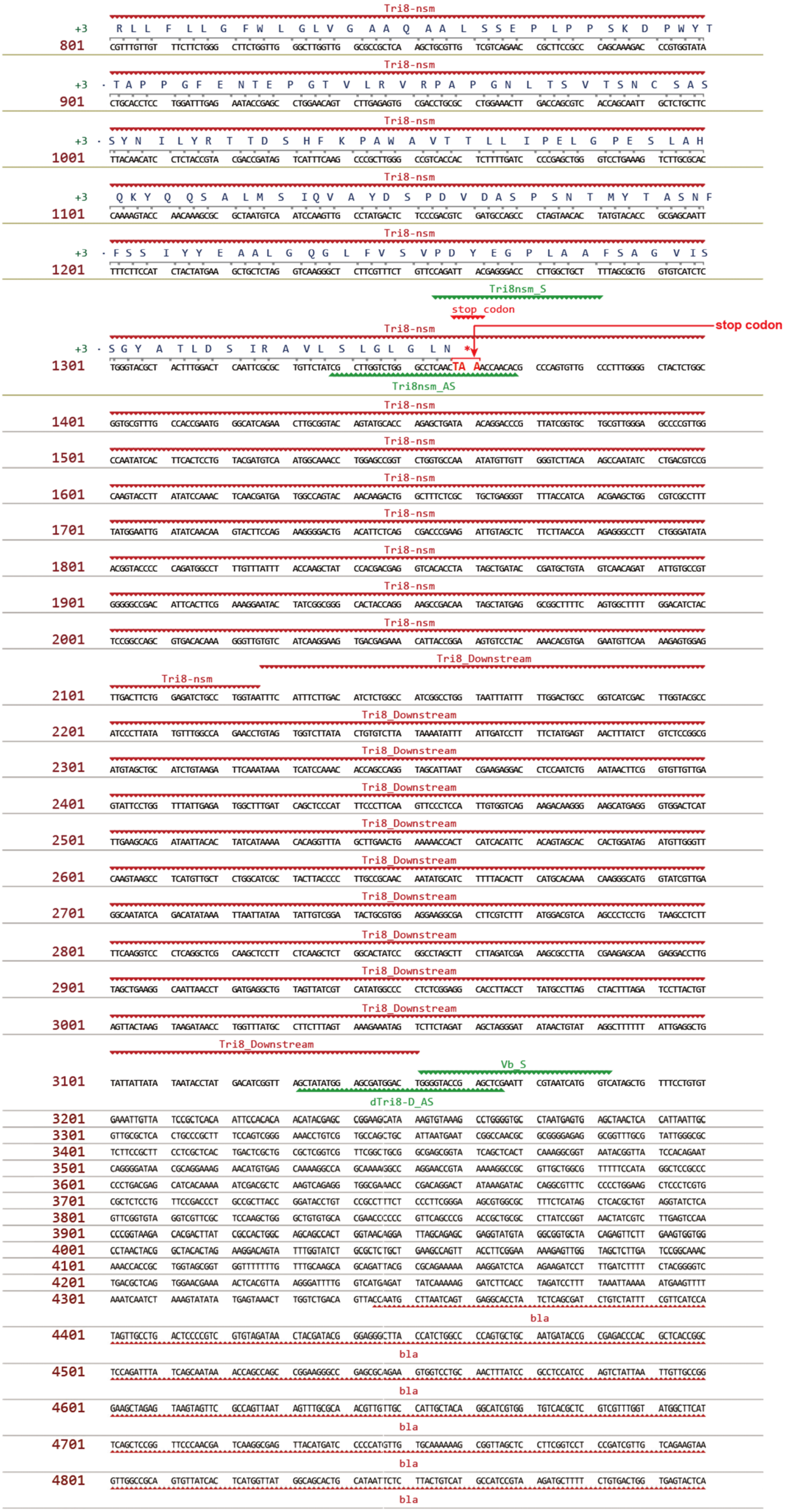
Tri8-nsm
+3 R L L F L L G F W L G L V G A A Q A A L S S E P L P P S K D P W Y T
801 CGTTTGTTGT TTCTTCTGGG CTTCTGGTTG GGCTTGGTTG GCGCCGCTCA AGCTGCGTTG TCGTCAGAAC CGCTTCCGCC CAGCAAAGAC CCGTGGTATA
Tri8-nsm
+3 T A P P G F E N T E P G T V L R V R P A P G N L T S V T S N C S A S
901 CTGCACCTCC TGGATTTGAG AATACCGAGC CTGGAACAGT CTTGAGAGTG CGACCTGCGC CTGGAAACTT GACCAGCGTC ACCAGCAATT GCTCTGCTTC
Tri8-nsm
+3 S Y N I L Y R T T D S H F K P A W A V T T L L I P E L G P E S L A H
1001 TTACAACATC CTCTACCGTA CGACCGATAG TCATTTCAAG CCCGCTTGGG CCGTCACCAC TCTTTTGATC CCCGAGCTGG GTCCTGAAAG TCTTGCGCAC
Tri8-nsm
+3 Q K Y Q Q S A L M S I Q V A Y D S P D V D A S P S N T M Y T A S N F
1101 CAAAAGTACC AACAAAGCGC GCTAATGTCA ATCCAAGTTG CCTATGACTC TCCCGACGTC GATGCCAGCC CTAGTAACAC TATGTACACC GCGAGCAATT
Tri8-nsm
+3 F S S I Y Y E A A L G Q G L F V S V P D Y E G P L A A F S A G V I S
1201 TTTCTTCCAT CTACTATGAA GCTGCTCTAG GTCAAGGGCT CTTCGTTTCT GTTCCAGATT ACGAGGGACC CTTGGCTGCT TTTAGCGCTG GTGTCATCTC
Tri8nsm_S
stop codon
Tri8-nsm
stop codon
+3 S G Y A T L D S I R A V L S L G L G L N *
1301 TGGGTACGCT ACTTTGGACT CAATTCGCGC TGTTCTATCG CTTGGTCTGG GCCTCAACTA AACCAACACG CCCAGTGTTG CCCTTTGGGG CTACTCTGGC
Tri8nsm_AS
Tri8-nsm
1401 GGTGCGTTTG CCACCGAATG GGCATCAGAA CTTGCGGTAC AGTATGCACC AGAGCTGATA ACAGGACCCG TTATCGGTGC TGCGTTGGGA GCCCCGTTGG
Tri8-nsm
1501 CCAATATCAC TTCACTCCTG TACGATGTCA ATGGCAAACC TGGAGCCGGT CTGGTGCCAA ATATGTTGTT GGGTCTTACA AGCCAATATC CTGACGTCCG
Tri8-nsm
1601 CAAGTACCTT ATATCCAAAC TCAACGATGA TGGCCAGTAC AACAAGACTG GCTTTCTCGC TGCTGAGGGT TTTACCATCA ACGAAGCTGG CGTCGCCTTT
Tri8-nsm
1701 TATGGAATTG ATATCAACAA GTACTTCCAG AAGGGGACTG ACATTCTCAG CGACCCGAAG ATTGTAGCTC TTCTTAACCA AGAGGGCCTT CTGGGATATA
Tri8-nsm
1801 ACGGTACCCC CAGATGGCCT TTGTTTATTT ACCAAGCTAT CCACGACGAG GTCACACCTA TAGCTGATAC CGATGCTGTA GTCAACAGAT ATTGTGCCGT
Tri8-nsm
1901 GGGGGCCGAC ATTCACTTCG AAAGGAATAC TATCGGCGGG CACTACCAGG AAGCCGACAA TAGCTATGAG GCGGCTTTTC AGTGGCTTTT GGACATCTAC
Tri8-nsm
2001 TCCGGCCAGC GTGACACAAA GGGTTGTGTC ATCAAGGAAG TGACGAGAAA CATTACCGGA AGTGTCCTAC AAACACGTGA GAATGTTCAA AAGAGTGGAG
Tri8_Downstream
Tri8-nsm
2101 TTGACTTCTG GAGATCTGCC TGGTAATTTC ATTTCTTGAC ATCTCTGGCC ATCGGCCTGG TAATTTATTT TTGGACTGCC GGTCATCGAC TTGGTACGCC
Tri8_Downstream
2201 ATCCCTTATA TGTTTGGCCA GAACCTGTAG TGGTCTTATA CTGTGTCTTA TAAAATATTT ATTGATCCTT TTCTATGAGT AACTTTATCT GTCTCCGGCG
Tri8_Downstream
2301 ATGTAGCTGC ATCTGTAAGA TTCAAATAAA TCATCCAAAC ACCAGCCAGG TAGCATTAAT CGAAGAGGAC CTCCAATCTG AATAACTTCG GTGTTGTTGA
Tri8_Downstream
2401 GTATTCCTGG TTTATTGAGA TGGCTTTGAT CAGCTCCCAT TTCCCTTCAA GTTCCCTCCA TTGTGGTCAG AAGACAAGGG AAGCATGAGG GTGGACTCAT
Tri8_Downstream
2501 TTGAAGCACG ATAATTACAC TATCATAAAA CACAGGTTTA GCTTGAACTG AAAAACCACT CATCACATTC ACAGTAGCAC CACTGGATAG ATGTTGGGTT
Tri8_Downstream
2601 CAAGTAAGCC TCATGTTGCT CTGGCATCGC TACTTACCCC TTGCCGCAAC AATATGCATC TTTTACACTT CATGCACAAA CAAGGGCATG GTATCGTTGA
Tri8_Downstream
2701 GGCAATATCA GACATATAAA TTAATTATAA TATTGTCGGA TACTGCGTGG AGGAAGGCGA CTTCGTCTTT ATGGACGTCA AGCCCTCCTG TAAGCCTCTT
Tri8_Downstream
2801 TTCAAGGTCC CTCAGGCTCG CAAGCTCCTT CTCAAGCTCT GGCACTATCC GGCCTAGCTT CTTAGATCGA AAGCGCCTTA CGAAGAGCAA GAGGACCTTG
Tri8_Downstream
2901 TAGCTGAAGG CAATTAACCT GATGAGGCTG TAGTTATCGT CATATGGCCC CTCTCGGAGG CACCTTACCT TATGCCTTAG CTACTTTAGA TCCTTACTGT
Tri8_Downstream
3001 AGTTACTAAG TAAGATAACC TGGTTTATGC CTTCTTTAGT AAAGAAATAG TCTTCTAGAT AGCTAGGGAT ATAACTGTAT AGGCTTTTTT ATTGAGGCTG
Tri8_Downstream
Vb_S
3101 TATTATTATA TAATACCTAT GACATCGGTT AGCTATATGG AGCGATGGAC TGGGGTACCG AGCTCGAATT CGTAATCATG GTCATAGCTG TTTCCTGTGT
dTri8-D_AS
3201 GAAATTGTTA TCCGCTCACA ATTCCACACA ACATACGAGC CGGAAGCATA AAGTGTAAAG CCTGGGGTGC CTAATGAGTG AGCTAACTCA CATTAATTGC
3301 GTTGCGCTCA CTGCCCGCTT TCCAGTCGGG AAACCTGTCG TGCCAGCTGC ATTAATGAAT CGGCCAACGC GCGGGGAGAG GCGGTTTGCG TATTGGGCGC
3401 TCTTCCGCTT CCTCGCTCAC TGACTCGCTG CGCTCGGTCG TTCGGCTGCG GCGAGCGGTA TCAGCTCACT CAAAGGCGGT AATACGGTTA TCCACAGAAT
3501 CAGGGGATAA CGCAGGAAAG AACATGTGAG CAAAAGGCCA GCAAAAGGCC AGGAACCGTA AAAAGGCCGC GTTGCTGGCG TTTTTCCATA GGCTCCGCCC
3601 CCCTGACGAG CATCACAAAA ATCGACGCTC AAGTCAGAGG TGGCGAAACC CGACAGGACT ATAAAGATAC CAGGCGTTTC CCCCTGGAAG CTCCCTCGTG
3701 CGCTCTCCTG TTCCGACCCT GCCGCTTACC GGATACCTGT CCGCCTTTCT CCCTTCGGGA AGCGTGGCGC TTTCTCATAG CTCACGCTGT AGGTATCTCA
3801 GTTCGGTGTA GGTCGTTCGC TCCAAGCTGG GCTGTGTGCA CGAACCCCCC GTTCAGCCCG ACCGCTGCGC CTTATCCGGT AACTATCGTC TTGAGTCCAA
3901 CCCGGTAAGA CACGACTTAT CGCCACTGGC AGCAGCCACT GGTAACAGGA TTAGCAGAGC GAGGTATGTA GGCGGTGCTA CAGAGTTCTT GAAGTGGTGG
4001 CCTAACTACG GCTACACTAG AAGGACAGTA TTTGGTATCT GCGCTCTGCT GAAGCCAGTT ACCTTCGGAA AAAGAGTTGG TAGCTCTTGA TCCGGCAAAC
4101 AAACCACCGC TGGTAGCGGT GGTTTTTTTG TTTGCAAGCA GCAGATTACG CGCAGAAAAA AAGGATCTCA AGAAGATCCT TTGATCTTTT CTACGGGGTC
4201 TGACGCTCAG TGGAACGAAA ACTCACGTTA AGGGATTTTG GTCATGAGAT TATCAAAAAG GATCTTCACC TAGATCCTTT TAAATTAAAA ATGAAGTTTT
4301 AAATCAATCT AAAGTATATA TGAGTAAACT TGGTCTGACA GTTACCAATG CTTAATCAGT GAGGCACCTA TCTCAGCGAT CTGTCTATTT CGTTCATCCA
bla
4401 TAGTTGCCTG ACTCCCCGTC GTGTAGATAA CTACGATACG GGAGGGCTTA CCATCTGGCC CCAGTGCTGC AATGATACCG CGAGACCCAC GCTCACCGGC
bla
4501 TCCAGATTTA TCAGCAATAA ACCAGCCAGC CGGAAGGGCC GAGCGCAGAA GTGGTCCTGC AACTTTATCC GCCTCCATCC AGTCTATTAA TTGTTGCCGG
bla
4601 GAAGCTAGAG TAAGTAGTTC GCCAGTTAAT AGTTTGCGCA ACGTTGTTGC CATTGCTACA GGCATCGTGG TGTCACGCTC GTCGTTTGGT ATGGCTTCAT
bla
4701 TCAGCTCCGG TTCCCAACGA TCAAGGCGAG TTACATGATC CCCCATGTTG TGCAAAAAAG CGGTTAGCTC CTTCGGTCCT CCGATCGTTG TCAGAAGTAA
bla
4801 GTTGGCCGCA GTGTTATCAC TCATGGTTAT GGCAGCACTG CATAATTCTC TTACTGTCAT GCCATCCGTA AGATGCTTTT CTGTGACTGG TGAGTACTCA
bla

4901 ACCAAGTCAT TCTGAGAATA GTGTATGCGG CGACCGAGTT GCTCTTGCCC GGCGTCAATA CGGGATAATA CCGCGCCACA TAGCAGAACT TTAAAAGTGC
bla

5001 TCATCATTGG AAAACGTTCT TCGGGGCGAA AACTCTCAAG GATCTTACCG CTGTTGAGAT CCAGTTCGAT GTAACCCACT CGTGCACCCA ACTGATCTTC
bla

5101 AGCATCTTTT ACTTTCACCA GCGTTTCTGG GTGAGCAAAA ACAGGAAGGC AAAATGCCGC AAAAAAGGGA ATAAGGGCGA CACGGAAATG TTGAATACTC
bla

5201 ATACTCTTCC TTTTTCAATA TTATTGAAGC ATTTATCAGG GTTATTGTCT CATGAGCGGA TACATATTTG AATGTATTTA GAAAAATAAA CAAATAGGGG
bla

5301 TTCCGCGCAC ATTTCCCCGA AAAGTGCCAC CTGACGTCTA AGAAACCATT ATTATCATGA CATTAACCTA TAAAAATAGG CGTATCACGA GGCCCTTTCG

5401 TCTCGCGCGT TTCGGTGATG ACGGTGAAAA CCTCTGACAC ATGCAGCTCC CGGAGACGGT CACAGCTTGT CTGTAAGCGG ATGCCGGGAG CAGACAAGCC

5501 CGTCAGGGCG CGTCAGCGGG TGTTGGCGGG TGTCGGGGCT GGCTTAACTA TGCGGCATCA GAGCAGATTG TACTGAGAGT GCACCATATG CGGTGTGAAA

5601 TACCGCACAG ATGCGTAAGG AGAAAATACC GCATCAGGCG CCATTCGCCA TTCAGGCTGC GCAACTGTTG GGAAGGGCGA TCGGTGCGGG CCTCTTCGCT

5701 ATTACGCCAG CTGGCGAAAG GGGGATGTGC TGCAAGGCGA TTAAGTT

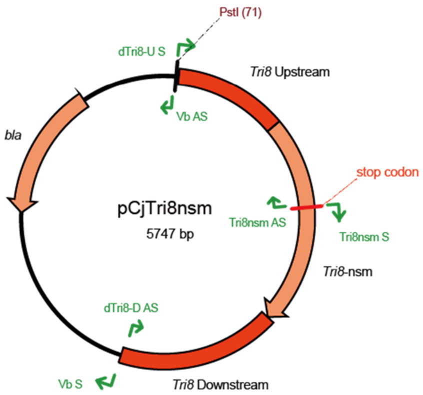

SbfI
Tri3_Upstream
dTri3-U_S
1 GGGTAACGCC AGGGTTTTCC CAGTCACGAC GTTGTAAAAC GACGGCCAGT GCCAAGCTTG CATGCCTGCA GGTCGACTCT AGAGACCTTT TAAGGGAGTA
Vb_AS

Tri3_Upstream
dTri3-U_S
101 ACGATATTGA GTTCGAATTT ATTAAGATAG ATACCATACA TAGATATCTG GTAATACAGA TGGTCATTGT CTCCATTATA AGAAGGCTGC ATAATAATAC

Tri3_Upstream
201 TTTAACGAGA GTTTCGGCGA TCTGATGGAG TAAGCACGTG GGGCTACATG TGGATTCATC TGGCGCTTTG TATATTAAAT TATCTAAACC CGAGCATTAT

Tri3_Upstream
301 GTTATACCTC TTTTCGTCGT AGGTTGCTTC ATGATACTGA GATACAAGAA TGATCCCTTC CCTGACTTGT GGTGCCGCTG GAAACTGAAG GAATGAAGAA

Tri3_Upstream
401 CCGACATTTA CAACAAACGT TGCATTTTCG CCATTGTGCC CTCCTAGATG TTTCGACTCA AGTCTGTAAC GTATTGAGAT GAACCAATGT GTGTCACTAA

Tri3_Upstream
501 ACTGAATCCT TGTCGAAAAA CTTGAATACA TGATGGAATG GCAAAAGTAG GTAAGAGATG CAAGGATAAG TGCTTGATCC GTCCGCTTAT GCCAGCCAGA

Tri3_Upstream
601 CTAGACTAAG AATACGTCAA ACTTTGAACA AGTTAAGGCG TATCGTGTCA GTGGGGGTTG TGTAAGGCCC AACAGTGCAT TGTAAGCTAA GACTCTCAGT

Tri3_Upstream
701 GCCTCAACTC CCTGTAAGTC TACAACATTT GTTGGCTCGG ATTGCGAAAC AAGGCTTCTA TTTATTAATA TAACTTGACA GCCAAGTATC TTCTGAGCTG

hphtkC2_AS
Tri3_Upstream
801 TTCTAATAAC ACAGAGCTCT GATTTATCTT TGATTCTACA GACAAAAAAA AGCAAGCAAC TTATTTCATC CTACAACCAT CAAGGTCAGT TAGCCTCCCC
dTri3-U_AS2
HSVtk

hphtkC2_AS
901 CATCTCCCGG GCAAACGTGC GCGCCAGGTC GCATATCGTC GGTATGGAGC CGGGGGTGGT GACGTGGGTC TGGACCATCC CGGAGGTAAG TTGCAGCAGG
HSVtk

1001 GCGTCCCGGC AGCCGGCGGG CGATTGGTCG TAATCCAGGA TAAAGACGTG CATGGAACGG AGGCGTTTGG CCAAGACGTC CAAGGCCCAG GCAAACACGT
HSVtk

1101 TATACAGGTC GCCGTTGGGG GCCAGCAACT CGGGGGCCCG AAACAGGGTA AATAACGTGT CCCCGATATG GGGTCGTGGG CCCGCGTTGC TCTGGGGCTC
HSVtk

1201 GGCACCCTGG GGCGGCACGG CCGTCCCCGA AAGCTGTCCC CAGTCCTCCC GCCACGACCC GCCGCACTGC AGATACCGCA CCGTATTGGC AAGTAGCCCG
HSVtk

1301 TAAACGCGGC GAATCGCAGC CAGCATAGCC AGGTCCAGCC GCTCGCCGGG GCGCTGGCGT TTGGCCAGGC GGTCGATGTG TCTGTCCTCC GGAAGGGCCC
HSVtk

1401 CAAGCACGAT GTTGGTGCCG GGCAAGGTCG GCGGGATGAG GGCCACGAAC GCCAGCACGG CCTGGGGGGT CATGCTGCCC ATAAGGTACC GCGCGGCCGG
HSVtk

1501 GTAGCACAGG AGGGCGGCGA TGGGATGGCG GTCGAAGATG AGGGTGAGGG CCGGGGGCGG GGCATGTGAG CTCCCAGCCT CCCCCCCGAT ATGAGGAGCC
HSVtk

1601 AGAACGGCGT CGGTCACGGC ATAAGGCATG CCCATTGTTA TCTGGGCGCT TGTCATTACC ACCGCCGCGT CCCCGGCCGA TATCTCACCC TGGTCGAGGC
HSVtk

1701 GGTGTTGTGT GGTGTAGATG TTCGCGATTG TCTCGGAAGC CCCCAGCACC CGCCAGTAAG TCATCGGCTC GGGTACGTAG ACGATATCGT CGCGCGAACC
HSVtk

1801 CAGGGCCACC AGCAGTTGCG TGGTGGTGGT TTTCCCCATC CCGTGGGGAC CGTCTATATA AACCCGCAGT AGCGTGGGCA TTTTCTGCTC CGGGCGGACT
HSVtk

1901 TCCGTGGCTT CTTGCTGCCG GCGAGGGCGC AACGCCGTAC GTCGGTTGCT ATGGCCGCGA GAACGCGCAG CCTGGTCGAA CGCAGACGCG TGTTGATGGC
HSVtk

2001 CGGGGTACGA AGCCATTTCC TTTGCCCTCG GACGAGTGCT GGGGCGTCGG TTTCCACTAT CGGCGAGTAC TTCTACACAG CCATCGGTCC AGACGGCCGC
HSVtk
hph

2101 GCTTCTGCGG GCGATTTGTG TACGCCCGAC AGTCCCGGCT CCGGATCGGA CGATTGCGTC GCATCGACCC TGCGCCCAAG CTGCATCATC GAAATTGCCG
hph

2201 TCAACCAAGC TCTGATAGAG TTGGTCAAGA CCAATGCGGA GCATATACGC CCGGAGGCGC GGCGATCCTG CAAGCTCCGG ATGCCTCCGC TCGAAGTAGC
hph

2301 GCGTCTGCTG CTCCATACAA GCCAACCACG GCCTCCAGAA GAGGATGTTG GCGACCTCGT ATTGGGAATC CCCGAACATC GCCTCGCTCC AGTCAATGAC
hph

2401 CGCTGTTATG CGGCCATTGT CCGTCAGGAC ATTGTTGGAG CCGAAATCCG CATGCACGAG GTGCCGGACT TCGGGGCAGT CCTCGGCCCA AAGCATCAGC
hph

2501 TCATCGAGAG CCTGCGCGAC GGACGCACTG ACGGTGTCGT CCATCACAGT TTGCCAGTGA TACACATGGG GATCAGCAAT CGCGCATATG AAATCACGCC
hph

2601 ATGTAGTGTA TTGACCGATT CCTTGCGGTC CGAATGGGCC GAACCCGCTC GTCTGGCTAA GATCGGCCGC AGCGATCGCA TCCATGGCCT CCGCGACCGG
hph

2701 CTGGAGAACA GCGGGCAGTT CGGTTTCAGG CAGGTCTTGC AACGTGACAC CCTGTGCACG GCGGGAGATG CAATAGGTCA GGCTCTCGCT GAACTCCCCA
hph

2801 ATGTCAAGCA CTTCCGGAAT CGGGAGCGCG GCCGATGCAA AGTGCCGATA AACATAACGA TCTTTGTAGA AACCATCGGC GCAGCTATTT ACCCGCAGGA
hph

2901 CATATCCACG CCCTCCTACA TCGAAGCTGA AAGCACGAGA TTCTTCGCCC TCCGAGAGCT GCATCAGGTC GGAGACGCTG TCGAACTTTT CGATCAGAAA
hph

3001 CTTCTCGACA GACGTCGCGG TGAGTTCAGG CTTTTTCATA TCGATCTCAA GCGGGGTAGC TGTTAGTCAA GCTGCGATGA AGTGGGAAAG CTCGAACTGA
hph GPD promoter

3101 AAGGTTCAAA GGAATAAGGG ATGGGAAGGA TGGAGTATGG ATGTAGCAAA GTACTTACTT AGGGGAAATA AAGGTTCTTG GATGGGAAGA TGAATATACT
GPD promoter

3201 GAAGATGGGA AAAGAAAGAG AAAAGAAAAG AGCAGCTGGT GGGGAGAGCA GGAAAATATG GCAACAAATG TTGGACTGAC GCAACGACCT TGTCAACCCC
GPD promoter

3301 GCCGACACAC CGGGCGGACA GACGGGGCAA AGCTGCCTAC CAGGGACTGA GGGACCTCAG CAGGTCGAGT GCAGAGCACC GGATGGGTCG ACTGCCAGCT
GPD promoter

3401 TGTGTTCCCG GTCTGCGCCG CTGGCCAGCT CCTGAGCGGC CTTTCCGGTT TCATACACCG GGCAAAGCAG GAGAGGCACG ATATTTGGAC GCCCTACAGA
GPD promoter

3501 TGCCGGATGG GCCAATTAGG GAGCTTACGC GCCGGGTACT CGCTCTACCT ACTTCGGAGA AGGTACTATC TCGTGAATCT TTTACCAGAT CGGAAGCAAT
GPD promoter

3601 TGGACTTCTG TACCTAGGTT AATGGCATGC TATTTCGCCG ACGGCTATAC ACCCCTGGCT TCACATTCTC CTTCGCTTAC TGCCGGTGAT TCGATGAAGC
GPD promoter

3701 TCCATATTCT CCGATGATGC AATAGATTCT TGGTCAACGA GGGGCACACC AGCCTTTCCA CTTCGGGGCG GAGGGGCGGC CGGTCCCGGA TTAATAATCA
GPD promoter

dTri3-D_S2
3801 TCCACTGCAC CTCAGAGCCG CCAGAGCTGT CTGGCGCAGT GGCGCTTATT ACTCAGCCCT TCTCTCTGCG TCCGTCCGTC TCTCCGCATG CCAGAAAGAG
GPD promoter
hphtkC2_S

Tri3_Downstream
dTri3-D_S2
3901 TCACCGGGGG TTTGCTGTTT ATTCTGAATG GTACATAGGT TTCTCGAGGA GCGATATTGT CAAGGCAACA CTTTGGGGTG GACTTTGAGT TCGGTGGTAC
GPD promoter
hphtkC2_S

Tri3_Downstream
4001 TCCAAGGACA GGAAGACATT ACATATGAGC TAGAACTGGA TGGGTAAATG TAGTTTGAAT ATAAATGTAT TTGGAAATTT GATTAGAATT TACCTATTTA

Tri3_Downstream
4101 CAATTCAATT TTTTACTTCC CAAACTATGA CTTATGTGGC TTGATATTAG ATCTTGGACA GGCCAGAGCC GAACTTCTAT AATTACAACT GCCTCTAGTT

Tri3_Downstream
4201 TCCACCAGGC CATTTTTACA TGCAATGAGC TTCGTTATAT GTATCAGCGC TCGTAGTATC AGTTCTCAAC AACACAAAAA TAAGAGTGAG GAAGTCCCAG

Tri3_Downstream
4301 GTGAACAGTT TTCTAGGTTC AGAAAAGAGT ATAGATACTA ATTTGGTGAA GACACATAGT CAACAGAAAC TTGCAGTTGA CTTTCTATAT GCTAAACTAC

Tri3_Downstream
4401 AAGATGAAGC CCAGTCCGTC TTGATGGACA ATCGCGATGT TCAACTGTGA GGTTAATGGT TTACATGTAT CAAACACAAT TGATTGTGCC CTTAAAGCTA

Tri3_Downstream
4501 ACATGAGTGG CCTTCACAAG TAACCTACAT GTCCAAAGCA GTTGATGTAT GTAAATAATA CTTAAGCTTA TTCTAAGTAA AGACATATTA TTAGTAATGC

Tri3_Downstream
4601 AGAGAAGACT TCCACAAGGA GTTAGTTTGA TAGAATGGTC ACAAGTGTAC GGTCTGTTAT TCGTCCCACA ATACATTGCC TGAGATGAAT AGTGTTTAAA

Tri3_Downstream
4701 TTGACGAGCG TCTAAATAGG GTACAATTTA TTTTACGTTC AGATGTCGCT ACTATGACCT TCCATCCTTG CATACCAAAA ATGGACTGAA CAAAAGGAAC

Vb_S
Tri3_Downstream
4801 AGGATTCGAT GCCGATACAG TGAGTTATCC AGTAAGATAT TGAGACAGTT TGTCAGATGC GCCGGGGTAC CGAGCTCGAA TTCGTAATCA TGGTCATAGC
dTri3-D_AS

4901 TGTTTCCTGT GTGAAATTGT TATCCGCTCA CAATTCCACA CAACATACGA GCCGGAAGCA TAAAGTGTAA AGCCTGGGGT GCCTAATGAG TGAGCTAACT
5001 CACATTAATT GCGTTGCGCT CACTGCCCGC TTTCCAGTCG GGAAACCTGT CGTGCCAGCT GCATTAATGA ATCGGCCAAC GCGCGGGGAG AGGCGGTTTG
5101 CGTATTGGGC GCTCTTCCGC TTCCTCGCTC ACTGACTCGC TGCGCTCGGT CGTTCGGCTG CGGCGAGCGG TATCAGCTCA CTCAAAGGCG GTAATACGGT
5201 TATCCACAGA ATCAGGGGAT AACGCAGGAA AGAACATGTG AGCAAAAGGC CAGCAAAAGG CCAGGAACCG TAAAAAGGCC GCGTTGCTGG CGTTTTTCCA
5301 TAGGCTCCGC CCCCCTGACG AGCATCACAA AAATCGACGC TCAAGTCAGA GGTGGCGAAA CCCGACAGGA CTATAAAGAT ACCAGGCGTT TCCCCCTGGA
5401 AGCTCCCTCG TGCGCTCTCC TGTTCCGACC CTGCCGCTTA CCGGATACCT GTCCGCCTTT CTCCCTTCGG GAAGCGTGGC GCTTTCTCAT AGCTCACGCT
5501 GTAGGTATCT CAGTTCGGTG TAGGTCGTTC GCTCCAAGCT GGGCTGTGTG CACGAACCCC CCGTTCAGCC CGACCGCTGC GCCTTATCCG GTAACTATCG
5601 TCTTGAGTCC AACCCGGTAA GACACGACTT ATCGCCACTG GCAGCAGCCA CTGGTAACAG GATTAGCAGA GCGAGGTATG TAGGCGGTGC TACAGAGTTC
5701 TTGAAGTGGT GGCCTAACTA CGGCTACACT AGAAGGACAG TATTTGGTAT CTGCGCTCTG CTGAAGCCAG TTACCTTCGG AAAAAGAGTT GGTAGCTCTT
5801 GATCCGGCAA ACAAACCACC GCTGGTAGCG GTGGTTTTTT TGTTTGCAAG CAGCAGATTA CGCGCAGAAA AAAAGGATCT CAAGAAGATC CTTTGATCTT
5901 TTCTACGGGG TCTGACGCTC AGTGGAACGA AAACTCACGT TAAGGGATTT TGGTCATGAG ATTATCAAAA AGGATCTTCA CCTAGATCCT TTTAAATTAA
6001 AAATGAAGTT TTAAATCAAT CTAAAGTATA TATGAGTAAA CTTGGTCTGA CAGTTACCAA TGCTTAATCA GTGAGGCACC TATCTCAGCG ATCTGTCTAT
bla

6101 TTCGTTCATC CATAGTTGCC TGACTCCCCG TCGTGTAGAT AACTACGATA CGGGAGGGCT TACCATCTGG CCCCAGTGCT GCAATGATAC CGCGAGACCC
bla

6201 ACGCTCACCG GCTCCAGATT TATCAGCAAT AAACCAGCCA GCCGGAAGGG CCGAGCGCAG AAGTGGTCCT GCAACTTTAT CCGCCTCCAT CCAGTCTATT
bla

6301 AATTGTTGCC GGGAAGCTAG AGTAAGTAGT TCGCCAGTTA ATAGTTTGCG CAACGTTGTT GCCATTGCTA CAGGCATCGT GGTGTCACGC TCGTCGTTTG
bla

6401 GTATGGCTTC ATTCAGCTCC GGTTCCCAAC GATCAAGGCG AGTTACATGA TCCCCCATGT TGTGCAAAAA AGCGGTTAGC TCCTTCGGTC CTCCGATCGT
bla

6501 TGTCAGAAGT AAGTTGGCCG CAGTGTTATC ACTCATGGTT ATGGCAGCAC TGCATAATTC TCTTACTGTC ATGCCATCCG TAAGATGCTT TTCTGTGACT
bla

```
6601  GGTGAGTACT CAACCAAGTC ATTCTGAGAA TAGTGTATGC GGCGACCGAG TTGCTCTTGC CCGGCGTCAA TACGGGATAA TACCGCGCCA CATAGCAGAA
                                                  bla
6701  CTTTAAAAGT GCTCATCATT GGAAAACGTT CTTCGGGGCG AAAACTCTCA AGGATCTTAC CGCTGTTGAG ATCCAGTTCG ATGTAACCCA CTCGTGCACC
                                                  bla
6801  CAACTGATCT TCAGCATCTT TTACTTTCAC CAGCGTTTCT GGGTGAGCAA AAACAGGAAG GCAAAATGCC GCAAAAAAGG GAATAAGGGC GACACGGAAA
                                                  bla
6901  TGTTGAATAC TCATACTCTT CCTTTTTCAA TATTATTGAA GCATTTATCA GGGTTATTGT CTCATGAGCG GATACATATT TGAATGTATT TAGAAAAATA
          bla
7001  AACAAATAGG GGTTCCGCGC ACATTTCCCC GAAAAGTGCC ACCTGACGTC TAAGAAACCA TTATTATCAT GACATTAACC TATAAAAATA GGCGTATCAC
7101  GAGGCCCTTT CGTCTCGCGC GTTTCGGTGA TGACGGTGAA AACCTCTGAC ACATGCAGCT CCCGGAGACG GTCACAGCTT GTCTGTAAGC GGATGCCGGG
7201  AGCAGACAAG CCCGTCAGGG CGCGTCAGCG GGTGTTGGCG GGTGTCGGGG CTGGCTTAAC TATGCGGCAT CAGAGCAGAT TGTACTGAGA GTGCACCATA
7301  TGCGGTGTGA AATACCGCAC AGATGCGTAA GGAGAAAATA CCGCATCAGG CGCCATTCGC CATTCAGGCT GCGCAACTGT TGGGAAGGGC GATCGGTGCG
7401  GGCCTCTTCG CTATTACGCC AGCTGGCGAA AGGGGGATGT GCTGCAAGGC GATTAAGTT
```

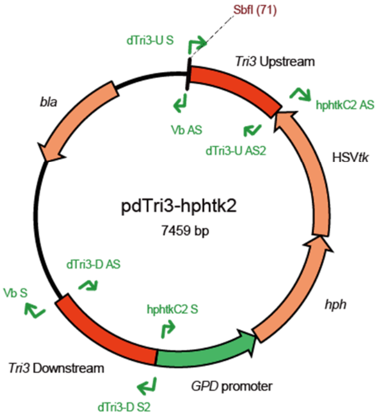

Supplementary Figure S7. Transgenic *F. graminearum* strains used for the investigation of the effect of *Tri6* and *Tri10* overexpression on 15-deCAL accumulation. (A) Pedigree of transformants with multiple genetic manipulations for the generation of a *Tri3*-disrupted strain, YN_153, with overexpression of *Tri6* and *Tri10*. The gene disruptants, YN_145 (Δ*Tri6* / P_{TEF}::*Tri6*, ΔP_{Tri10} / P_{GPD}, Δ*Tri8* / P_{GPD}::*hph*::*tk*) and YN_153 (Δ*Tri6* / P_{TEF}::*Tri6*, ΔP_{Tri10} / P_{GPD}, Δ*Tri8* / *Tri8_nsm*, Δ*Tri3* / P_{GPD}::*hph*::*tk*), were obtained from the marker-free transformants YN_120 (**Supplementary Figure S6**) and YN_149 (Δ*Tri6* / P_{TEF}::*Tri6*, ΔP_{Tri10} / P_{GPD}, Δ*Tri8* / *Tri8_nsm*), respectively, by positive selection. YN_149 was created from YN_145 by negative selection. (B) Generation of strain YN_153. YN_145 was created from YN_120 by positive selection with hygromycin B following transformation with pdjTri8-hphtk. YN_149 was created from YN_145 by negative selection with 5-FdU following transformation with pCjTri8-nsm. YN153 was created from YN_149 by positive selection with hygromycin B after transformation with pdjTri3-hphtk2. (C) Sequences and maps of disruption and replacement vectors.

References

1. Kimura, M.; Kamakura, T.; Tao, Q.Z.; Kaneko, I.; Yamaguchi, I. Cloning of the blasticidin S deaminase gene (*BSD*) from *Aspergillus terreus* and its use as a selectable marker for *Schizosaccharomyces pombe* and *Pyricularia oryzae*. *Mol. Gen. Genet.* **1994**, *242*, 121-129. DOI:10.1007/bf00391004.
2. Banno, S.; Kimura, M.; Tokai, T.; Kasahara, S.; Higa-Nishiyama, A.; Takahashi-Ando, N.; Hamamoto, H.; Fujimura, M.; Staskawicz, B.J.; Yamaguchi, I. Cloning and characterization of genes specifically expressed during infection stages in the rice blast fungus. *FEMS. Microbiol. Lett.* **2003**, *222*, 221-227. DOI:10.1016/s0378-1097(03)00307-0.
3. Maeda, K.; Tanaka, A.; Sugiura, R.; Koshino, H.; Tokai, T.; Sato, M.; Nakajima, Y.; Tanahashi, Y.; Kanamaru, K.; Kobayashi, T.; Nishiuchi, T.; Fujimura, M.; Takahashi-Ando, N.; Kimura, M. Hydroxylations of trichothecene rings in the biosynthesis of *Fusarium* trichothecenes: evolution of alternative pathways in the nivalenol chemotype. *Environ. Microbiol.* **2016**, *18*, 3798-3811. DOI:10.1111/1462-2920.13338.
4. Tanaka, A.; Saikawa, S.; Suzuki, T.; Echigo, A.; Maeda, K.; Sato, M.; Fujimura, M.; Tokai, T.; Usami, R.; Yoshida, Y.; Kimura, M.; Takahashi-Ando, N. Acetyltransferase activity in *Pseudomonas* sp. capable of acetylating the C-4 hydroxyl group of nivalenol-type trichothecenes. *J. Gen. Appl. Microbiol.* **2017**, *62*, 326-329. DOI:10.2323/jgam.2016.05.002.
5. Maeda, K.; Tanaka, Y.; Matsuyama, M.; Sato, M.; Sadamatsu, K.; Suzuki, T.; Matsui, K.; Nakajima, Y.; Tokai, T.; Kanamaru, K.; Ohsato, S.; Kobayashi, T.; Fujimura, M.; Nishiuchi, T.; Takahashi-Ando, N.; Kimura, M. Substrate specificities of *Fusarium* biosynthetic enzymes explain the genetic basis of a mixed chemotype producing both deoxynivalenol and nivalenol-type trichothecenes. *Int. J. Food Microbiol.* **2020**, *320*, 108532. DOI:10.1016/j.ijfoodmicro.2020.108532.
6. Maeda, K.; Nakajima, Y.; Koizumi, Y.; Takahashi-Ando, N.; Kimura, M.; Ohsato, S. Accumulation of 4-deoxy-7-hydroxytrichothecenes, but not 4,7-dihydroxytrichothecenes, in axenic culture of a transgenic nivalenol chemotype expressing the NX-type *FgTri1* gene. *Int. J. Mol. Sci.* **2021**, *22*, 11428. DOI:10.3390/ijms222111428.
7. Tokai, T.; Koshino, H.; Kawasaki, T.; Igawa, T.; Suzuki, Y.; Sato, M.; Fujimura, M.; Eizuka, T.; Watanabe, H.; Kitahara, T.; Ohta, K.; Shibata, T.; Kudo, T.; Inoue, H.; Yamaguchi, I.; Kimura, M. Screening of putative oxygenase genes in the *Fusarium graminearum* genome sequence database for their role in trichothecene biosynthesis. *FEMS Microbiol. Lett.* **2005**, *251*, 193-201. DOI:10.1016/j.femsle.2005.07.043.

8. Tokai, T.; Koshino, H.; Takahashi-Ando, N.; Sato, M.; Fujimura, M.; Kimura, M. *Fusarium Tri4* encodes a key multifunctional cytochrome P450 monooxygenase for four consecutive oxygenation steps in trichothecene biosynthesis. *Biochem Biophys Res Commun* **2007**, *353*, 412-417. DOI:10.1016/j.bbrc.2006.12.033.
9. Nakajima, Y.; Tokai, T.; Maeda, K.; Tanaka, A.; Takahashi-Ando, N.; Kanamaru, K.; Kobayashi, T.; Kimura, M. A set of heterologous promoters useful for investigating gene functions in *Fusarium graminearum*. *JSM Mycotoxins* **2014**, *64*, 147-152. DOI:10.2520/myco.64.147.
10. Liew, M.X.X.; Nakajima, Y.; Maeda, K.; Kitamura, N.; Kimura, M. Regulatory mechanism of trichothecene biosynthesis in *Fusarium graminearum*. *Front. Microbiol.* **2023**, *14*, 1148771. DOI:10.3389/fmicb.2023.1148771.
